# Functional Family Therapy for families of youth (age 11–18) with behaviour problems: A systematic review and meta‐analysis

**DOI:** 10.1002/cl2.1324

**Published:** 2023-07-19

**Authors:** Julia H. Littell, Therese D. Pigott, Karianne H. Nilsen, Jennifer Roberts, Travis K. Labrum

**Affiliations:** ^1^ Graduate School of Social Work and Social Research Bryn Mawr College Bryn Mawr Pennsylvania USA; ^2^ School of Public Health Georgia State University Atlanta Georgia USA; ^3^ Regional Centre for Child and Adolescent Mental Health Eastern and Southern Norway (RBUP) Oslo Norway; ^4^ School of Social Sciences, Education and Social Work Queen's University Belfast Belfast UK; ^5^ School of Social Work University of Pittsburgh Pittsburgh Pennsylvania USA

## Abstract

**Background:**

Functional Family Therapy (FFT) is a short‐term family‐based intervention for youth with behaviour problems. FFT has been widely implemented in the USA and other high‐income countries. It is often described as an evidence‐based program with consistent, positive effects.

**Objectives:**

We aimed to synthesise the best available data to assess the effectiveness of FFT for families of youth with behaviour problems.

**Search Methods:**

Searches were performed in 2013–2014 and August 2020. We searched 22 bibliographic databases (including PsycINFO, ERIC, MEDLINE, Science Direct, Sociological Abstracts, Social Services Abstracts, World CAT dissertations and theses, and the Web of Science Core Collection), as well as government policy databanks and professional websites. Reference lists of articles were examined, and experts were contacted to search for missing information.

**Selection Criteria:**

We included randomised controlled trials (RCTs) and quasi‐experimental designs (QEDs) with parallel cohorts and statistical controls for between‐group differences at baseline. Participants were families of young people aged 11–18 with behaviour problems. FFT programmes were compared with usual services, alternative treatment, and no treatment. There were no publication, geographic, or language restrictions.

**Data Collection and Analysis:**

Two reviewers independently screened 1039 titles and abstracts, read all available study reports, assessed study eligibility, and extracted data onto structured electronic forms. We assessed risks of bias (ROB) using modified versions of the Cochrane ROB tool and the What Works Clearinghouse standards. Where possible, we used random effects models with inverse variance weights to pool results across studies. We used odds ratios for dichotomous outcomes and standardised mean differences for continuous outcomes. We used Hedges *g* to adjust for small sample sizes. We assessed the heterogeneity of effects with *χ* ^2^ and *I* ^2^. We produced separate forest plots for conceptually distinct outcomes and for different endpoints (<9, 9–14, 15–23, and 24–42 months after referral). We grouped studies by study design (RCT or QED), and then assessed differences between these two subgroups of studies with *χ* ^2^ tests. We generated robust variance estimates, using correlated effects (CE) models with small sample corrections to synthesise all available outcome data. Exploratory CE analyses assessed potential moderators of effects within these domains. We used GRADE guidelines to assess the certainty of evidence on six primary outcomes at 1 year after referral.

**Main Results:**

Twenty studies (14 RCTs and 6 QEDs) met our inclusion criteria. Fifteen of these studies provided some valid data for meta‐analysis; these studies included 10,980 families in relevant FFT and comparison groups. All included studies had high risks of bias on at least one indicator. Half of the studies had high risks of bias on baseline equivalence, support for intent‐to‐treat analysis, selective reporting, and conflicts of interest. Fifteen studies had incomplete reporting of outcomes and endpoints. Using the GRADE rubric, we found that the certainty of evidence for FFT was very low for all of our primary outcomes. Using pairwise meta‐analysis, we found no evidence of effects of FFT compared with other active treatments on any primary or secondary outcomes. Primary outcomes were: recidivism, out‐of‐home placement, internalising behaviour problems, external behaviour problems, self‐reported delinquency, and drug or alcohol use. Secondary outcomes were: peer relations and prosocial behaviour, youth self esteem, parent symptoms and behaviour, family functioning, school attendance, and school performance. There were few studies in the pairwise meta‐analysis (*k* < 7) and little heterogeneity of effects across studies in most of these analyses. There were few differences between effect estimates obtained in RCTs versus QEDs. More comprehensive CE models showed positive results of FFT in some domains and negative results in others, but these effects were small (standardised mean difference [SMD] <|0.20|) and not significantly different from no effect with one exception: Two studies found positive effects of FFT on youth substance abuse and two studies found null results in this domain, and the overall effect estimate for this outcome was statistically different from zero. Over all outcomes (15 studies and 293 effect sizes), small positive effects were detected (SMD = 0.19, SE = 0.09), but these were not significantly different from zero effect. Prediction intervals showed that future FFT evaluations are likely to produce a wide range of results, including moderate negative effects and strong positive results (−0.37 to 0.75).

**Authors’ Conclusions:**

Results of 10 RCTs and five QEDs show that FFT does not produce consistent benefits or harms for youth with behavioural problems and their families. The positive or negative direction of results is inconsistent within and across studies. Most outcomes are not fully reported, the quality of available evidence is suboptimal, and the certainty of this evidence is very low. Overall estimates of effects of FFT may be inflated, due to selective reporting and publication biases.

## 1 PLAIN LANGUAGE SUMMARY

### 1.1 Functional Family Therapy is not consistently more (or less) effective than other services

Functional Family Therapy (FFT) is a family‐based intervention for youth with behaviour problems. It is often described as an evidence‐based programme with consistent, positive effects. This systematic review finds that FFT does not produce consistent benefits or harms. The positive or negative direction of results is inconsistent within and across studies.

### 1.2 What is this review about?

FFT is a prominent, short‐term treatment for families of youth with behaviour problems that has been implemented in 45 states in the USA and in nine other high‐income countries. Proponents claim that FFT is consistently more effective than treatment as usual (TAU) and other treatments in improving outcomes for youth and families.

This review assessed the effectiveness of FFT in reducing recidivism, out‐of‐home placements, youth behaviour problems, delinquency, and substance use; and improving peer relations, self esteem, school outcomes, parent functioning, and family functioning.

**What is the aim of this review?**

This review synthesised data from the best available studies to assess the effectiveness of FFT compared with other active treatments and with no treatment.

### 1.3 What studies are included?

The review included rigorous evaluations of the impacts of FFT, including: randomised controlled trials and non‐randomised studies that used parallel cohorts and statistical controls for between‐group differences at baseline.

Eligible studies involved families of young people aged 11‐18 with behaviour problems, such as criminal offenses, delinquency, anti‐social behaviour, and substance abuse.

### 1.4 What are the findings of this review?

Twenty studies met the review's eligibility criteria, but only 15 provided valid data for meta‐analysis. All included studies had high risks of bias on at least one indicator. Half of the studies had high risks of bias on at least four indicators, and most (75%) had incomplete reporting of outcomes and endpoints.

The review found that FFT is not consistently more or less effective than the other treatments to which it has been compared, including various forms of TAU and individual, family, and group therapies. There is insufficient evidence to draw conclusions about the effects of FFT compared with no treatment.

The direction of FFT effects is mixed, sometimes positive, sometimes negative. Confidence intervals show that almost all pooled estimates of effects of FFT are not statistically different from zero. Prediction intervals indicate that future evaluations of FFT are likely to show a wide range of negative and positive effects.

### 1.5 What are the implications for research and policy?

The best available evaluations of FFT are small controlled trials and quasi‐experiments with incomplete reporting of outcomes and some high risks of bias. Future studies should have prospectively registered protocols, use larger samples and more rigorous research methods, and provide full reporting on all outcomes and end points.

Incomplete reporting of results of primary studies may have inflated overall estimates of positive effects of FFT.

Although FFT has been marketed as a ‘scientifically proven’, effective, evidence‐based practice, policymakers and practitioners should be aware that the certainty of the evidence for FFT is very low. The direction of results is inconsistent within and across studies, and there is no empirical support for claims that FFT is consistently more effective than other treatments.

Information on the cost‐effectiveness of FFT appears to be based on inflated estimates of the effects of treatment. Therefore, claims about the cost‐effectiveness of FFT are not convincing.

### 1.6 How up to date is this review?

The review authors searched for studies that were reported through August 2020.

## 2 BACKGROUND

### 2.1 Description of the condition

Behavioural problems are among the most commonly diagnosed problems in children. In 2003, 6.3% of children ages 6 to 17 in the USA were diagnosed with behavioural problems, yet rates of parental concerns about these problems were much higher, suggesting that child behaviour problems may be under‐diagnosed in this sample (Blanchard, 2006). Clinically relevant behaviour problems were identified in 10% to 18% of children and youth between the ages of 4 and 18 living in Germany in 2001 (Barkmann, 2005). In Turkey, 11.9% of 2‐ to 3‐year‐old children scored in the clinically significant range and 18.6% were in the borderline range on the Child Behaviour Checklist (Erol, 2005). A cross‐national study found few differences in children's internalising or externalising behaviour problems as a function of nationality, gender, or age (Lambert, 1994).

### 2.2 Description of the intervention

A number of family‐based interventions have been developed to prevent and/or treat behavioural problems among children and youth. Among these, Functional Family Therapy (FFT) is one of the oldest and most widely‐known.

FFT is a short‐term, family‐based intervention for youth with behavioural problems. Developed by James Alexander and others in the early 1970s, this model has been implemented with youth at risk for or presenting with behavioural problems such as delinquency, violence, substance abuse, conduct disorder, oppositional defiant disorder, or disruptive behaviour disorders. Substance use or abuse is thought to be prevalent among youth participating in these programmes (Alexander, 1998, p. 44). Participants have included court mandated referrals and chronic delinquents released from state institutions. FFT has been used as an alternative to incarceration or as a re‐entry program for youth returning to the community following release from institutional settings. It has been offered in mental health, juvenile justice, and child welfare settings. It has been widely used in the USA in both rural and urban areas, and has been deployed and studied in other countries (e.g., in the Netherlands, Norway, Ireland, Sweden, and the UK).

FFT reflects a core set of theoretical principles, in which behaviour is seen as a representation of the family relational system; that is, as indicative of the functionality of the family. The overarching goals of FFT are described by its developers as follows:

1. Changing the maladaptive behaviours of youth and families, especially those who at the outset may not be motivated or may not believe they can change;
2. Reducing the personal, societal, and economic consequences of disruptive behaviour disorders; and
3. Doing so with less cost, in terms of time and money, than many other treatments currently available (Alexander, 1998, p. 7).

FFT is a short‐term (90‐day), intensive and comprehensive program that can be delivered in clinical settings, school settings, or at home. Implemented by professional therapists, the program requires about 8–30 h of direct service to youth and their families over an average of 12 sessions in 90 days. FFT has five specific objectives: engagement, motivation, assessment, behaviour change, and generalisation. Intervention is structured so that these objectives are accomplished in phases. Each phase is built upon the previous phase and has an assessment and intervention component directed at specific goals.

Waldron and colleagues described FFT implementation in two phases: an engagement‐and‐motivation phase, followed by behaviour change (Waldron, 2001, p. 805). Other authors describe engagement and motivation as two distinct phases; some add an assessment phase in the middle (before behaviour change); and others add a generalisation phase at the end. Thus, FFT is described as having two to five phases. Questions can be raised about whether these phases are truly distinct (e.g., it is possible to delay assessment until the middle phase?).

**Engagement** involves ‘maximising factors which enhance the perception that positive change might occur (intervention credibility), and minimising factors (e.g., poor program image, difficult location, insensitive referral) that might signify insensitivity and/or inappropriate resources’ (Alexander, 1998; p. 15).

To develop or enhance family members’ **motivation**, workers ‘identify and quickly begin to modify the pattern of changeable intrafamily risk factors, especially negativity, hopelessness, and blaming; [and] initiate and/or strengthen intrafamilial protective factors that can mitigate the effect of risk factors that cannot be changed’ (Alexander, 1998, p. 15).

**Assessment** focuses on the functional nature of problems within the family, rather than a diagnosis. Assessment is a continuous, multilevel, multidimensional, and multi‐method process that includes individual, family, behavioural, and contextual factors (Alexander, 1998, p. 22). It focuses on the promotion and maintenance of problematic sequences (chains of behaviours, events, or interactions), identifying interrelationships, and identifying risk and protective factors.

The **behaviour change** phase is aimed at developing ‘long term behaviour change patterns that are culturally appropriate, context sensitive, and individualised to the unique characteristics of each family member’ (Alexander, 1998, p. 15). Workers focus on cognitive, interactive, and emotional issues; emphasise positive communication and parenting skills; and provide concrete resources that ‘guide and symbolise specific changes in behaviour’ (Alexander, 1998, p. 15). This phase aims to reduce intrafamilial risk factors and enhance intrafamilial protective factors.

The late phase focuses on **generalisation** of behaviour change to other settings and social systems. This involves mobilising community support systems and modifying deteriorated family‐system relationships (e.g., with schools, probation officers) (Alexander, 1998, p. 15).

As used in FFT, the term ‘family’ refers to a wide range of family forms and structures. It includes a variety of living arrangements, and often refers to a unit that includes a youth who resides with one or more adult figures (a parent or guardian) who are deemed responsible for the youth's conduct. ‘In general, FFT initiates intervention with the unit that represents the current reality for the identified youth’ (Alexander, 1998, p.16).

In recent years, specialty FFT programs have been developed to address the needs of special populations of children and youth. The FFT‐CW program treats children involved in the child welfare system, often due to problems with child abuse and neglect. The FFT‐G program was developed for youth at risk of involvement in gangs. The FFT‐G program is relevant for our purposes, as it serves youth in the same age group as FFT (in contrast, cases in FFT‐CW tend to include younger children).

The **FFT‐G** program includes all of the regular features of FFT, along with efforts to address pressure from neighbourhood gang members and to engage families of gang‐involved youth (Gottfredson, 2018, p. 940). Development of the FFT‐G model was supported with $750,000 USD in grants from the US Office of Juvenile Justice and Delinquency Prevention in 2009–2010. FFT LLC staff produced the manual for FFT‐G and provided training and initial supervision for FFT‐G therapists.

Successful marketing of FFT has made these programs eligible for federal funding in the USA under Medicaid and Title IV‐E. Thus, there are financial incentives to organisations to provide FFT.

### 2.3 How the intervention might work

As described above, FFT is supposed to work in phases, beginning with efforts to engage the target youth and their family members, who may be resistant to treatment. Next, FFT therapists try to build youth and family members’ motivations to change. Third, FFT workers and family members assess the family's strengths and problems. Fourth, they attempt to bring about behavioural changes that can improve youth, parent, and family functioning. And, finally, therapists and family members make efforts to sustain changes over time and to generalize these changes to other settings and social systems.

Therapists are expected to adapt FFT services to fit family members’ capacities and the specific problems they face. FFT uses reframing (redefining individual and family problems and strengths), interpretations of patterns of maladaptive behaviour with links to emotions, deepening understanding of actions, and communication training with focus on positive communication. It incorporates theories of information processing, social cognition, and the psychology of emotion (Alexander, 1998, p. 10).

The model is said to be useful for complex and multidimensional problems because of its flexible structure and alleged cultural sensitivity. Effectiveness is attributed to the careful sequencing of techniques, helped by the continuous assessment and intervention processes, organised in phases that build upon each other (Alexander, 1998).

FFT therapists are expected to have Masters’ degrees in psychology, counselling, marriage and family therapy, social work, or a related area (Alexander, 1998). FFT is usually implemented in ‘sites’ which are working groups of FFT trained professionals and support staff. It takes approximately 1 year to implement FFT in a new site. It is necessary to have a certificate to practice FFT, and FFT requires close training and supervision. There are four levels of certification: Functional Family therapist, FFT Clinical Team leader, FFT Clinical Supervisor, and FFT Trainer. Working FFT teams meet weekly with a FFT supervisor to discuss cases and clinical issues.

A training manual for FFT was developed by Thomas Sexton and colleagues (Sexton, 2004). Some studies suggest that the effectiveness of FFT depends on therapist competence (Barnoski, 2002) and the degree to which therapists adhere to the FFT model (Sexton, 2010); but, there are serious questions about the validity of available measures of competence and adherence, and one study found that adherence to FFT was not related to outcomes (Hollimon, 2004).

Average costs of FFT per family were estimated at $2140 in 2003 USD (Aos, 2004). More recent estimates put the average cost of FFT‐G at $2,417 USD per family ($154,718 for 64 families; Gottfredson, 2018, p. 947).

A cost–benefit analysis suggested that FFT might produce a net savings of over $14,315 USD per youth served in Washington State and $26,216 USD per youth outside of Washington State (Aos, 2001, 2004). However, this analysis was limited to a narrow range of outcomes and appears to rely solely on data from published reports, which may produce inflated estimates of treatment effects (Dwan, 2013, 2014; Norris, 2012; Pigott, 2013; Polanin, 2016; Song, 2010).

### 2.4 Why it is important to do this review

#### 2.4.1 Prior research

Proponents of FFT claim that it has ‘proven results’ in reducing recidivism among youthful offenders, reducing drug charges, and improving school and work attendence, resulting in cost savings of up to $5000 USD per family (https://www.fftllc.com/evidence-based-research). But these claims are based largely upon non‐systematic reviews of results of observational studies and a few, small controlled studies.

The effects of FFT have been the subject of outcome evaluations since 1970s. Alexander and colleagues (Alexander, 2013) identified 27 studies of the outcomes of FFT, based on 24 unique (non‐overlapping) samples. Most (25) of these studies were conducted in the USA (8 in Salt Lake City, Utah; 6 in Albuquerque, New Mexico; 3 in Ohio; 2 in Indiana; 2 in Washington state; 2 in Oregon; 1 in California, and 1 in Pennsylvania); 2 studies were conducted in Lund, Sweden.

Robbins and colleagues (Robbins, 2016) cited 15 RCTs in which FFT (or a similar program) was compared to alternative treatments and/or no treatment (of these, 10 RCTs were classified as efficacy trials and 5 were termed effectiveness studies). They also identified 12 non‐randomised dissemination/implementation studies, including some that lacked comparison groups.

Most FFT evaluations obtain measures of multiple outcomes, from different sources, and over multiple endpoints. Data on recidivism (arrest and conviction) and out‐of‐home placements are often obtained from police and court records, juvenile justice records, child welfare services, and/or hospital records. Data on youth, parent, and family functioning are usually obtained through structured interviews with youth and parents. These interviews tend to include standardised instruments, such as the Child Behaviour CheckList (CBCL; Achenbach, 1991), which measures internalising behaviours (e.g., depression, anxiety, somatization) and externalising behaviours (e.g., acting out, aggression, hostility, antisocial behaviour). Other commonly used standardised measures include the Self‐Reported Delinquency Scale (Elliot, 1985) and various measures of aspects of family functioning (e.g., cohesion, adaptability).

#### 2.4.2 Previous reviews

Many narrative summaries of research on the processes and outcomes of FFT have been produced by FFT program developers (e.g., Alexander, 1998, 2013; Robbins, 2016; Sexton, 2019) and others (e.g., Kazdin, 2015).

FFT trials have been included in larger meta‐analytic reviews of effects of interventions for juvenile offenders (Lipsey, 1998), family‐based treatments (Shadish, 2002), treatments for adolescent substance abuse (Stanton, 1997; Waldron, 2008; Tanner‐Smith, 2013), antisocial behaviour (Sawyer, 2015), and comparisons of residential versus non‐residential forms of care for youth (Gutterswijk, 2020); these larger reviews do not report separate results for FFT.

Weisman and Montgomery (Weisman, 2019) produced an overview of 31 reviews that were published between 1986 and 2018 and included at least one primary study of FFT. The overview includes narrative reviews as well as systematic reviews, reviews that focused only on FFT as well as those that included research on a wider array of interventions. Some of the reviews in the latter category did not provide analyses or estimates of outcomes of FFT per se.

Table 1 shows our analysis of previous systematic reviews and meta‐analyses that provided analyses of effects of FFT. Some of these reviews also included studies of other types of interventions, but they all provided pooled estimates of effects of FFT. We assessed each of these reviews using the initial (simple) version of AMSTAR (Assessment of Multiple SysTemAtic Reviews; Shea et al., 2007). Most reviews did not meet some AMSTAR criteria.

**Table 1:** Systematic reviews and meta‐analyses of research on outcomes of FFT, assessed with AMSTAR.

	Austin, 2005	Baldwin, 2012	Filges, Filges 2015, Filges 2018	Hartnett, 2017
Number of FFT studies included	1	3	2	14
Focal intervention(s)	Family‐based interventions	Family therapies	FFT	FFT
Target behaviours	Substance use problems	Delinquency and substance abuse	Non‐opiod drug use	Disruptive behaviours and substance use disorders
1. Was an ‘a priori’ design provided? The research question and inclusion criteria should be established before the conduct of the review.	No	No	Yes	No
2. Was there duplicate study selection and data extraction? There should be at least two independent data extractors and a consensus procedure for disagreements should be in place.	Unclear	Yes	Yes	Unclear
3. Was a comprehensive literature search performed? At least two electronic sources should be searched. The report must include years and databases used (e.g., Central, EMBASE, and MEDLINE). Key words and/or MESH terms must be stated and where feasible the search strategy should be provided. All searches should be supplemented by consulting current contents, reviews, textbooks, specialised registers, or experts in the particular field of study, and by reviewing references in studies found.	No	Unclear	Yes	Yes
4. Was the status of publication (i.e., grey literature) avoided as an inclusion criterion? The authors should state that they searched for reports regardless of their publication type. The authors should state whether or not they excluded any reports (from the systematic review), based on their publication status, language etc.	No	Yes	Yes	Yes
5. Was a list of studies (included and excluded) provided? A list of included and excluded studies should be provided	No	No	Yes	No
6. Were the characteristics of the included studies provided? In an aggregated form such as a table, data from the original studies should be provided on the participants, interventions and outcomes. The ranges of characteristics in all the studies analysed, e.g., age, race, sex, relevant socioeconomic data, disease status, duration, severity, or other diseases should be reported.	No	No	Yes	Yes
7. Was the scientific quality of the included studies assessed and documented? A priori methods of assessment should be provided (e.g., for effectiveness studies if author(s) chose to include only randomised, double‐blind, placebo‐controlled studies, or allocation concealment as inclusion criteria); for other types of studies alternative items will be relevant.	No	No	Yes	Yes
8. Was the scientific quality of the included studies used appropriately in formulating conclusions? The results of the methodological rigour and scientific quality should be considered in the analysis and the conclusions of the review, and explicitly stated in formulating recommendations.	No	No	Yes	Yes
9. Were the methods used to combine the findings of studies appropriate? For the pooled results, a test should be done to assess their homogeneity (i.e., *χ* ^2^ test for homogeneity, *I* ^2^).	Not applicable	Yes	Not applicable	Unclear
10. Was the likelihood of publication bias assessed? Assessment of publication bias should include a combination of graphical aids (e.g., funnel plot) and/or statistical tests (e.g., Egger regression test).	No	Yes	No	Yes
11. Was the conflict of interest stated? Potential sources of support should be clearly acknowledged in both the systematic review and the included studies.	No	No	Yes, for the review; No for included studies	No
Reviewers’ results/conclusions about effects of FFT	FFT is a ‘promising treatment,’ not ‘probably efficacious’ or ‘well established'.	Average ES for FFT vs. alternative treatments (*k* = 3) *d* = 0.29, 95% CI: −0.18 to 0.076, *p* = 0.19; ES for FFT vs. no treatment (*k* = 1) *d* = 0.82, 95% CI: 0.12 to 1.53, *p* < 0.05.	There is ‘insufficient firm evidence to allow any conclusion to be drawn on the effect of FFT for young people in treatment for non‐opioid drug use’ (2015, p. 5).	'FFT is more effective than no‐treatment or well‐defined [alternative treatments], but not more effective than TAU’ (2017, p. 607).

Abbreviations: AMSTAR, Assessment of Multiple SysTemAtic Reviews; CI, confidence interval; FFT, Functional Family Therapy; TAU, treatment as usual.

*Source*: AMSTAR was adapted from Shea et al. (2007).

Austin and colleagues (Austin, 2005) reviewed research on five family‐based treatments for adolescents with substance use problems. Only one FFT study was included. The reviewers classified FFT as a ‘promising treatment’ and noted that it did not meet the American Psychological Association (APA) criteria for ‘probably efficacious’ or ‘well established’ treatments.

Baldwin and colleagues (Baldwin, 2012) conducted a meta‐analysis of 24 studies comparing Brief Strategic Family Therapy, FFT, Multidimensional Family Therapy, or Multisystemic Therapy. They suggested that these four therapies had modest effects compared with treatment as usual (TAU) and alternative therapies. Three studies of FFT were included; one compared FFT to no treatment.

Filges and colleagues (Filges, 2015, 2018) conducted a systematic review of effects of FFT for non‐opiod drug use among youth. With only two included studies in the analysis, they found ‘insufficient firm evidence to allow any conclusion to be drawn on the effect of FFT for young people in treatment for non‐opioid drug use’ (2015, p. 5).

Hartnett and colleagues (Hartnett et al., 2017) conducted a review of 14 FFT studies and produced separate meta‐analyses for six subgroups of studies: results of RCTs and non‐randomised studies were synthesised separately, and (within each these two subgroups of studies) separate syntheses were conducted for studies that compared FFT to no treatment, TAU, or a ‘well‐defined’ alternative treatment. Authors aggregated outcome data for relevant between‐groups comparison within studies and reported six pooled estimates with two to five comparisons in each meta‐analysis. They used results of heterogeneity tests to determine whether to use fixed or random effects models (contrary to the advice of Borenstein, 2009, 2010). They concluded that ‘FFT is more effective than no‐treatment or well‐defined [alternative treatments], but not more effective than TAU’ (2017, p. 607).

Of the four reviews we analysed, the work of Filges and colleagues is most closely in line with the AMSTAR criteria. But this review was focused on a subpopulation of FFT cases. There is need for a full systematic review and meta‐analysis of FFT studies. Our review aimed to meet that need.

## 3 OBJECTIVES

We aimed to synthesise available data from eligible studies to assess the effectiveness of FFT for families of youth with behaviour problems.

## 4 METHODS

### 4.1 Criteria for considering studies for this review

#### 4.1.1 Types of studies

The protocol for this study was published in 2007 (Littell, 2007).

Two types of studies were eligible for this review: (1) randomised controlled trials (RCTs) and (2) quasi‐experimental designs (QEDs) that used parallel cohorts (i.e., multiple groups assessed at the same points in time) and statistical controls for between‐group differences at baseline.

#### 4.1.2 Types of participants

Participants were families of young people aged 11–18 with behaviour problems, such as: criminal offenses, delinquency, violent behaviour, anti‐social behaviour, and substance abuse.

#### 4.1.3 Types of interventions

Certified FFT programmes (as described above) compared with TAU, or alternative services (such as individual, family, or group therapy), or no treatment.

We included speciality FFT programmes developed for youth at risk of gang involvement (FFT‐G), because participants in these programmes meet our inclusion criteria (above). We did not include speciality FFT programmes for child welfare cases (FFT‐CAN and FFT‐TCM), as they involved younger children and those who do not necessarily have behavioural problems.

#### 4.1.4 Types of outcome measures

We examined outcomes related to youth behaviour and youth, parent, and family functioning. We included outcomes measured at any point in time after treatment. The measurement or reporting of specific outcomes (e.g., primary outcomes) was not used to determine whether studies were eligible for this review.

##### Primary outcomes

Primary outcomes refer to the young person who was the focus of intervention. These outcomes are:

- Recidivism (re‐referral for a behavioural offence, arrest or conviction for a criminal offence);
- Placement in a restrictive or secure facility (incarceration, detention, residential treatment, psychiatric hospitalisation);
- Internalising behaviour problems;
- Externalising behaviour problems;
- Self‐reported delinquency (SRD); and
- Drug and alcohol use.

##### Secondary outcomes

- Peer relations,
- Pro‐social behaviour,
- Self‐esteem,
- Parent symptoms and behaviour,
- Family functioning,
- School attendance, and
- School performance.

Acceptable outcome measures were derived from standardised instruments (such as the Child Behaviour Checklist, Achenbach, 1991); administrative data (police, court, and school records); biologic drug tests; and parent reports, teacher reports, self‐reports (e.g., regarding delinquency, drug‐use, peer relations) that had some empirical evidence of reliability or validity (e.g., Chronbach's *α* or *κ* > 0.7) in the study sample or a similar sample. We only used outcome data if valid measures were available for at least 50% of the cases in each comparison group.

We did not collect or analyse data on outcomes for siblings, nor did we analyse data on caregiver substance use.

### 4.2 Search methods for identification of studies

Initial searches were conducted in 2013–2014 and updated in August 2020.

#### 4.2.1 Electronic searches

Electronic searches were made of bibliographic databases as well as government policy databanks and professional websites. Reference lists of articles were examined, and experts contacted to search for so called ‘grey literature’. There were no publication, geographic, or language restrictions. Searches covered the following sources.

##### Bibliographic databases

APA PsycINFO (OVID) 1806 to August Week 4 2020 searched on 29 August 2020

ASSIA (Proquest) to 4 August 2020

Cambridge Core Collection on 30 August 2020

CINAHL (EbscoHost) 1937 to date on 03 March 2013; 1 January 2013 through 2020 on 29 August 2020

Cochrane Library (Wiley) all databases: 03 March 2013; 2013‐2020 on 30 August 2020

EMBASE Classic+Embase (OVID): 1947 to date on 28 August 2020

ERIC (OVID): 1965 to March 2020 on 29 August 2020

Norart: earliest to date on 29 August 2020

Ovid MEDLINE(R) ALL: 1946 to date on 28 August 2020

Science Direct: 1969 to date on 03 March 2013; 2013 to date on 30 August 2020

Sociological Abstracts (Proquest): earliest to date on 04 March 2013; 1 January 2013 to date on 31 August 2020

Social Care Online: earliest to date on 7 March 2013; 2007 to date on 30 August 2020

Social Work Abstracts: earliest to date on 04 March 2013; Social Services Abstracts (Proquest): 2013 to date on 29 August 2020

SveMed+ on 29 August 2020

WorldCAT dissertations and theses (OCLC) 2007 to date on 30 August 2020;

Dissertation Abstracts International (DAI) on 6 March 2013

Web of Science ISI earliest to date on 03 March 2013; Web of Science Core Collection earliest to date on 29 August 2020:

- Science Citation Index Expanded 1900–present
- Social Sciences Citation Index 1900–present
- Arts & Humanities Citation Index (1975–present)
- Conference Proceedings Citation Index‐ Science (1990–present)
- Conference Proceedings Citation Index‐ Social Science & Humanities (1990–present)
- Emerging Sources Citation Index 2015–present

##### Government and professional websites

Annie E. Casey Foundation (25 March 2014, 30 September 2020)

Blueprints for Violence Prevention (25 March 2014)

California Evidence‐Based Clearinghouse (25 March 2014)

Current Controlled Trials (25 March 2014)

Functional Family Therapy (www.fftinc.com, www.functionalfamilytherapy.com, www.fft-sverige.se) (25 March 2014)

UK Home Office: 25 March 2014, 30 August 2020

U.S. Centers for Disease Control: 25 March 2014, 30 August 2020

U.S. Department of Health and Human Services: 25 March 2014, 30 August 2020

U.S. Government Printing Office: 25 March 2014, 30 August 2020

U.S. National Institutes of Health, RePORTER (formerly CRISP) database: 25 March 2014, 2013‐2020 on 30 August 2020

Our protocol indicated that we would search C2‐SPECTR and InfoTrac, but these databases were no longer available.

##### Search engines

Google Scholar: we examined the first 100 hits (sorted by relevance) for ‘functional family therapy’ on 07 March 2013; and the first 100 hits (limited to 2013‐2020 and sorted by relevance) on 30 August 2020.

##### Search terms

Search terms for MEDLINE (modified as necessary for other databases) were:

1. functional family therapy.tw.

Detailed search strategies for all databases and websites are provided in Appendix 1. Search terms and strings were fairly simple, because we were searching for data on a named (branded) intervention. These simple terms and strings were found to be sufficient to retrieve all known studies, and no other useful terms were identified during the search process.

All searches were conducted in English; in 2020, searches were expanded to include both English and Norwegian terms in the Norart database and English and Swedish search terms in the SveMed+ database.

#### 4.2.2 Searching other resources

##### Personal contacts

We contacted principal investigators and authors to identify unpublished reports and ongoing studies. These contacts included: Robert Barnoski, Gunnar Bjørnebekk, Sandra Heriot, Stephanie Lee, Terje Ogden, Tim Ozechowski, Susan Regas, Michael Robbins, Dagfinn Thøgersen, Allen Thurston, and Holly Barrett Waldron.

##### Cross‐referencing of bibliographies

References in reviews and primary studies were scanned to identify new leads. We pursued all potentially‐eligible primary studies and potentially‐relevant reviews identified in bibliographies. We used backward citation searches and did not conduct forward citation searches.

### 4.3 Data collection and analysis

#### 4.3.1 Selection of studies

Two reviewers (JL, MB, AW, JR, CW, TL) independently read each title and abstracts and used the screening tool shown in Appendix 2 to exclude irrelevant studies. Any citation deemed potentially relevant by any one reviewer was retrieved in full text and moved to the next stage.

Before study eligibility decisions were made, we grouped retrieved reports into distinct studies. Each study contains a unique sample that does not overlap with another study sample. Many studies produced multiple reports on the same sample and/or on subsamples. Each study was identified by its first report (first author's surname and year), with multiple reports filed under each study name.

Two reviewers (JL, MB, AW, JR, CW, TL) independently read all available reports on retrieved studies to determine whether studies meet our eligibility criteria (described above and in Appendix 2). Disagreements were resolved by consensus or with a third author. Specific reasons for exclusion were documented for each study that did not meet our inclusion criteria.

#### 4.3.2 Data extraction and management

Information on study design and implementation, sample characteristics, intervention characteristics, and outcomes was extracted from studies and coded onto Excel spreadsheets, using data extraction and coding procedures adapted from Littell et al., 2021 (see Appendix 2). Two reviewers (JL, MB, AW, JR, CW, TL) independently coded each study. When inter‐rater differences occurred, these were discussed to refine coding schemes and resolve any discrepancies.

Data were organised in tables and figures. Data on effect sizes (ESs) were entered into RevMan for pair‐wise meta‐analysis.

#### 4.3.3 Assessment of risk of bias in included studies

Included studies were judged against the following criteria, which were adapted from the Cochrane Risk of Bias tool (version 1.0, Higgins & Green, 2011) and the What Works Clearinghouse standards for baseline equivalence (WWC baseline) and attrition (WWC attrition). Risk of bias ratings were not used as criteria for inclusion in the synthesis.

**Adequate sequence generation** (selection bias): investigators describe a random component in the sequence of assignments (e.g., computer‐generated random numbers, table of random numbers, drawing lots or envelopes, coin tossing, shuffling cards, or throwing dice).

- Yes = Low risk of bias
- Unclear: insufficient information (e.g., random assignment is mentioned, but not described in detail)
- No = High risk: investigators describe a non‐random component in the sequence of assignments (e.g., alternation or rotation, date of birth, date of admission or referral, case record number, clinical judgement, client preference, or service availability; non‐random addition, replacement, or removal of cases)

**Adequate allocation concealment** (selection bias): participants and investigators could not foresee assignment, because randomisation was performed at central site remote from the trial location or investigators monitored use of assignments contained in sequentially numbered, sealed, opaque envelopes.

- Yes = Low risk
- Unclear: insufficient information (e.g., random assignment is mentioned, but not described in detail) or adequacy of concealment is unclear (e.g., use of coin toss, card shuffle, dice, envelopes with unspecified characteristics)
- No = High risk: allocation was not adequately concealed; for example, investigators used open random number lists, transparent or unsealed envelopes, or quasi‐randomisation methods such as alternation or rotation, date of birth, date of admission or referral, case record number, or service availability.

**Baseline equivalence**: initial differences between groups were small or moderate (*d* ≤ 0.25) or researchers used statistical controls (e.g., PSM) for baseline differences.

- Yes = Low risk
- Unclear risk: insufficient information (e.g., group‐level on background characteristics were not provided, d cannot be computed, unclear if statistical controls were sufficient to create comparable groups)
- No = High risk: there were baseline differences between groups with *d* > 0.25, and no/inadequate statistical controls for these differences.

**Avoidance of performance bias** (confounding): no systematic differences between groups in levels of care or attention, or in exposure to factors other than the interventions of interest.

- Yes = Low risk
- Unclear (insufficient information)
- No = High risk: one group received more attention, care, or surveillance than another; or factors likely to be related to outcomes (confounding factors) were unequally distributed between groups.

**Avoidance of detection bias** (blinding): assessor is unaware of group assignment when collecting outcome data.

- Yes for all outcomes = Low risk
- Yes for some outcomes = Unclear
- Unclear (insufficient information)
- No = High risk

**Avoidance of attrition bias**: Losses to follow up were less than or equal to 25% and equally distributed (≤10% difference in response rates) across groups. Group equivalence on important baseline characteristics was retained after losses to follow‐up (*d* < 0.25).

- Yes for all outcomes = Low risk
- Yes for some outcomes = Unclear
- Unclear (insufficient information)
- No = High risk: loss of baseline equivalence (*d* > 0.25), losses to follow up > 25% overall, or losses were unequally distributed (>10% difference) across groups.

**Intention‐to‐treat** analysis: data were analysed according to participants’ initial group assignment, regardless of whether assigned services were received or completed.

- Yes for all outcomes = Low risk
- Yes for some outcomes = Unclear
- Unclear (insufficient information)
- No = High risk

**Standardised observation periods**: follow‐up data were collected from each case at a fixed point in time after random assignment, or analyses included controls for variable observation periods.

- Yes for all outcomes = Low risk
- Yes for some outcomes = Unclear
- Unclear (insufficient information)
- No = High risk

**Validated outcome measures**: use of instruments with demonstrated reliability (e.g., *α/κ* > 0.7) or validity in this sample or similar samples, or use of external administrative data on events (e.g., arrests, incarceration, hospitalisation).

- Yes for all outcomes = Low risk
- Yes for some outcomes = Unclear
- Unclear (insufficient information)
- No = High risk

**Free of selective reporting:** a prospective study protocol is available and all pre‐specified outcomes are reported in the pre‐specified way; all expected outcomes are reported in full and for all cases (e.g., no systematic exclusion of treatment non‐completers), regardless of the direction and statistical significance of results.

- Yes = Low risk
- Unclear (e.g., prospective protocol is not available, or changes in the protocol were made after the study began)
- No = High risk: some outcomes are not reported or are reported incompletely (e.g., non‐significant results are mentioned, but data are not provided; data are provided for selected subgroups only).

**Free of conflicts of interest (COI)** investigators state that they have no COI. Investigators would not benefit if results favoured FFT *or* control/comparison groups. None of the study authors, data collection staff, or data analysts were paid to develop, supervise, or provide services to the FFT or to the comparison group; none of these investigators are members of consulting firms linked to FFT (e.g., FFT Associates, FFT LLC, LIFFT) or comparison conditions.

- Yes = Low risk
- Unclear (insufficient information, no conflict of interest statement)
- No = High risk

##### Outcome‐level ROB assessments

We conducted separate assessments of risks of detection bias and attrition bias for two types of outcomes: (1) outcomes based on administrative data and (2) outcomes derived from interviews and self‐reports. This allowed us to capture the different risks of detection bias and attrition bias associated with data extracted from official agency records versus data collected in structured interviews with youth, caregivers, or others.

#### 4.3.4 Measures of treatment effect

Continuous data were analysed if means and standard deviations were available or there was some other way to calculate an ES (e.g., from *t*‐tests, *F*‐tests, or exact *p*‐values, see Lipsey, 2001). When reports provided insufficient data, we attempted to retrieve additional information from the study authors.

For continuous outcomes, standardised mean differences (SMD) were estimated using RevMan's formula for SMD, which is Hedges’ *g* (similar to Cohen's *d* with an adjustment for small sample bias).

For dichotomous outcomes, we calculated odds ratios and their 95% CIs. RevMan uses Mantel–Haenszel methods for combining binary outcome data across studies.

After computing ESs, we examined outliers and checked to make sure that our data accurately reflected study reports.

When reported results were clearly incorrect (e.g., a estimated effect was not included within its 95% CI), we did not include these data in our analyses. We queried authors about these results, and about very large outliers (see notes on Celinska, 2013).

#### 4.3.5 Unit of analysis issues

For cluster‐randomised trials and other studies with hierarchical data structures, we planned to ensure that standard errors were calculated correctly or make corrections so that ESs were properly weighted in meta‐analysis. We found no such studies.

Trials with multiple arms provided opportunities to examine multiple contrasts (e.g., between FFT and no treatment, FFT and individual cognitive/behavioural therapy [CBT] treatment, and FFT and group therapy). We extracted all relevant data, but took care to keep these estimates separate in pairwise meta‐analysis (data on one group could not be used twice in the same pairwise meta‐analysis; as explained below, all outcome data were included in correlated effect [CE] models).

#### 4.3.6 Dealing with missing data

We requested missing data from the primary authors of FFT studies.

We recorded data on attrition and differential attrition for each outcome and each endpoint. Where there was missing data for more than 50% of one or both comparison groups, we did not include that ES in meta‐analysis.

Where possible, we used Cochrane's revman‐calculator to calculate missing standard deviations.

#### 4.3.7 Assessment of heterogeneity

Heterogeneity was evaluated with the *χ* ^2^ test of heterogeneity and *I* ^2^.

#### 4.3.8 Assessment of reporting biases

Funnel plots were used to assess the risk of publication bias and other potential sources of bias. With fewer than 10 studies in the largest funnel plot, we were not able to use statistical tests for asymmetry.

We used all available reports on included studies (included registered protocols) to track reporting of outcomes within studies, across all endpoints and by outcome domain. Results of these analyses were arrayed graphically and used to support our judgements regarding risks of selective reporting.

#### 4.3.9 Data synthesis

We used pairwise meta‐analysis to synthesise data from multiple studies on comparable outcome measures at similar points in time. We also used CE meta‐analysis models to synthesise data on all available outcomes within conceptually distinct outcome domains. The methods we used for these two different kinds of meta‐analysis are explained below.

We did not expect all studies to produce estimates of the same population parameters, given the differences between them in characteristics of participants, interventions, and study designs. Thus, we used random effects models whenever possible (i.e., in pairwise meta‐analysis and in CE models with *df* > 4).

##### Pairwise meta‐analysis

We conducted separate pairwise meta‐analyses for each conceptually distinct outcome, within 10 outcome domains: recidivism (arrest or conviction), out‐of‐home placement, youth symptoms, delinquency, drug or alcohol use, peer relations, self esteem, parent functioning, family functioning, and school outcomes.

Contrasts between FFT and alternative treatments or TAU (estimates of relative effects) were kept separate from contrasts between FFT and no treatment (absolute effects) in pairwise meta‐analysis.

We used separate pairwise meta‐analyses to capture data gathered at different endpoints. We collapsed endpoints into the the following intervals: less than 9 months, 9–14 months, 15–23 months, and 24–42 months after treatment began. When a study had more than two data points within one of these intervals, we used the latest endpoint within that interval in pairwise meta‐analysis.

When studies reported multiple measures of recidivism at the same point in time, we selected the most comprehensive measure (e.g., any recidivism) over specific subtypes (e.g., misdemeaners, felonies, violent crimes) for pairwise meta‐analysis. When recidivism data were provided by multiple sources (interviews and official records), we used official records (administrative data) in pairwise meta‐analysis.

When studies provided multiple reports on parent or youth symptoms, we selected the most direct report (self‐report) for inclusion in pair‐wise meta‐analysis.

Pairwise meta‐analysis was conducted in RevMan Web. Inverse variance methods were used to pool SMDs, so that each ES was weighted by the inverse of its variance in an overall estimate of ES. Mantel–Haenszel methods were used to combine dichotomous outcome data (odds ratios) across studies. CIs of 95% were used for individual study data and for pooled estimates. Results are displayed in forest plots.

###### Correlated effects models

Intervention studies often report multiple dependent outcomes, including multiple measures of the same construct, reports on the same measure from multiple data sources, and repeated measures from the same sources over time. We used the CE model described by Pustejovsky, 2022 to handle these dependencies. The CE model is a method of robust variance estimation (RVE) with meta‐analysis. It assumes that ESs are correlated within studies, because they are derived from the same sample. This approach provides ‘valid point estimates, standard errors, and hypothesis tests even when the degree and structure of dependence between ESs is unknown’ (Fisher, 2015, p. 1; also see Hedges, 2010; Tanner‐Smith, 2014, 2016).

We use small sample corrections for RVE with meta‐analysis (Tipton, 2015).

Studies can report similar outcomes in different ways (e.g., days of drug use vs. days of abstinence from drug use) so, before performing the CE analysis, we reverse scored some outcomes so that positive scores always favour FFT.

We used all available data on our primary and secondary outcomes in the CE models, including multiple measures of the same outcome at different points in time. We assumed there was a correlation of 0.8 for ESs measured within the same study, but we tested this assumption with sensitivity analysis, assessing results for *ρ* = 0.0, 0.2, 0.4, 0.6, 0.8, and 1.0. Results showed that different values of *ρ* produced consistent estimates of mean ES coefficients, standard errors, and *τ* ^2^; all of these estimates were consistent within ±0.07.

We estimated ES models (both the mean ES model and any moderator models) using the R programmes *metafor* and *robumeta*.

The variance component for the random ES model was estimated in *robumeta* using restricted maximum likelihood (REML) estimation. When there were fewer than five studies reporting on an outcome in these analyses, we used a fixed effect (FE) model in *metafor* to compute the mean ES.

We computed separate CE estimates for dichotomous and continuous variables. For dichotomous outcomes, our synthesis was conducted using the log odds ratio (LOR), and we converted results back to odds ratios (ORs) for ease of interpretation. Then, to increase statistical power, we converted odds ratios to SMDs and produced CE models with all available outcomes in the analysis.

Results of CE models with fewer than four degrees of freedom are unreliable (Tanner‐Smith, 2014). In these instances, we examined forest plots of all ES, aggregated relevant ES within studies using the aggregate function in metafor. A correlation of 0.8 was assumed for associations among ESs within studies (we also tried this analysis under the assumption that the constant correlation among ES within studies of 0.6., and results didn't change much). We then used FE models to estimate the mean ES across studies.

Where possible, we provide 95% prediction intervals (PIs) as well as 95% CIs around point estimates of main effects. PIs (ES ± [1.96 × SQRT[*τ* ^2^]]) show the range of values within which results of future studies are likely to fall. Fixed effect models assume that *τ* ^2^ is zero and there are no PIs.

#### 4.3.10 Subgroup analysis and investigation of heterogeneity

Following our protocol, we assessed results of RCTs separately from results of QEDs, and we used moderator analysis to explore potential differences in ES estimates based on **study design** (RCTs vs. QEDs).

Previous meta‐analyses have shown that studies conducted by investigators who held an allegiance to the program they were studying produced significantly more positive results in favour of that program than investigators without such an allegiance (Luborsky, 1999; Shadish, 2002). We explored potential allegiance effects by comparing results of studies conducted by FFT program **developers** to those obtained by others.

In addition, moderator analysis was used to explore differences related to **location** (USA vs. other countries).

We had planned to use subgroup and moderator analyses to see if different results were obtained when FFT was compared to TAU versus alternative treatments (e.g., individual, group, or family therapies). But, as explained below, there was considerable diversity within these each of two **comparison conditions** and the differences between them (in terms of the duration, intensity, and amount of services families received) were negligible. Hence, we collapsed TAU and alternative treatments into a larger ‘active treatments’ group. We conducted separate analyses of the effects of FFT versus no treatment.

We had planned to identify distinct **subpopulations** of participants, so that we could assess effects of FFT for different kinds of youth. However, included studies had overlapping inclusion criteria. Regardless of whether their initial focus was on juvenile crime, delinquency, substance use, mental health, or other behaviour problems, these characteristics co‐occurred in all study samples. One study (Gottfredson, 2018) aimed to target youth ‘at risk’ of gang involvement, but eligibility for this study was based on the youth's own prior criminal involvement. Thus, we did not conduct analysis of effects of FFT for different subgroups of youth.

There were too few studies and not enough variation among studies to explore potential moderating effects of **risk of bias** variables such as baseline equivalence, attrition, and selective reporting.

#### 4.3.11 Sensitivity analysis

We used sensitivity analysis to examine the potentially biasing effects of outliers (e.g., studies with unusually large sample sizes, and those with extremely high or low ES). Sensitivity analyses were performed by removing studies one at a time from a forest plot or from CE analysis and comparing results with and without a study.

As mentioned above, we assessed the sensitivity of CE and FE models to various assumptions about the size of the correlations between ESs within studies.

##### Summary of findings and assessment of the certainty of the evidence

We used the GRADE guidelines (gdt.gradepro.org) to assess the certainty of evidence regarding primary outcomes in the Summary of findings Table. This (SoF) table includes measures of our six primary outcomes at approximately 1 year (6–12 months) after random assignment or referral.

## 5 RESULTS

### 5.1 Description of studies

Studies were identified using the search methods described above. Results of the search and characteristics of included and excluded studies are described below.

#### 5.1.1 Results of the search

**Figure 1:**
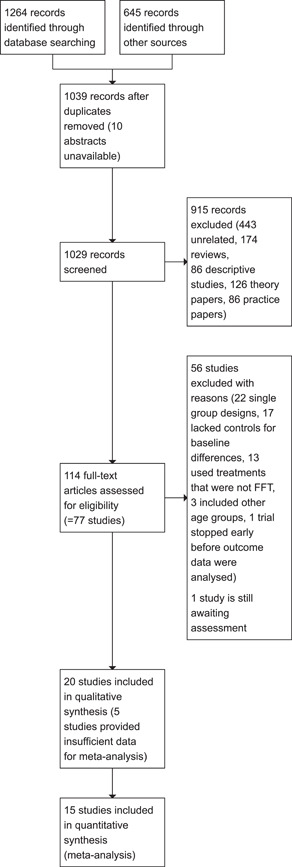
Study flow diagram.

As shown in Figure 1, electronic databases searches produced a total of 1264 citations: of these, 349 were found in Science Direct, 203 in APA PsycINFO, 174 in WorldCat dissertations and theses, 114 in Proquest databases (Sociological Abstracts and Social Work Abstracts), 107 in ISI Web of Science Core, 61 in the Cochrane Library, 60 in ASSIA, 60 in Embase, 52 in Medline, 50 in CINAHL, 19 in Social Care Online, 4 in SveMed+, 2 in Cambridge Core, and 2 in Norart.

Other sources produced another 645 citations: 200 from Google scholar, 300 from government and professional websites, 55 from personal contacts, and 90 references were harvested from other reviews.

After duplicate citations were removed, the searches yielded 1039 unique citation records.

We were able to screen all but 10 citations (abstracts and full text for these citations were not available). Of the 1029 titles and abstracts we screened, 915 were excluded from further examination (443 were unrelated to FFT, 174 were reviews of prior research; 86 were descriptive, correlational, or case studies; 126 were theory or position papers; and 86 were practice guidelines or manuals).

We retrieved full text reports for the remaining 114 citations. In addition, we retrieved 215 background papers and reviews, including 164 (94%) of the 174 reviews we identified.

As we studied these papers, questions arose about the nature of **focal interventions in some studies**. An early review of FFT studies included a ‘family therapy’ trial (Alexander, 1971) and indicated that FFT trials began in 1973 (Alexander, 1998; p. 55). Later reviews referred to the 1971 study as one of the FFT trials (e.g., Alexander, 2013; Robbins, 2016), although Sexton (2019) stated that FFT trials began in 1973. Little descriptive information is available about the nature of focal interventions in early trials and, given this lack of clarity, we excluded the 1971 trial.

Questions arose about whether and how some study **samples overlapped**. For example, three reports on an early FFT trial (Alexander, 1973) appear as three separate studies in several reviews (e.g., Alexander, 1998, 2013; Robbins, 2016), although Parsons and Alexander (1973) reported on the first 40 cases in this trial (according to Alexander & Parsons, 1973, p. 222) and Klein and colleagues (Klein, 1977) reported outcomes for younger siblings of youth in the same trial. These three reports list identical funding sources and grant numbers.

Similarly, Barnoski (2002) and Sexton (2010) appeared as separate studies in several reviews (e.g., Alexander, 2013; Robbins, 2016), even though the 2010 study ‘expanded upon’ the original sample.

Two reviews (Alexander, 2013; Robbins, 2016) described 27 studies based on 24 unique (non‐overlapping) samples.

Several citations described multiple studies and some studies had multiple citations. In all, we identified 77 unique studies with non‐overlapping samples (these studies were the subject of 129 unduplicated reports). We sought additional data on study methods from authors of several studies before making eligibility decisions.

Initial inter‐rater agreement on eligibility decisions was 90.6% with Cohen's *κ* = 0.77. Discrepancies were resolved through discussion.

Fifty‐six studies were excluded for specific reasons (described below), one study is still awaiting assessment, and no studies were classified as ongoing.

#### 5.1.2 Included studies

Twenty (20) studies met our inclusion criteria. These studies included a total of 12,129 families in FFT and comparison groups that were relevant for our review (not including groups that combined FFT with other treatments).

Whenever possible, we relied on multiple reports on the same study. When these reports provided conflicting evidence, we relied on the earliest report for information about study design, instrumentation, and implementation; and we relied on later reports for information on outcomes.

We sought additional information from authors of 10 included studies, and received unpublished data on three of these studies (Barnoski, 2002; Ogden, 2013; Robbins, 2012). Additional data were not available for one study (Regas, 1983), we are awaiting additional information from one study (Celinska, 2013), and authors of five completed studies did not respond to our requests for information (Eeren, 2018; Ozechowski, 2012; Waldron, Waldron 2001, Waldron 2005, Waldron 2008a).

Descriptive information on included studies is summarised in Table 2 (for more detailed information on these studies, see Characteristics of included studies). Table 2 describes all **20 included studies**, as well as the subset of **15 studies with valid data for meta‐analysis**. (For reasons explained below, Carr, 2014; Eeren, 2018, Ozechowski, 2012; Waldron, 2005, 2008a did not provide valid data for meta‐analysis.)

**Table 2:** Summary of characteristics of included studies.

Variable	Value	Included studies (*k* = 20)		Studies with valid data for meta‐analysis (*k* = 15)	
		*k*	%	*k*	%
Publication status	Unpublished reports only	7	35	5	33
	Published reports only	7	35	6	40
	Both	6	30	4	27
Year enrolment began	1970–1979	1	5	1	7
	1980–1989	1	5	1	7
	1990–1999	2	10	2	13
	2000–2009	8	40	7	47
	2010–2019	5	25	3	20
	Missing	3	15	1	7
Country	Ireland	1	5	0	
	Netherlands	1	5	0	
	Norway	1	5	1	7
	Sweden	1	5	1	7
	United Kingdom	1	5	1	7
	United States	15	75	12	80
Studies conducted with FFT developers	Yes	10	50	6	40
	No	10	50	9	60
Study type	Randomised	14	70	10	66
	Nonrandomised	6	30	5	33
Protocol available	Prospective	0		0	
	Retrospective	5	25	3	20
	None	15	75	12	80

The studies in our meta‐analysis included 10,980 families in relevant FFT and comparison groups.

The 15 studies included in meta‐analysis had a total of 42 reports (mean of 2.6 reports per study). Forty percent (6) of these studies had published reports only, 33% (5) had unpublished reports only and 27% (4) had both (see Table 2).

**Enrolment** in the first study occurred from 1970 to 1972; enrolment in the most recent study was completed in 2017. Four of the studies in our analysis sample began before the year 2000, seven began in the 2000s, three in the 2010s (with missing data on the timing of one study).

##### Study settings and investigators

Fifteen (75%) of the 20 included studies and 12 (80%) of the 15 studies with valid data were conducted in the USA. One study was conducted in each of the following **countries**: Ireland, the Netherlands, Norway, Sweden, and the UK.

Ten (50%) of all included studies were conducted with **developers** of the FFT program (4 of 10 developer‐involved studies did not provide valid outcome data). The analysis sample included six developer‐involved studies and nine independent studies.

We had planned to identify study settings in terms of the **service sectors** they operated in (e.g., juvenile justice, mental health, or child welfare), but several studies operated across service sectors. Some had multiple sources of referrals (e.g., Humayun, 2010) and others included youth involved in multiple service systems (e.g., Darnell, 2015).

##### Study methods

Of the 20 included studies, 14 were RCTs and six were QEDs that used statistical controls for baseline differences between groups. Four RCTs and one QED did not provide valid outcome data, leaving 10 RCTs and five QEDs for quantitative synthesis.

Some of the **RCTs** did not randomly assign all cases or used other decision rules to allocate cases to treatments. For example, random assignment in the Alexander (1973) study was affected by service ability and families’ religious affiliations (one comparison group was restricted to families of the Mormon faith). There was some lack of clarity about whether cases were randomly assigned to groups or randomly selected from a larger pool (e.g., in Alexander, 1973). We dealt with these issues when assessing risks of bias (discussed below).

Several **QEDs** attempted to match groups on demographic characteristics and pre‐treatment service or criminal history variables. Three studies (Baglivio, 2014, Darnell, 2015; Eeren, 2018) used propensity score matching (PSM) to create groups that were statistically equivalent on several key background variables, but that does not ensure equivalence on unmeasured characteristics. Other approaches to matching were not always successful in creating equivalent groups; for example, there were substantial between group differences on gender, race/ethnicity, and pre‐treatment criminal history in the Dunham (2009) and Celinska (2013) studies. Barnoski (2002) used statistical controls for baseline differences between FFT and TAU groups in age, gender, and risk factors, but reported that ‘youth viewed as most in need of services may have received preferential assignment to FFT rather than the control group, and the higher‐risk youth may have received preferential assignment to the better therapists’ (2004a, p. 7).

Several studies had **multiple arms**, allowing for multiple comparisons between FFT and other conditions. For example, Alexander (1973) included four comparison groups: FFT, group therapy, family therapy, and a no treatment control group. Regas (1983) included three groups (FFT, group therapy, and no treatment) for the first 2 months, then treatment was provided to the control group and the two active treatment comparison groups (only) were assessed at 7 months. Slesnick (2004) assigned youth to FFT or home‐based family therapy or TAU, providing two contrasts of interest. Darnell (2015) included four different treatment conditions, but only one contrast (FFT vs. probation TAU) was relevant for our purposes. The Robbins (2012) study used a factorial design with four arms, but only two of these arms (FFT vs. MET/CBT groups) were relevant for our review.

**Overlapping comparison groups**. In the Carr 2014 study, 82 cases were randomly assigned to FFT (*n* = 27) or TAU (*n* = 55). Cases remained in the TAU group until they were either lost to follow‐up (*n* = 11) or completed Time 2 (T2) assessments. Of the 44 cases that remained at T2, 14 did not meet study eligibility criteria; 15 of the remaining 30 cases were then ‘randomly assigned’ to FFT. Although this was a second (new) cohort of FFT cases, data on these 15 cases appear in aggregate reports of baseline, T2, and T3 data for *both* the FFT (reported *n* = 42) and TAU comparison groups (*n* = 55). Because the FFT and TAU groups overlapped (and cohorts were not truly parallel), data from this study could not be included in meta‐analysis.

None of the included studies had prospectively registered (or publicly available) protocols. Five included studies (3 in the analysis sample) had retrospectively registered protocols (registered after enrolment and data collection had begun).

##### Sample characteristics

Characteristics of our analysis sample (*k* = 15) are shown in Table 3 (for more information, see Characteristics of included studies). We were able to categorise these studies in terms of whether participants were identified as juvenile offenders (*k* = 7), substance abusers (*k* = 3), or youth with other behavioural and mental health problems (*k* = 5); however, most study samples include youth with multiple presenting problems and many of these youth were involved in multiple service sectors. For example, cases were referred to the Humayun (2010) study by juvenile justice, mental health, and community service programs. Another study (Darnell, 2015) included youth who were on probation following release from court‐ordered placements in foster care, group homes, and psychiatric hospitals, suggesting that youth were involved in juvenile justice, child welfare, and mental health services.

**Table 3:** Summary of characteristics of included studies with valid data for meta‐analysis.

Variable	Value	Included studies with valid data (*k* = 15)	
		*k*	%
Sample type: presenting problems	Juvenile offenders	7	47
	Substance abuse	3	20
	Other behaviour/MH problems	5	33
Service sector (referral source)	Juvenile justice	8	53
	Mental health	1	7
	Multiple sectors	6	40
Location type	Urban	3	20
	Rural	1	7
	Mixed	11	73
Sample size (number of cases assigned to relevant groups) Median = 116, IQR = 96 to 222, min = 20, max = 7618	<100	6	40
	101–200	4	27
	201–500	2	13
	501–1000	1	7
	1001+	2	13
Mean age of focal youth Mean = 14.2, SD = 0.8, min = 13.8, max = 17.1	<15	2	13
	15 to <16	9	60
	16+	2	13
	Missing	2	13
Gender of focal youth: % male	<50	2	13
	50–64	3	20
	65–79	7	47
	80–94	2	13
	95–100	1	7
Racial composition: % White	<35	4	27
	35–49	1	7
	50–64	2	13
	65–79	1	7
	80–94	2	13
	Missing	5	40
Racial composition: % Black	<35	6	33
	35–49	1	7
	80–94	1	7
	Missing	7	47
Ethnic composition: % Hispanic	<20	2	13
	20–50	4	27
	>50	2	13
	Missing	7	47
FFT: duration (mean number of days of service) Mean = 89, SD = 29, min = 28, max = 115	<60 days	2	13
	61–90 days	1	7
	90–115 days	6	40
	Missing	6	40
FFT: amount (mean number of hours of direct service) Mean = 9.4, SD = 2.2, min = 6.3, max = 13	<10	5	33
	10–13	2	13
	Missing	8	53
Comparison conditions Studies with 3 or 4 groups supported multiple comparisons; there were 20 relevant comparisons to FFT groups in 15 studies.	No treatment	2	
	Treatment as usual (TAU)	9	
	Other family treatment	3	
	Adolescent groups	4	
	Individual CBT	2	

Abbreviations: CBT, cognitive/behavioural therapy; FFT, Functional Family Therapy; IQR, interquartile range; MH, mental health.

One of the juvenile justice studies (Gottfredson, 2018) was described as youth at risk of gang involvement, although few of the study participants were ever involved in gangs.

Another study relaxed its initial eligibility to allow for inclusion of more low risk cases (Thørgersen, 2021 on the Ogden, 2013 study).

A few studies were located in either urban (*k* = 3) or rural (*k* = 1) areas, but most (11) covered a mixture of urban, suburban, and rural locations (Table 3).

###### Sample sizes

The number of cases in relevant comparison groups in studies in the analysis sample ranged from 20 to 7618 (median = 119, IQR = 96 to 222; as shown in Table 3).

###### Age, gender, and ethnicity

The average age of focal youth ranged from about 13.8 to 17.1 (mean = 14.2, SD = 0.8).

Most studies included a mixture of male and female youth, except the Gottfredson (2018) study, which was restricted to males. Only two studies (Alexander, 1973; Slesnick, 2004) included more girls than boys.

Ten studies provided some data on the racial/ethnic composition of their samples. Some were mostly White (Humayun, 2010), mostly Hispanic (Robbins, 2012), or mostly Hispanic and Black (Darnell, 2015, Turner, 2017).

##### Intervention characteristics

Some studies provided very little information on characteristics of FFT programs, and others described FFT programs that varied widely within and across studies.

FFT therapists focused on family systems, communication, and behaviour modification. Some used token economies and bibliotherapy in some cases (e.g., Alexander, 1973). FFT therapists often met with participants in offices, university laboratories, or clinic settings (Regas, 1983; Slesnick, 2004; Waldron, 2001).

As described in the Background section, FFT is organised in **phases**, which have evolved over time:

- two phases were described by Waldron (2001): (1) engagement and motivation and (2) behaviour change;
- a three phase approach was described by Dunham, 2009 and Celinska 2013: (1) engagement and motivation, (2) behaviour change, and (3) generalisation;
- and a five‐phase structure was used in four studies (Darnell, 2015, Humayun, 2010; Ogden, 2013; Robbins, 2012): (1) engagement, (2) motivation, (3) assessment, (4) behaviour change, and (5) generalisation.

FFT programs in the analysis sample ranged from 29 to 115 days weeks in **duration** (mean = 89 days, SD = 29, valid *k* = 9). Seven studies reported **amounts** of services received by FFT cases, with a range of 6–13 h of contact per family (mean = 9.4, SD = 2.2).

In the Regas (1983) study, for example, FFT consisted of eight weekly 1‐h family sessions. FFT therapists were trained by the researcher, who provided ‘live supervision,’ watching the sessions behind a one‐way mirror, redirecting sessions when goals were not met, and conferring with the therapist before the end of each session to formulate homework assignments (Regas, 1983, p. 67).

The **FFT‐G** program studied by Gottfredson 2018 included all of the features of FFT, along with efforts to address pressure from neighbourhood gang members and to engage families of gang‐involved youth (Gottfredson, 2018, p. 940). Development of the FFT‐G model was supported with $750,000 USD in grants from the US Office of Juvenile Justice and Delinquency Prevention in 2009–2010. FFT LLC staff produced the manual for FFT‐G and provided training and initial supervision for FFT‐G therapists in the Gottfredson 2018 study. Due to implementation problems, the FFT‐G program was not markedly different from other FFT programs.

Data on **service costs** were provided by Gottfredson 2018. The average cost of all services per family was $9888 USD per FFT‐G case and $9,031 USD per TAU case. This is because fewer TAU cases received any services. Some analyses of the cost data were restricted to comparisons between FFT‐G and the subset of TAU cases that received services; but the smaller denominator for TAU appears to inflate its real (total) cost.

##### Comparison conditions

FFT was compared with various forms of TAU, alternative treatments, and no treatment conditions. Some studies had three or four arms and supported multiple comparisons with FFT (see Table 3).

**No treatment comparison** conditions were included in two studies. In Regas (1983); the control group received FFT after 2 months and was not included in a subsequent follow‐up. Alexander (1973) compared FFT to a group that received no treatment in addition to groups that received alternative forms of treatment.

**TAU** was a comparison condition in nine studies, but the nature of TAU varied across studies. TAU included:

- usual probation services (Barnoski, 2002; Darnell, 2015);
- supervised probation (Hollimon, 2004);
- supervised probation and diversion services (Dunham, 2009);
- usual probation and an alternative form of family therapy (Gottfredson, 2018);
- any child welfare, mental health, or family counselling services (Ogden, 2013);
- casework services (Humayun, 2010); and
- services provided by runaway shelters (Slesnick, 2004).

Problems in implementation of TAU were reported in the Gottfredson 2018 study, where few TAU cases received the alternative family service and, as a result, a judge reassigned some of them to FFT.

**Alternative interventions** included:

- individual CBT for youth (Waldron, 2001),
- Individual counselling or mentoring for youth (Celinska, 2013),
- individual and family counselling (Hansson, 2000),
- group therapy for adolescents (Regas, 1983),
- client‐centred family groups (Alexander, 1973),
- eclectic‐psychodynamic family counselling (Alexander, 1973),
- ecologically‐based family therapy (Slesnick, 2004),
- Motivational Enhancement Therapy (MET)/CBT groups (Robbins, 2012), and
- Multisystemic Therapy (MST; Baglivio, 2014).

With relatively few studies in the review, we were unable to detect differences in the relative effects of FFT when compared with different types of active treatments. Given wide variations in the nature of services provided within the TAU and alternative intervention categories, and scant information on the duration and amounts of services provided under these rubrics, we collapsed these two types of comparison groups into a single ‘active treatment’ comparison category. When studies had more than one active comparison group (e.g., group therapy and family therapy), we combined data from both groups, using weighted averages and pooled standard deviations, to create a single active comparison group.

Only six studies reported data on the **amount of services** provided to cases in active comparison groups; study‐level averages ranged from 2 to 13 h per case (mean = 8.5, SD = 3.8, *k* = 6). The mean **duration** of these services ranged from 28 to 152 days (mean = 87, SD = 44, *k* = 7).

The Humayun (2010) study used a ‘dose control group’ approach in attempt to address the confounding factors of treatment, time, and attention. Because experimental treatments often provide more time and attention (a larger dose) than TAU conditions, and TAU services are generally available to all participants, Humayun and colleagues tried to balance the time and attention received by both groups by offering families in the control group an additional 12 h of regular casework service. They expected both groups to receive TAU plus 12 h of either FFT or 12 h of regular casework. But FFT cases actually received more TAU services than the control group (mean of 18 vs. 11 h) and more services overall (28 vs. 11 h).

Three studies reported similar amounts and similar duration of service for FFT and comparison cases (Regas, 1983; Robbins, 2012; Waldron, 2001).

##### Outcome measures

A glossary of abbreviations for outcome measures is provided in Appendix 3.

**Recidivism** was defined in several ways, including arrest or conviction for a criminal offence, and re‐referral for a behavioural offense (running away, habitual truancy, shoplifting, or possession of alcohol, soft drugs, or tobacco). Measures of recidivism were often based on official police and court records (administrative data). Some studies used youth and parent reports on recidivism as well.

Several studies identified out of home **placements** using administrative data from juvenile and adult courts. Darnell (2015) used these sources plus child welfare administrative data. Some studies relied on parent reports on young people's living arrangements.

Most assessments of symptoms, attitudes, behaviour, and **youth, parent, and family functioning** were obtained on standardised measures (e.g., the CBCL) embedded in interviews with youth and parents or primary caregivers. Sometimes reports came from teachers, probation officers, and clinicians.

Measures of **delinquency** were obtained with youth self‐reports on the SRD or other structured measures. Some studies also obtained reports on youth delinquent behaviour and from parents.

Data on **drug and alcohol use** were obtained from parent and youth reports. Some studies also used biologic (urine and saliva) tests.

Data on **school attendance** were gathered from youth and parent reports and (occasionally) from school records.

###### Timing of outcome measures

**Figure 2:**
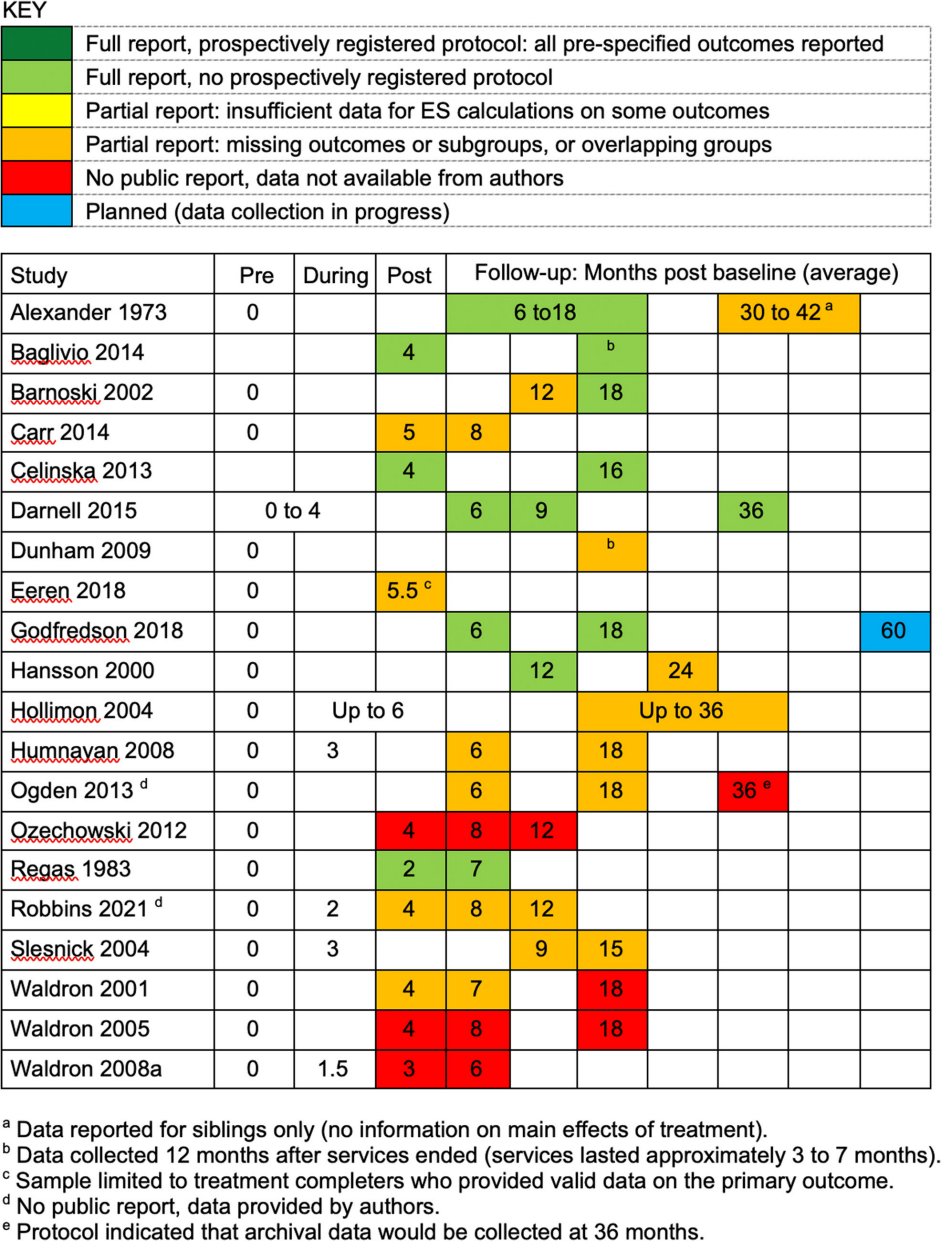
Timing and reporting of outcomes (*k* = 20 included studies).

Figure 2 shows the timing of assessments made in 20 included studies, as well as the status of reporting on outcomes at each endpoint. Most studies used multiple post‐treatment and follow‐up measures that ranged from 2 to 42 months after treatment began.

In some studies, there were wide variations in the timing of data collection within endpoints. For example, the first follow‐up occurred anywhere between 6 and 18 months, and a second follow‐up occurred between 2.5 and 3.5 years in Alexander (1973); Hollimon (2004) took up to 3 years for post‐treatment assessments.

A few studies (e.g., Baglivio, 2014) linked the timing of data collection to the end of treatment. In the Dunham (2009) study, FFT lasted 12–19 weeks and TAU (probation services) lasted 3–7 months; outcome data were collected 12 months after treatment completion, which created observation periods of different lengths.

As discussed earlier, Ozechowski (2012); Waldron (2005, 2008a) did not provide any outcome data, and results of the Carr 2014 trial could not be used in meta‐analysis because the treatment and control groups overlapped. The Eeren 2018 study did not provide data that could be used to estimate SMDs or ORs.

##### Confounded study characteristics

Several study characteristics appeared to be confounded. As shown in Table 4, 80% of FFT developer‐involved studies were RCTs, compared with 60% of independent studies (*d* = 0.54); but this difference was not present in our analysis sample (as four developer‐involved RCTs and one independent QED did not provide valid data).

**Table 4:** Confounded study characteristics.

	All included studies (*k* = 20)			Included studies with valid data for analysis (*k* = 15)		
	RCT		QED	RCT		QED
	*k*	Row %	*k*	*k*	Row %	*k*
FFT developer	8	80	2	4	67	2
Independent	6	60	4	6	60	3

	**USA**		**Other**	**USA**		**Other**
	***k***	**%**	***k***	***k***	**%**	***k***
FFT developer	9	90	1	6	100	0
Independent	6	60	4	6	60	3

Abbreviations: FFT, Functional Family Therapy; QED, quasi‐experimental design; RCT, randomised controlled trial.

Most (90%) of developer‐involved studies were conducted in the USA, compared with 60% of independent studies (*d* = 0.99). This confound was also observed in the analysis sample, where all developer‐led studies were conducted in the USA.

#### 5.1.3 Excluded studies

As shown in Figure 1, 56 studies were excluded from further consideration for the following reasons:

- 22 studies lacked sufficient comparison or control groups,
- 17 non‐randomised studies did not use statistical controls for baseline differences,
- 13 studies concerned programs that were not certified FFT programs (these included adaptations of FFT and combinations/integrations of FFT with other interventions),
- 3 studies included participants who did not meet our age criterion, and
- one trial (Thurston, 2015) was stopped before outcome data were analysed.

For more information, see Characteristics of excluded studies.

Another study (Lantz, 1982) is still awaiting assessment. This study was conducted 40 years ago, and we could not find any documentation of it.

### 5.2 Risk of bias in included studies

**Figure 3:**
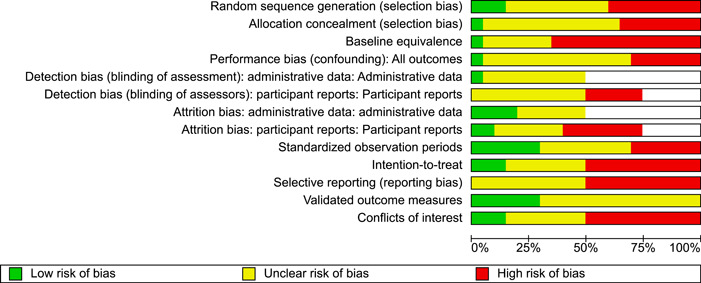
Risk of bias graph.

**Figure 4:**
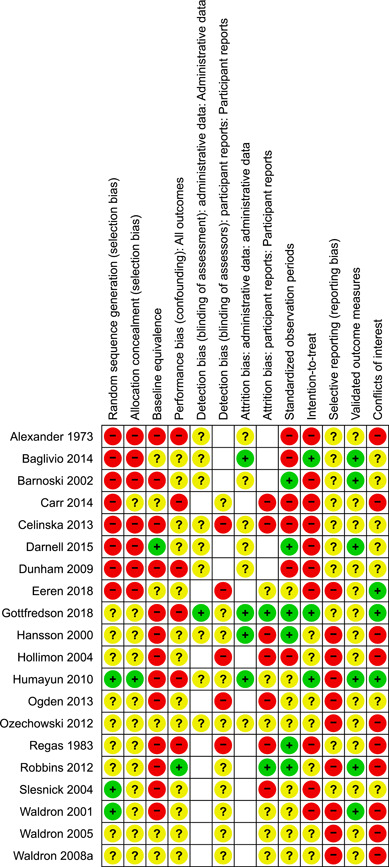
Risk of bias summary.

Our assessments of risks of bias used the definitions and criteria shown above and in Appendix 2. Results of these assessments are shown in Figures 3 and 4, and discussed below. Support for ROB assessments is provided in Characteristics of included studies.

As shown in Figure 3, more than half of included studies (*n* = 20) had high risks of bias on baseline equivalence, support for intention‐to‐treat analysis, selective reporting, and COI.

#### 5.2.1 Allocation (selection bias)

**Sequence generation**. Most quasi‐experimental studies were rated as having high risk of selection bias due to sequence generation. Some RCTs altered their sequence generation procedures to fit service availability (Alexander, 1973) or randomly assigned some cases twice (Carr, 2014).

**Allocation concealment** was not possible in most QEDS and was rarely discussed in RCTs.

**Baseline equivalence**. Although most studies used stratification, matching, or computerised urn randomisation in attempt to equalise baseline characteristics of groups, these efforts were not always successful given the relatively small sizes of some samples. There were substantial (*d* > 0.25) between‐group differences at baseline in 13 studies, and all of these studies were included in our meta‐analyses (see Figure 4).

Conflicting reports on baseline characteristics were provided on one study (Waldron, 2001; see Characteristics of included studies).

Baglivio 2014 reported group equivalence (*d* < 0.25) on all matching variables, including group averages on a three‐point regional score; but, equivalence on this mean scores does not insure that the groups were truly balanced across three regions.

#### 5.2.2 Blinding (performance bias and detection bias)

**Performance bias** (confounding) was a high risk in six studies. The trial by Humayun (2010) intended to guard against performance bias by providing comparable amounts of treatment (time and attention) to both groups; however, it was unable to do this (FFT cases received more TAU services than TAU cases).

Baglivio (2014) argued that differences in the length of service do not necessarily represent differences in the amount of service received, as service length can be influenced by case severity/complexity or difficulty of engagement.

**Detection bias** (blinding) was assessed separately for outcomes derived from administrative data and those obtained from interviews with program participants and others (see Figures 3 and 4). Assessments were not blind when conducted by program therapists, as in the Celinska (2013) study.

#### 5.2.3 Incomplete outcome data (attrition bias)

**Attrition** was assessed separately for outcomes derived from administrative data and those obtained from interviews with program participants and others. We assessed overall attrition and differential attrition. Ogden (2013) had more than 30% of cases with missing data on parent or youth reports at 6 and 18 months, and more than half were missing data from teachers’ reports at these end points. Humayun (2010) had more attrition on observation measures than self‐report instruments.

**Intention‐to‐treat (ITT) analysis** was reported in several studies, using very different definitions of this term. For example, Slesnick (2004) limited their ‘ITT’ outcome analyses to the subgroup of cases that had completed all four assessments (*n* = 75/119). Other studies systematically excluded cases that did not complete treatment, along with those with missing data (Celinska, 2013, Eeren, 2018).

#### 5.2.4 Selective reporting (reporting bias)

**Figure 5:**
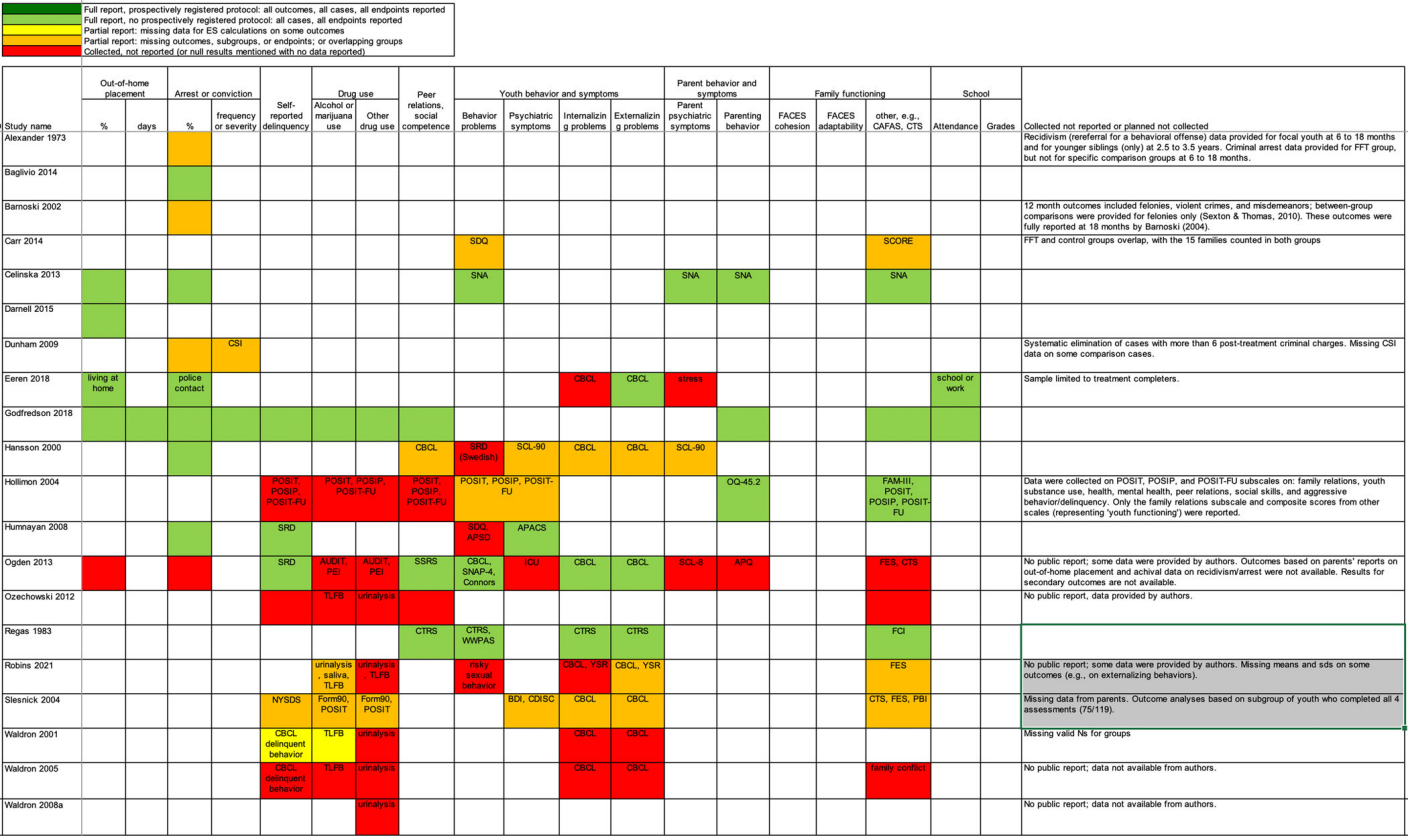
Full reporting, partial reporting, and non‐reporting of outcomes by study and outcome domain (*k* = 20 included studies).

Half (10) of the included studies had high risk of selective reporting, including six studies in our analysis sample. More detailed assessments of outcome reporting are shown in Figures 2 and 5; support for these assessments is provided in Characteristics of included studies.

None of the studies had prospectively registered protocols. Three studies in the analysis sample had retrospectively registered protocols (Humayun, 2010; Ogden, 2013; Robbins, 2012), as did two studies without usable data (Ozechowski, 2012; Waldron, 2008a).

When retrospective protocols were available, we compared the protocol and final reports. Some studies collected data on many secondary outcomes, but their reporting focused on primary outcomes (e.g., Humayun, 2010; Ogden, 2013). We could only distinguish primary and secondary outcomes for studies with protocols.

In some studies with multiple outcome measures, data reduction led to less‐than‐full reporting. For example, Robbins (2012) collected multiple measures of youth substance use, behaviour problems, and family functioning at multiple points in time. Authors reported selected computed/composite variables, subscales, and endpoints. In several studies, non‐significant results were not fully reported (i.e., subgroup means and standard deviations were missing).

#### 5.2.5 Other potential sources of bias

**Standardised observation periods**. As mentioned earlier, some studies had wide variations in the timing of data collection for some or all endpoints. It was not always clear whether observation periods began at the beginning or end of services. When observations were linked to the end of services, and duration of service varied, there were variations in the length of observation periods. In some studies this led to longer or shorter periods of observation for FFT versus comparison conditions. Six studies had high risks of bias related to observation periods (Figure 4).

**Validated outcome measures** were used in many studies, but some studies also included measures with little or no information on reliability or validity.

**COI** were present when researchers were also involved in the development and implementation of FFT or a comparison condition. The clearest statement of competing COI was provided by Robbins, 2012. The clearest statement of no competing interests or potential COI was provided by Humayun, 2010. Some studies did not provide statements about COI; these studies were coded as unclear risk of COI, unless there was other evidence of competing interests. Dissertations directed by FFT developers (e.g., Dunham, 2009) were coded as having unclear risk of COI.

### 5.3 Effects of interventions

Results of pairwise meta‐analyses are displayed in forest plots and described below. As discussed earlier, for purposes of pairwise meta‐analysis, we collapsed endpoints into three categories that best fit the available data: 6–12 months, 15–18 months, and 24–42 months after assignment to treatment.

As described in the Methods section, we computed separate CE estimates for dichotomous and continuous outcome variables and then converted odds ratios to SMDs and produced CE models with all available outcomes in the analysis. Where CE models were unreliable (*df* < 4), we used FE models to estimate average ESs. We limited these analyses to estimates of the relative effects of FFT compared with active treatments.

#### 5.3.1 Recidivism

Recidivism is defined as any behavioural offense or arrest or conviction for a criminal offence. Results of comparisons between FFT and any active treatment at 6–12 months are shown in Analysis 1.1. Four RCTs and two QEDs provided data on the proportion of cases that had recidivism within this time frame. Results of RCTs were heterogeneous (*τ*² = 0.73; *χ* ^2^ = 12.54, *df* = 3; *p* = 0.006; *I* ^2^ = 76%). Pooled effects from RCTs showed less recidivism in the FFT groups, but results were not significantly different from no effect (pooled OR = 0.58, 95% CI: 0.22 to 1.52, *p* = 0.27). Two QEDs produced results that were more homogeneous, with OR = 1.14 (95% CI: 0.77 to 1.68, *p* = 0.51). Differences between these two subgroups (RCTs and QEDs) were not statistically significant *p* = 0.21) and their pooled effect was OR = 0.79 with a wide 95% CI (0.45 to 1.40) and significant heterogeneity. The forest plot suggests that FFT was not consistently superior to active comparisons in reducing recidivism.

**Analysis 1.1:**
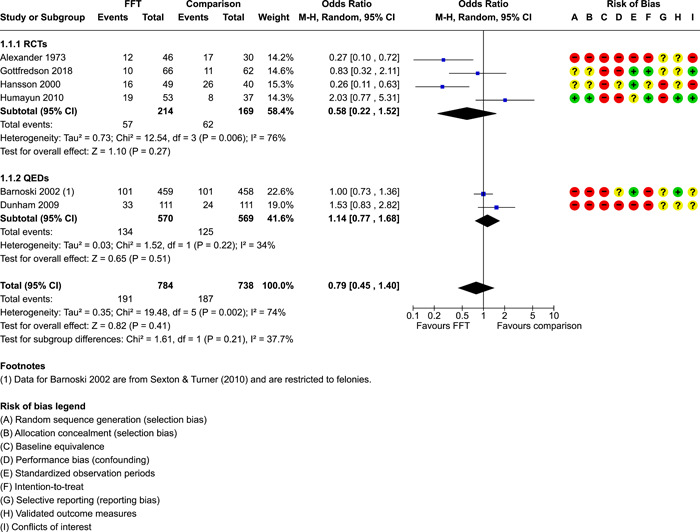
Comparison 1: Recidivism (arrest or conviction), Outcome 1: Recidivism, 6‐12 months

One RCT with high risks of bias provided data on effects of FFT on recidivism at 12 months, compared with a no treatment control group. Results, shown in Analysis 1.2, favoured FFT, but were not statistically different from zero.

**Analysis 1.2:**
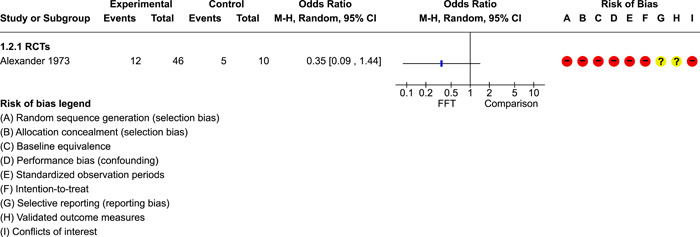
Comparison 1: Recidivism (arrest or conviction), Outcome 2: Recidivism, 12 months, FFT vs no treatment

Meta‐analysis showed that FFT had more consistent (homogeneous) effects across studies on recidivism at 15–18 months, both within and across subgroups of RCTs and QEDs (Analysis 1.3). The pooled ES was OR = 0.93 (95% CI: 0.78 to 1.11, *p* = 0.42).

**Analysis 1.3:**
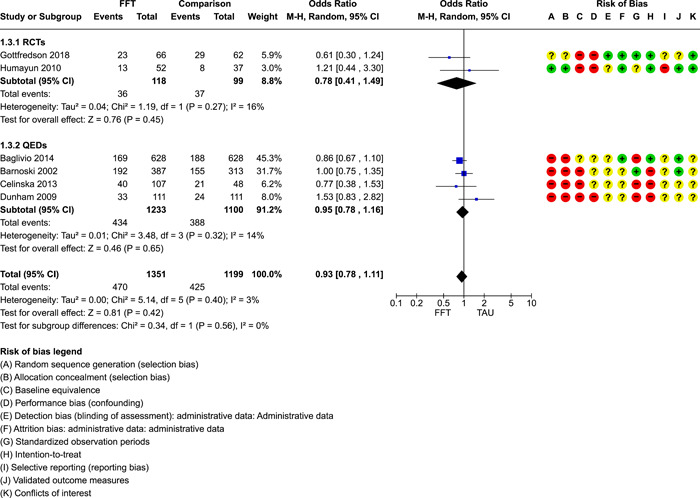
Comparison 1: Recidivism (arrest or conviction), Outcome 3: Recidivism, 15‐18 months

Two studies provided data on recidivism at 24 months (see Analysis 1.4). One small RCT showed that FFT dramatically reduced odds of recidivism; a larger QED indicated that FFT had no impact on this outcome. The pooled effect was OR = 0.41 with a wide CI (0.06 to 2.98) and substantial heterogeneity between studies.

**Analysis 1.4:**
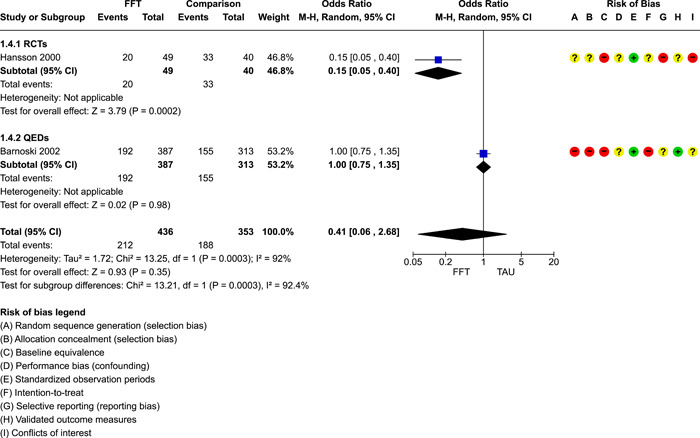
Comparison 1: Recidivism (arrest or conviction), Outcome 4: Recidivism, 24 months

The RCT by Gottfredson (2018) showed no real differences between FFT and comparison cases in terms of number of new arrests that had occurred by 6 and 18 months (Analysis 1.5, Analysis 1.6).

**Analysis 1.5:**
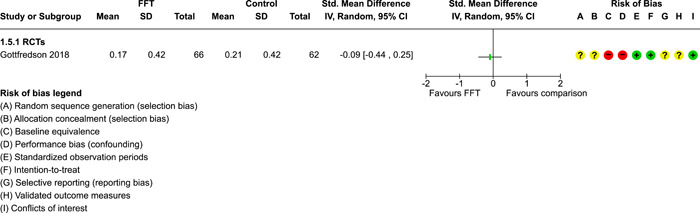
Comparison 1: Recidivism (arrest or conviction), Outcome 5: Number of arrests, 6 months

**Analysis 1.6:**
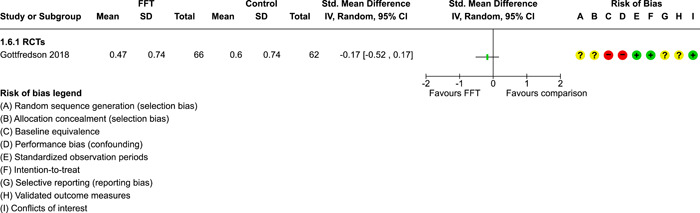
Comparison 1: Recidivism (arrest or conviction), Outcome 6: Number of arrests, 18 months

Results of CE models for all dichotomous measures of recidivism are shown in Table 5. Eight studies provided 42 dichotomous ES, with a pooled OR of 1.41 (95% CI: 0.77 to 2.58). Most of the variation in effects is between studies (*I* ^2^ = 73) and the estimated mean effect is not statistically different from no effect. Sensitivity analysis shows that the removal of one outlier leads to a smaller effect estimate and reduced heterogeneity. Two of these studies also provided four continuous measures of recidivism. When combined, all 46 ES yield a pooled ES estimate of 0.18 (SE: 0.14, 95% CI: −0.16 to 0.52; Table 5). This estimate indicates that FFT had a modest positive effect on recidivism across eight studies, but this effect is not significantly different from zero (no effect), and the PI suggests that future studies can be expected to produce estimates of effects of FFT on recidivism that range from −0.39 to 0.76 SMD.

**Table 5:** CE (and FE) estimates of effects of FFT by outcome domain.

Outcome domain and type (model)	Valid data		ES	SE	*t* or*Z*	*df*	95% CI		Sig	95% PI		Heterogeneity	
	*k*	nES					LB	UB		LB	UB		
Recidivism					*t*							***τ*** ^2^	*I* ^2^
Dichotomous	8	42	1.41	1.28	1.39	6.3	0.77	2.58		0.51	3.89		73.0
Dichotomous SA	8	41	1.36	1.27	1.32	6.1	0.77	2.42		0.54	3.47		69.9
Continuous	2	4	−0.11	0.22		1.0							
Combined	8	46	0.18	0.14	1.30	6.3	−0.16	0.52		−0.39	0.76	0.09	74.9
Placement					*Z*							*Q*	*p*
Dichotomous	3	11	1.45	1.17		1.9							
Continuous	2	7	0.04	0.23		1.0							
Combined	4	18	0.13	0.11		2.9							
Combined (FE)	4	18	0.14	0.12	1.13	3.0	−0.10	0.38				2.33	0.51
Youth behaviour					*t*							***τ*** ^2^	*I* ^2^
Dichotomous (FE)	1	4	0.50	1.29		3.0							
Continuous	7	70	−0.03	0.06	−0.45	4.6	−0.20	0.14		−0.22	0.16	0.01	13.8
Combined	7	74	−0.03	0.07	−0.44	4.6	−0.21	0.15		−0.24	0.18	0.01	16.4
Delinquency					*Z*							*Q*	*p*
Dichotomous	0	0											
Continuous	5	16	−0.04	0.09		3.7							
Continuous (FE)	5	16	−0.05	0.10	−0.46	4.0	−0.24	0.15				3.67	0.45
Substance use					*Z*							*Q*	*p*
Dichotomous	0	0											
Continuous	4	39	0.34	0.19		3.0							
Continuous (FE)	4	39	0.27	0.12	2.22	3.0	0.03	0.51	*			6.68	0.08
Peer relations					*Z*							*Q*	*p*
Dichotomous	0	0											
Continuous	3	15	0.03	0.20		1.6							
Continuous (FE)	3	15	−0.05	0.14	−0.40	2.0	−0.32	0.21				3.59	0.17
Self‐esteem	1	1											
Parent functioning					*Z*							*Q*	*p*
Dichotomous	0	0											
Continuous	5	22	−0.01	0.06		3.1							
Continuous (FE)	5	22	−0.01	0.10	−0.14	4.0	−0.21	0.18				1.13	0.89
Family functioning					*Z*							*Q*	p
Dichotomous	0	0											
Continuous	5	60	0.14	0.03		2.4							
Continuous (FE)	5	60	0.14	0.11	1.26	4.0	−0.08	0.35				0.75	0.94
School					*Z*							*Q*	*p*
Dichotomous	1	1											
Continuous	1	1											
Combined (FE)	1	2	−0.16	0.17	−0.92	1.0	−0.50	0.18				0.20	0.66
All domains					*t*							***τ*** ^2^	*I* ^2^
Dichotomous	9	59	1.39	1.26	1.43	7.2	0.81	2.41		0.49	3.95		72.6
Dichotomous SA	9	58	1.35	1.25	1.36	7.1	0.80	2.29		0.51	3.60		70.1
Continuous	10	234	0.07	0.11	0.67	8.1	−0.18	0.32		−0.50	0.65	0.09	62.6
Combined	15	293	0.19	0.09	2.01	12.5	−0.02	0.40		−0.37	0.75	0.08	68.2

*Note*: In preparation for this analysis, ES were adjusted so that positive ES always favour FFT. Results of CE models are shown unless FE model is noted. CE analyses assume 0.8 correlations among dependent ES within studies. Robust variance estimates were derived from CE models with small sample correlations. Results of CE models are not reliable if *df* < 4, so these reports are truncated. When CE models were not reliable, we aggregated relevant ES within studies (assuming intra‐study correlations of *ρ* = 0.8] and used the FE model to estimate the average ES across studies. *T* tests are used with CE models, *Z* tests with FE models. For combined analyses, ORs were converted to SMDs. ES = OR for dichotomous variables, SMD for continuous variables

Abbreviations: CE, correlated effect; CI, confidence interval; ES, effect size; FE, fixed effect; FFT, Functional Family Therapy; *K*, number of studies; LB, lower bound; nES, number of effect sizes; PI, prediction interval; SA, sensitivity analysis omitting one outlier from Celinska (2013); UB, upper bound.

Sig codes: **<0.01, *<0.05.

#### 5.3.2 Out‐of‐home placement

Gottfredson 2018 found no differences between FFT and comparison cases on odds of out‐of‐home placement at 6 months (Analysis 2.1).

**Analysis 2.1:**
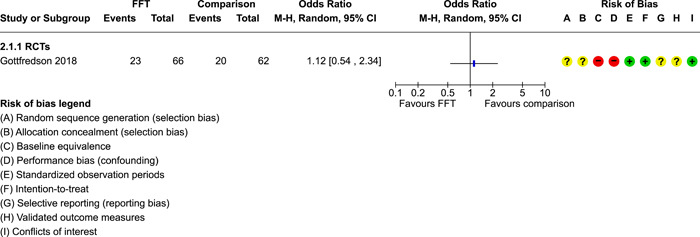
Comparison 2: Out of home placement, Outcome 1: Placement, 6 months

Two studies provided data on odds of out‐of‐home placements at 15–18 months, showing no real differences between groups within studies and no heterogeneity of results across studies (Analysis 2.2).

**Analysis 2.2:**
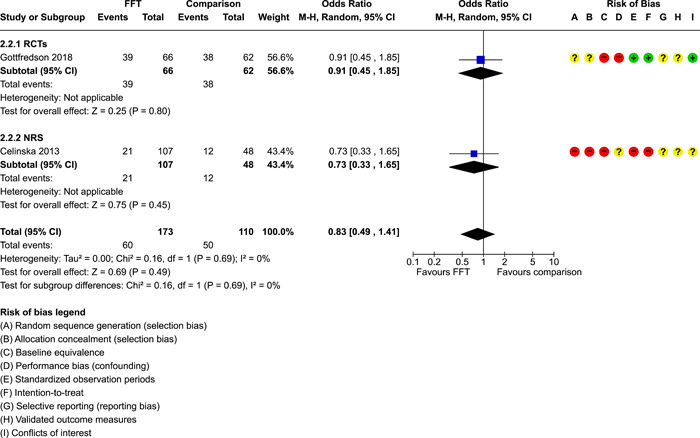
Comparison 2: Out of home placement, Outcome 2: Placement, 15‐18 months

Two RCTs found no differences in length of placements at 6–12 months (Analysis 2.3) or 15–18 months (Analysis 2.4). In the latter period, the Gottfredson 2018 study showed that FFT resulted in fewer placements (SMD = −0.31, 95% CI: −0.66 to 0.03) while the Slesnick (2004) study showed that FFT cases had more placements that comparison cases (SMD = 0.27, 95% CI: −0.20 to 0.75 (Analysis 2.4).

**Analysis 2.3:**
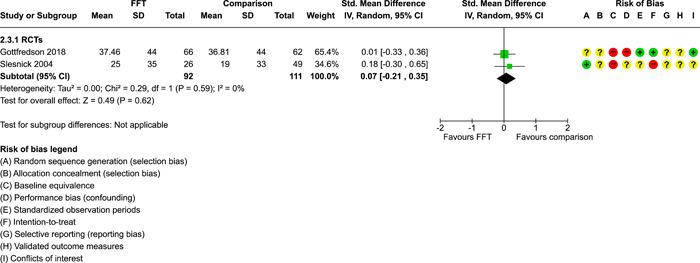
Comparison 2: Out of home placement, Outcome 3: Days in placement, 6‐12 months

**Analysis 2.4:**
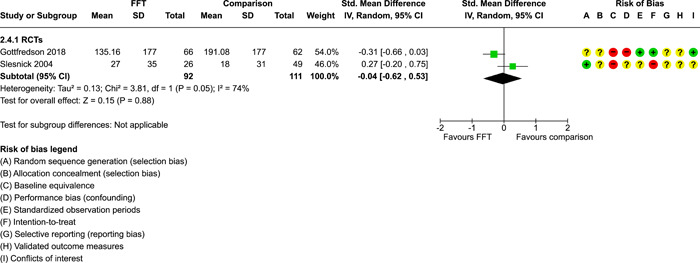
Comparison 2: Out of home placement, Outcome 4: Days in placement, 15‐18 months

Only four studies provided any data on placement outcomes. This was not sufficient for CE analysis (*df* < 4; see Table 5). As explained in the methods section, we aggregated ESs within each of these four studies, then used a FE model to obtain an estimate of effects across studies. The *Q* test for heterogeneity shows that results are consistent across studies (*Q* = 2.33, *p* = 0.51). The FE estimate indicates that FFT had a small positive effect on placement outcomes in these four studies (ES = 0.14, SE = 0.12, 95% CI: −0.10 to 0.38), but this effect is not significantly different from no effect.

#### 5.3.3 Youth behaviour problems

Two RCTs provided data showing no evidence of relative effects of FFT on externalising behaviours at 6–12 months (Analysis 3.1) or 15–18 months (Analysis 3.2). The same studies showed no evidence of effects on internalising behaviours 6–12 months (Analysis 3.3) or 15–18 months (Analysis 3.4). A third study found no significant differences on total CBCL scores at up to 36 months (Analysis 3.5).

**Analysis 3.1:**
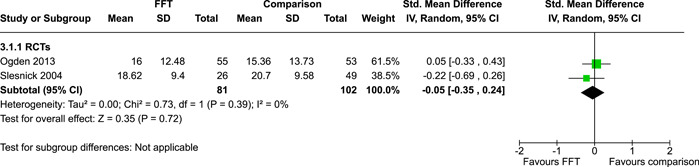
Comparison 3: Youth behaviour problems and symptoms, Outcome 1: Externalizing behaviour, 6‐12 months

**Analysis 3.2:**
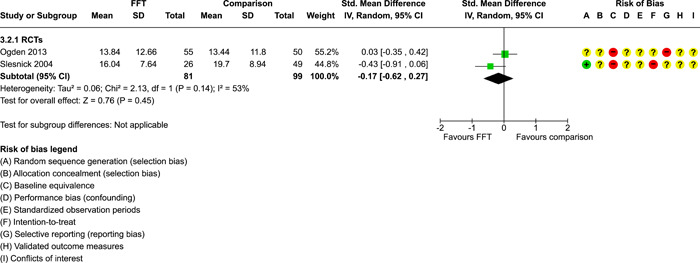
Comparison 3: Youth behaviour problems and symptoms, Outcome 2: Externalizing behaviour, 15‐18 months

**Analysis 3.3:**
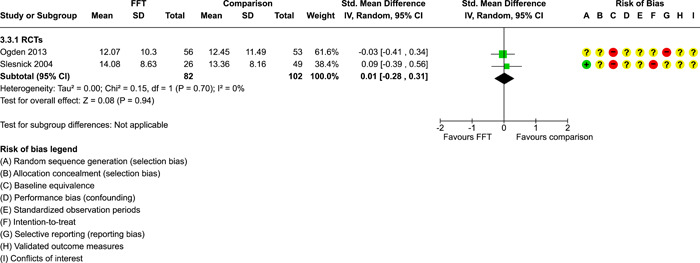
Comparison 3: Youth behaviour problems and symptoms, Outcome 3: Internalizing behaviour, 6‐12 months

**Analysis 3.4:**
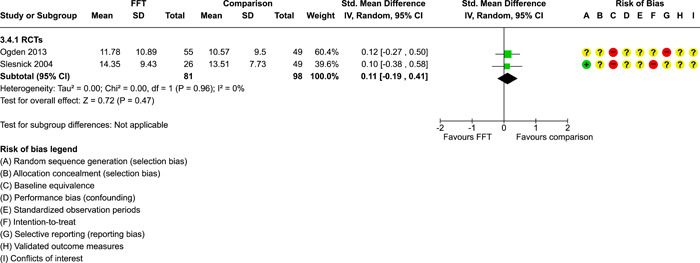
Comparison 3: Youth behaviour problems and symptoms, Outcome 4: Internalizing behaviour, 15‐18 months

**Analysis 3.5:**
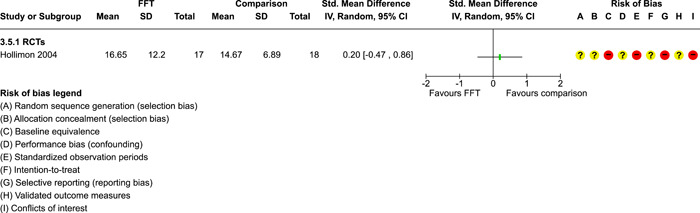
Comparison 3: Youth behaviour problems and symptoms, Outcome 5: CBCL total, up to 36 months

Seven studies provided a total of 74 ES on youth behaviour problems and symptoms (Table 5). The overall CE estimate suggests that FFT had a slight negative effect on these problems (ES = −0.03, SE = 0.07), but this effect was not significantly different from zero. There is little heterogeneity between studies in this analysis (*τ* ^2^ = 0.01, *I* ^2^ = 16.4). The PI suggests that future studies can be expected to show that FFT results in either negative or positive effects on youth behaviour and symptoms (ranging from −0.24 to 0.18 SMD).

#### 5.3.4 SRD

SRD scores were reported by five RCTs at 6–12 months (Analysis 4.1) and three RCTs at 15–18 months (Analysis 4.2). Study‐level and pooled ESs provided no evidence of effects of FFT on SRD. Results were somewhat homogeneous across studies.

**Analysis 4.1:**
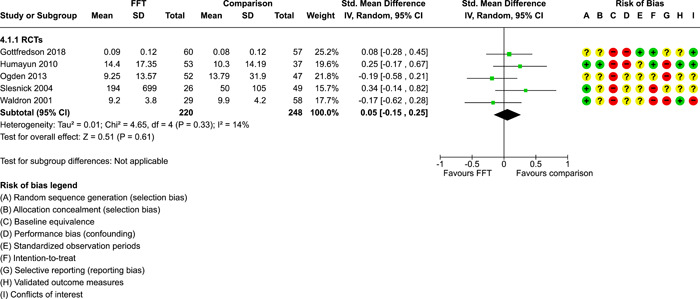
Comparison 4: Delinquency, Outcome 1: Delinquency scores, 6‐12 months

**Analysis 4.2:**
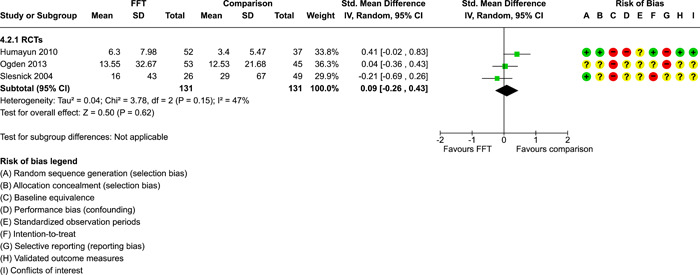
Comparison 4: Delinquency, Outcome 2: Delinquency scores, 15‐18 months

In all, only five studies provided data on delinquency outcomes. The FE estimate shows a slight negative effect of FFT on these outcomes (ES = −0.05, SE = 0.10), but this is not significantly different from no effect. Again, results are somewhat homogeneous (*Q* = 3.67, *p* = 0.45).

#### 5.3.5 Substance use

Marijuana use was the most often reported form of drug use. Three RCTs provided inconsistent evidence of effects of FFT on marijuana use at 6 to 12 months (Analysis 5.1). The pooled effect was SMD = 0.02 (95% CI: −0.23 to 0.26), not significantly different from no effect. The *χ* ^2^ test of heterogeneity was not significant (1.01, *p* = 0.60).

**Analysis 5.1:**
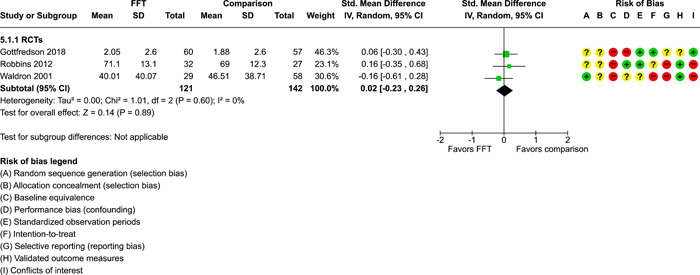
Comparison 5: Substance use, Outcome 1: Marijuana use, 6‐12 months

One study found no significant effects of FFT on drug or alcohol use (combined) at 9 months (Analysis 5.2) and 15 months (Analysis 5.3).

**Analysis 5.2:**
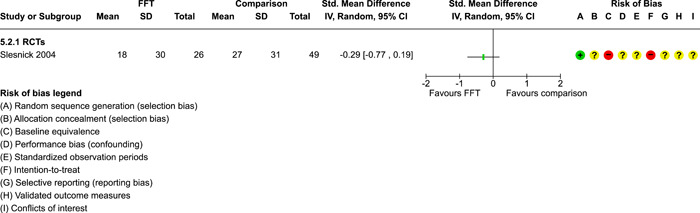
Comparison 5: Substance use, Outcome 2: Drug/alcohol use, 9 months

**Analysis 5.3:**
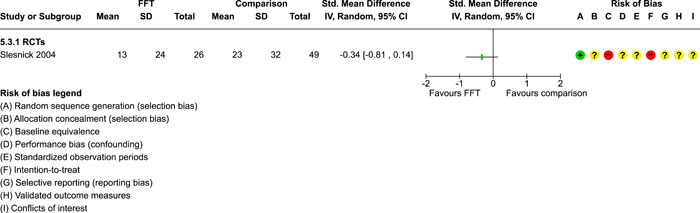
Comparison 5: Substance use, Outcome 3: Drug/alcohol use, 15 months

Overall, four studies provided data on 39 substance use outcomes. The FE estimate shows an overall positive effect across these studies (SMD = 0.27, SE = 0.12; Table 5) and this effect is statistically different from zero. Analysis of average effects within studies (Table 6) shows that FFT had significantly positive effects on substance use outcomes in two studies and null effects in two studies.

**Table 6:** Study‐level mean effects on substance abuse outcomes (FE estimates).

Study	nES	Mean SMD	SE	*z*	95% CI		
					LB	UB	Sig
Waldron (2001)	4	0.50	0.13	3.75	0.24	0.77	***
Slesnick (2004)	28	0.06	0.05	1.21	−0.04	0.17	
Gottfredson (2018)	5	0.27	0.08	0.32	−0.14	0.19	
Robbins 2021	2	0.84	0.21	3.97	0.42	1.25	***
Overall (k = 4)	39	0.27	0.12	2.22	0.03	0.51	*

Abbreviations: FE, fixed effect; nES, number of effect sizes; SMD, standardised mean difference. CI, confidence interval; LB, lower bound, UB, upper bound

Sig codes: *<0.05; **<0.01; ***<0.001.

We now turn to look at our secondary outcomes.

#### 5.3.6 Peer relations

One study provided data on peer relations, showing no evidence of effects at 6 or 18 months, although non‐significant results favoured the comparison group (Analysis 6.1, Analysis 6.2).

**Analysis 6.1:**
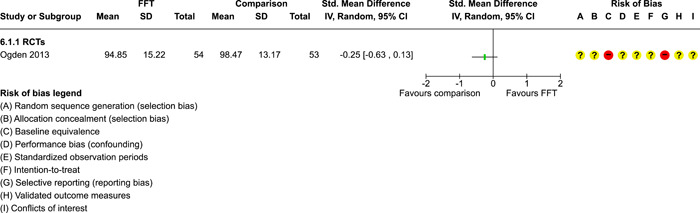
Comparison 6: Peer relations, Outcome 1: Social skills, 6 months

**Analysis 6.2:**
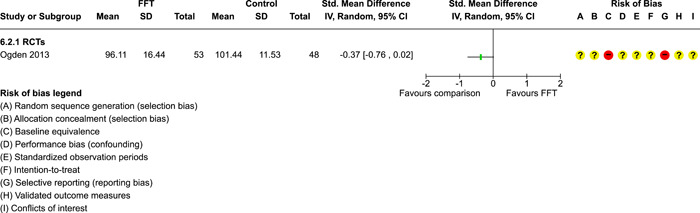
Comparison 6: Peer relations, Outcome 2: Social skills, 18 months

Another study found no differences in youth gang involvement at 6 months (Analysis 6.3).

**Analysis 6.3:**
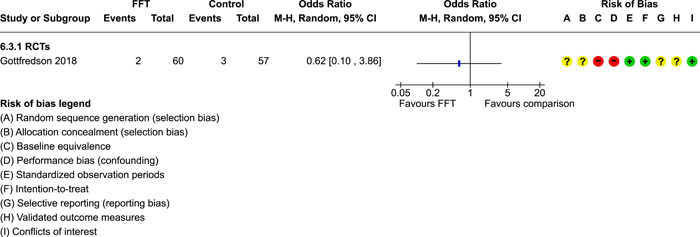
Comparison 6: Peer relations, Outcome 3: Gang involvement, 6 months

Overall, three studies provided data on 15 peer relations outcomes, including social ability, social skills, cooperation, assertiveness, responsibility, peer influence, and positive views of peers. The FE estimate of the mean effects of FFT on outcomes in this domain is negative and not significantly different from zero (ES = −0.05, SE = 0.14; Table 5). These results are not heterogeneous across studies (*Q* = 3.59, *p* = 0.17).

#### 5.3.7 Self‐esteem

One study showed non‐significant results favoring comparison cases on a measure of youth self‐esteem at 7 months (Analysis 7.1). There were no significant differences between FFT and no treatment at 2 months, although the FFT group performed slightly better than no‐treatment controls on this measure (Analysis 7.2).

**Analysis 7.1:**
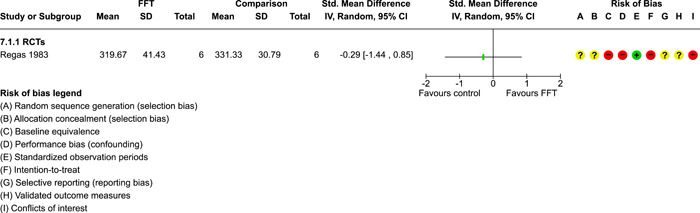
Comparison 7: Self esteem, Outcome 1: Youth self‐esteem, 7 months

**Analysis 7.2:**
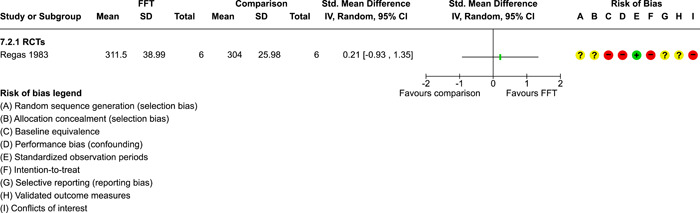
Comparison 7: Self esteem, Outcome 2: Youth self‐esteem, 2 months, FFT vs no treatment

#### 5.3.8 Parent functioning

Included studies used very different measures of parents’ symptoms and behaviours, tapping dissimilar constructs such as parents’ health, mental health, perceived social support, and parenting behaviours (e.g., monitoring, supervision, discipline, child care). This lack of comparability of measures made it difficult to synthesise results in pairwise meta‐analysis. One study showed no significant differences in parenting skills at 6 months (Analysis 8.1), another found no differences in parental care at 9 and 15 months (Analysis 8.2, Analysis 8.3), and a third study found no differences in parent functioning over 36 months (Analysis 8.4).

**Analysis 8.1:**
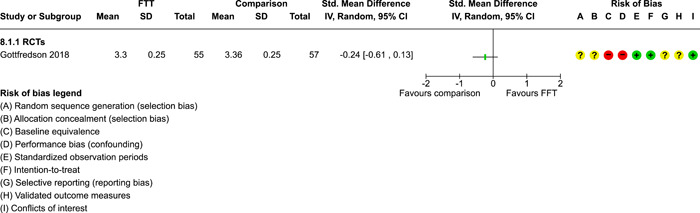
Comparison 8: Parent functioning, Outcome 1: Parenting skills, 6 months

**Analysis 8.2:**
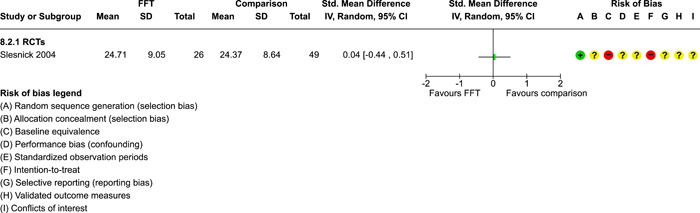
Comparison 8: Parent functioning, Outcome 2: Parental care, 9 months

**Analysis 8.3:**
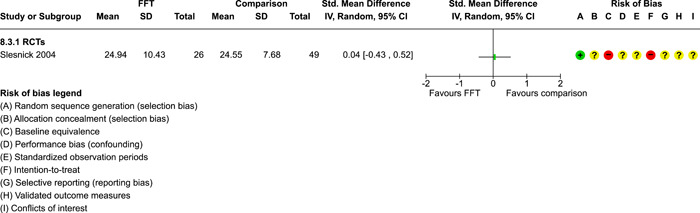
Comparison 8: Parent functioning, Outcome 3: Parental care, 15 months

**Analysis 8.4:**
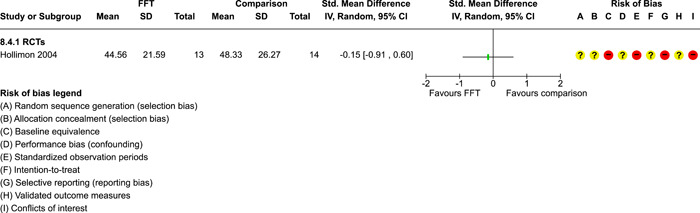
Comparison 8: Parent functioning, Outcome 4: Parent functioning, up to 36 months

Overall, five studies provided 22 estimates of effects of FFT on parents symptoms and behaviour. The FE estimate shows that the mean effect is close to zero (SMD = −0.01, SE = 0.10; Table 5). There is no significant heterogeneity in these outcomes across studies (*Q* = 1.13, *p* = 0.89).

#### 5.3.9 Family functioning

Two studies showed positive, non‐significant effects of FFT on overall family functioning at 6–7 months (pooled SMD = 0.19, 95% CI: −0.16 to 0.54; Analysis 9.1). Another study showed non‐significant reductions in family problems at up to 36 months (SMD = −0.21, 95% CI: −0.97 to 0.54; Analysis 9.2).

**Analysis 9.1:**
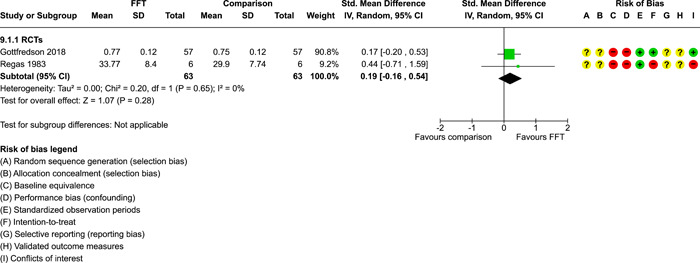
Comparison 9: Family functioning, Outcome 1: Family functioning, 6‐7 months

**Analysis 9.2:**
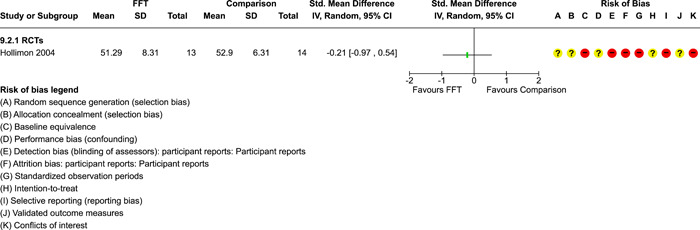
Comparison 9: Family functioning, Outcome 2: Family functioning, up to 36 months

Five studies provided a total of 60 measures of family functioning. The FE mean is positive (SMD = 0.14, SE = 0.11), homogeneous (*Q* = 0.76, *p* = 0.94), and not significantly different from zero (Table 5).

#### 5.3.10 School outcomes

One study found non‐significant differences favoring comparison cases in in the odds of school attendance (Analysis 10.1) and frequency of school attendance (Analysis 10.2) at 6 months.

**Analysis 10.1:**
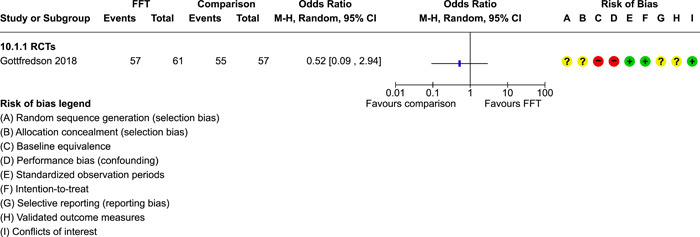
Comparison 10: School, Outcome 1: School attendance, any, 6 months

**Analysis 10.2:**
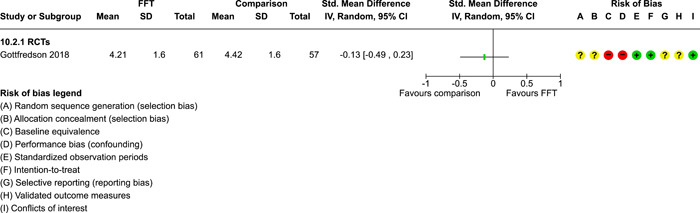
Comparison 10: School, Outcome 2: School attendance, days/week, 6 months

#### 5.3.11 All outcomes combined

Fifteen studies provided 293 ES estimates across all outcome domains (Table 5). Dichotomous measures suggest that FFT increased the odds of positive outcomes by about 40% (OR = 1.39, SE = 1.26, 95% CI: 0.81 to 2.41), but this effect is not significantly different from no effect. Most of the variation in these ESs was between studies (*I* ^2^ = 72.6). Future studies can be expected to produce dichotomous outcomes that range from reductions in the odds of positive effects by about half (PI LB = 0.49) to a fourfold increase in the odds of positive effects (PI UB = 3.95).

Across all continuous measures, the CE estimate suggests that FFT had small, positive effects that are not statistically different from zero (ES = 0.07, SE = 0.11; 95% CI: −0.18 to 0.32). Future studies can be expected to produce continuous outcomes that show that effects of FFT ranging from reductions in positive outcomes by 0.50 SD to improvements in outcomes by 0.65 SD (Table 5).

Across 15 studies and 293 ES measures, FFT has positive but non‐significant effects (SMD = 0.19, SE = 0.09). There is some heterogeneity of effects across studies, with *τ* ^2^ = 0.08 and *I* ^2^ = 68.2, indicating that more than two‐thirds of the variation in ESs lies between studies. Future studies can expect to find that FFT produces a wide range of results, from moderate negative effects to strong positive effects (PI: −0.37 to 0.75; Table 5).

#### 5.3.12 Subgroup and moderator analysis

We conducted separate moderator analyses to see whether there were systematic differences based on study design (RCTs compared with non‐RCTS), location (USA vs. other countries), and between studies conducted by FFT developers or independent investigators. There was no missing data on these moderators. Because county and investigator independence were confounded (there were no studies conducted by FFT developer outside of the USA; Table 4), we combined these two variables and examined differences among three groups of studies: independent studies conducted outside of the USA, independent studies within the USA, and developer‐led studies in the USA.

Results for RCTs versus non‐RCTs (QEDs) are shown in Table 7. On average, RCTs produced more positive ES estimates than non‐RCTs (a difference of 0.23 SMD, SE = 0.17), but this difference was not statistically significant. Although RCTs produced higher estimates of effects, within both subgroups of studies, effects of FFT were not statistically different from zero.

**Table 7:** CE estimates of effects of FFT on all outcomes, by study design.

	Valid data						95% CI			Heterogeneity	
	*k*	nES	ES	SE	*t*	*df*	LB	UB	Sig	***τ*** ^2^	*I* ^2^
Moderator analysis	15	293								0.09	67.8
Intercept			0.06	0.10	0.57	3.7	−0.23	0.34			
RCT			0.23	0.17	1.35	9.0	−0.16	0.63			
Subgroup means											
QED	5		0.06	0.10	0.57	3.7	−0.23	0.34			
RCT	10		0.29	0.14	2.06	8.4	−0.03	0.62			

Abbreviations: CI, confidence interval; FFT, Functional Family Therapy; LB, lower bound; nES, number of effect sizes; QED, quasi‐experimental design; RCT, randomised controlled trial; UB, upper bound.

*<0.05, **<0.01, ***<0.001.

As shown in Table 8, the highest mean ESs were produced by studies conducted in the USA by FFT developers (mean ES = 0.29, SE = 0.22), followed by USA independent studies (mean ES = 0.17, SE = 0.05), and non‐USA independent studies (mean ES = 0.15, SE = 0.34). Overall differences between these subgroups were not statistically significant. Pooled effects of FFT were not significant in the subgroup six studies conducted in the USA by FFT program developers, or for three studies conducted outside of the USA by independent investigators (the latter estimate is not reliable, as *df* < 4); results of independent studies in the USA favoured FFT.

**Table 8:** CE estimates of effects of FFT on all outcomes, by country and investigator independence.

	Valid data						95% CI			Heterogeneity	
	*k*	nES	Est	SE	*t*	*df*	LB	UB	Sig	***τ*** ^2^	*I* ^2^
Moderator analysis	15	293								0.11	71.8
Intercept			0.15	0.34	0.42	1.99	−1.35	1.64			
USA independent			0.02	0.35	0.06	3.98	−0.95	0.99			
USA developers			0.14	0.41	0.34	4.52	−0.94	1.22			
Subgroup means											
Non‐USA, independent	3		0.15	0.34	0.42	2.0	−1.35	1.64			
USA, independent	6		0.17	0.05	3.15	5.4	0.03	0.30	*		
USA, FFT developers	6		0.29	0.22	1.31	4.4	−0.30	0.87			

Abbreviations: CI, confidence interval; FFT, Functional Family Therapy; LB, lower bound;nES, number of effect sizes; UB, upper bound.

*<0.05, **<0.01, ***<0.001.

#### 5.3.13 Assessment of publication bias and small study effects

**Figure 6:**
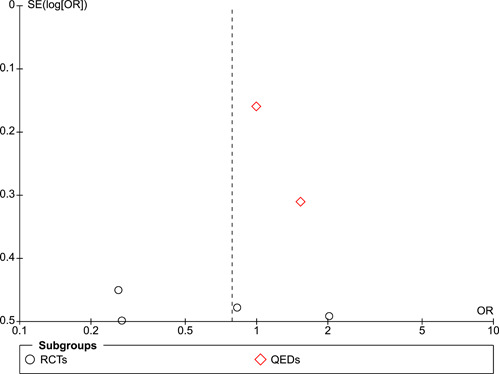
Funnel plot, recidivism 6–12 months.

**Figure 7:**
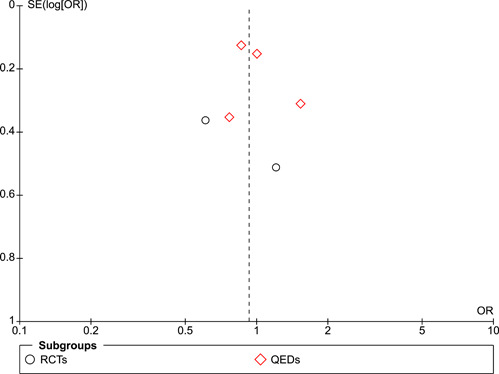
(Analysis 1.3) Funnel plot, recidivism 15–18 months.

We used funnel plots in attempts to assess the likelihood of publication bias and/or small study effects in pair‐wise meta‐analyses. The largest pairwise meta‐analyses in our review included fewer than 10 studies (see Figures 6 and 7), which is insufficient for formal (statistical) analysis of funnel plot asymmetry. Because visual inspection of funnel plot asymmetry is not reliable (Lau, 2006; Terrin, 2005), we cannot draw conclusions about the likelihood of publication bias and/or small study effects based on these funnel plots.

## 6 DISCUSSION

### 6.1 Summary of main results

Twenty studies met our inclusion criteria, including 14 RCTs and 6 QEDs. Fifteen studies provided data for meta‐analysis, based on samples that included a total of 10,980 families. Most (15) of the included studies were conducted in the USA and others were located in Northern Europe and the UK. Half of the included studies were conducted by FFT program developers.

We examined effects of FFT compared with other active treatments on a wide range of outcomes for youth, parents, and families. Studies provided data on outcomes at multiple endpoints, ranging from 2 to 18 months after the beginning of treatment; three studies also provided data on outcomes at approximately 3 years after the beginning of treatment.

Using pairwise meta‐analysis, we found no evidence of effects of FFT compared with other active treatments on a wide range of primary and secondary outcomes. Our Summary of Findings Table showed that FFT was neither more nor less effective than other treatments in reducing recidivism, time in out‐of‐home placements, externalising or internalising behaviours, delinquency, or drug or alcohol use within 1 year after treatment began. There were few studies in these analyses (*k* < 7) and little heterogeneity of effects across studies.

Only two small studies provided evidence on effects of FFT compared with no treatment. Thus, there is insufficient evidence on the absolute effects of FFT.

Robust variance estimates were generated with CE models to include all available data on the relative effects of FFT compared with other active treatments, while accounting for correlations among dependent ESs within studies (*k* = 15, nES = 293). These models provide more powerful overall estimates of effects than pairwise meta‐analysis. The direction of effect estimates was inconsistent across outcome domains in the CE models, but most of these effects were not significantly different from no effect. The overall CE estimate of effects of FFT (SMD = 0.19, SE = 0.09) is small, not significantly different from no effect, and could be accounted for by reporting and publication biases. Prediction intervals suggest that future studies are likely to produce a wide range of estimates of effects of FFT, including both positive and negative effects (from −0.37 to 0.75).

Overall, RCTs produced larger effect estimates than QEDs, but moderator analysis showed that this difference was not statistically significant.

FFT developer involvement and study location were confounded because most developer‐led studies took place in the USA. On average, studies conducted by FFT developers produced larger ESs than independent studies, and studies conducted in the USA had larger ESs than studies conducted elsewhere, but these differences were not statistically significant. The largest average effects were produced in USA developer‐led studies and the smallest average effects came from independent studies outside of the USA.

The use of both pairwise meta‐analysis and CE models in the same review provides distinct but overlapping perspectives on a body of evidence and can enhance overall understanding of the contours and weight of that evidence (Littell, 2021).

### 6.2 Overall completeness and applicability of evidence

None of the studies in our review could demonstrate full reporting of outcomes, because none had prospectively registered protocols. We found evidence of partial reporting or non‐reporting of outcomes or endpoints in 15 of 20 included studies (Figures 2 and 5).

We assessed the risk of publication bias and related biases with data from our two largest pairwise meta‐analyses. With only six studies in each of these funnel plots (Figures 6 and 7), results are not informative.

Overall, the evidence for FFT is sparce, and available studies are not representative of all FFT programs. Anecdotal evidence suggests that well‐established and well‐respected FFT programs were more likely to be evaluated than newer and more unstable FFT programs.

### 6.3 Quality of the evidence

Relatively few studies have provided credible data on the impacts of FFT compared with other active treatments or no treatment. Studies that met our criteria included 14 RCTs and 6 QEDs. Most had relatively small samples (median total sample size *n* = 124, IQR = 90 to 222, *k* = 20). Five of the included studies provided no valid data for meta‐analysis.

We found evidence of incomplete reporting in most (15 of 20) included studies. There were high risks of bias in more than half of included studies on baseline equivalence, support for intent‐to‐treat analysis, selective reporting of outcomes, and COI. All included studies had high risk of bias on at least one indicator. Thus, the certainty of available evidence for FFT is very low.

### 6.4 Potential biases in the review process

We were unable to overcome problems with incomplete reporting and selective reporting of outcomes and endpoints among studies in the review. Authors of three studies provided us with some additional unpublished data, but we did not obtain data on all relevant outcomes. We were unable to obtain any data on several NIDA‐funded clinical trials conducted at the Oregon Research Institute.

Meta‐research shows that positive and statistically significant results are more likely to be reported than negative or non‐significant results (Dwan, 2013, 2014; Pigott, 2013; Song, 2010). Published studies yield larger ESs than unpublished studies (*d* = 0.18, 95% CI: 0.10 to 0.25; Polanin, 2016). Publication and reporting biases lead to overestimates of positive effects in systematic reviews (Kirkham, 2010; Norris, 2012). Thus, our review is likely to have overestimated positive effects of FFT.

### 6.5 Agreements and disagreements with other studies or reviews

For more than 20 years, proponents of FFT have claimed that there is ample evidence that the program has consistent, positive effects across studies. For example, it has been claimed that ‘The original FFT outcome studies, conducted at the University of Utah, provide scientifically sound data that demonstrate the efficacy of the model. Subsequent studies performed elsewhere have enhanced the impressive picture of FFT treatment effectiveness. Taken together, the combination of formal outcome studies and other replications have provided remarkable consistency with respect to FFT across populations, treatment sites, and years’ (Alexander, 1998, p. 55). Our systematic review and meta‐analysis contradicts these claims in several respects: we found that the quality of evidence for FFT is weak, most outcomes are not fully reported, and (despite evidence of select reporting) available evidence does not support claims that FFT has consistent, positive effects across studies. Nevertheless, FFT has an active marketing program that continues to claim that FFT has ‘proven results’ (https://www.fftllc.com/evidence-based-research). Support for this claim comes from data on ‘success rates’ in FFT groups that are presented inaccurately and without relevant comparisons to control groups. For example, Gottfredson 2018 reported that 65% of all FFT cases had no arrests within 18 months after random assignment, compared with 53%–60% of all control cases (results for control cases vary across study reports); overall, between‐group differences are not statistically significant. However, FFT LLC claims that this study shows that 77% of FFT cases had no subsequent arrests and FFT LLC fails to mention results for control cases (https://www.fftllc.com/evidence-based-research).

The largest previous meta‐analysis of research on effects of FFT concluded, ‘FFT is more effective than no‐treatment or well‐defined [alternative treatments], but not more effective than TAU’ (Hartnett, 2017, p. 615). We did not find that FFT was more effective than no treatment or alternative treatments. Differences between these reviews could be explained by the use of different inclusion criteria and different review methods. Unlike the Hartnett (2017) meta‐analysis, we excluded QEDs that lacked statistical controls for baseline differences, because these studies lack adequate controls for selection bias. We also excluded studies with younger or older cohorts, focusing on the age group for which FFT was originally intended. Although our inclusion criteria were more stringent, our meta‐analysis included more studies, more comparisons, more data from multiple study reports, and more robust analytic methods than those in previous meta‐analyses. Hartnett (2017) reported results for RCTs and QEDs separately and further subdivided results by comparison conditions (no treatment, TAU, or alternative treatment); they produced six meta‐analyses with two to five studies in each analysis. We found no consistent differences in results between RCTs and QEDs, so we collapsed results across these study designs to increase statistical power. We found diversity among alternative treatments (individual, family, and group therapies) and little evidence that TAU and alternative treatments differed in terms of amount and duration of services provided, so we collapsed data on all active treatments to increase statistical power. Hartnett (2017) used results of heterogeneity tests to determine whether to use fixed or random effects models in meta‐analysis (contrary to recommendations of Borenstein, 2009, 2010). We used random effect models whenever possible because we cannot assume that all studies are estimating the same (fixed) ES (due to variations across studies in methods, participant characteristics, and comparison conditions). We used robust variance estimates (from CE models) to include multiple dependent ESs in random effects models wherever possible; we used FEs models only when random effects CE models were unreliable. We examined results within outcome domains and then aggregated across domains, with 15 studies and 293 ESs in our final CE analysis. With more stringent eligibility criteria, more studies, more outcome data, and better meta‐analytic methods, we believe that results of our review are more credible than those of previous reviews and meta‐analyses on effects of FFT. Even so, as explained above, we suspect that our overall estimates may be upwardly biased.

## 7 IMPLICATIONS FOR RESEARCH

Our analysis of the best available studies of effects of FFT shows room for improvement. Future studies of effects of FFT (and similar programs) should:

- use large controlled trials or rigorous QEDs that can support causal inferences (e.g., quasi‐experimental studies should use parallel cohorts and controls for differences between groups at baseline);
- follow detailed prospectively‐registered protocols specifying the outcome measures, analyses, and endpoints that will be used;
- use outcome measures that have been validated in the study sample or in similar samples;
- use some source of administrative data that will be available for all participants to reduce biases due to differential attrition;
- use intent‐to‐treat analysis to provide policy‐relevant estimates of treatment effects;
- collect outcome data at multiple points in time to assess possible short‐term and long‐term effects;
- take steps to minimise any risks of bias;
- fully report data on all outcomes, analyses, and endpoints.

Future research should also move beyond branded interventions to identify ‘active ingredients’ or elements responsible for effecting change. Some observers have suggested that the ‘theory of change’ for FFT is not very cogent; that is, it is not clear which aspects of FFT and which therapeutic processes or actions are most important to bring about change.

Future reviews should follow current guidelines for systematic reviews and meta‐analysis, taking all steps necessary to minimise bias and error.

## 8 AUTHORS' CONCLUSIONS

### 8.1 Implications for practice

FFT is actively marketed and widely used in the USA and other high‐income countries, where it is said to have consistent, positive effects on outcomes for youth and families. Claims about the effectiveness of FFT have persisted for decades, despite the fact that the evidence base for FFT is sparce and inconsistent.

Our systematic review and meta‐analysis showed that FFT is not consistently more (or less) effective than the active treatments to which it has been compared, including TAU and other individual, group, and family therapies. There is insufficient evidence to assess effects of FFT compared with specific types of TAU, specific alternative treatments, or no treatment.

Repeated claims about the cost effectiveness of FFT are not reliable, because they are based on incomplete assessments of the effects of FFT. Effect estimates appear to be inflated by reporting biases, leading to cost effectiveness estimates that are also inflated.

This means that the choice between FFT and other interventions can and should be based on factors other than claims about the relative effectiveness or cost‐effectiveness of FFT. For example, some providers appreciate the clinical approach and/or structure of FFT, while others find its structure and staffing requirements onerous; these are good reasons for using or not using FFT.

## CONTRIBUTIONS OF AUTHORS

Julia Littell (JL) drafted the protocol and data extraction forms. Karinne H Nilsen (KN) wrote the search strategy. KN conducted electronic searches and searched for grey literature. Working in pairs Jennifer Roberts (JR), JL, and other contributors screened titles and abstracts. KN and JL retrieved potentially eligible studies. Travis Labrum (TL), JL, and other contributors worked in pairs to make eligibility decisions. JL was paired with other reviewers to extract data from studies. JL conducted descriptive and pairwise meta‐analyses. Terri Pigott (TP) conducted CE analysis. JL produced the first draft of the review, with input from all co‐authors.

## DECLARATIONS OF INTEREST

None known.

## DIFFERENCES BETWEEN PROTOCOL AND REVIEW

Changes made from the protocol reflect advances in systematic review methods since the publication of the protocol for this review (Littell et al., 2007).

**Objectives**

Before examining outcome data, we clarified criteria for outcomes for a Summary of Findings (**SoF**) Table. These outcomes were not specified in our protocol. We added recidivism (arrest or conviction) as a primary outcome.

We adopted newer and more explicit procedures for assessing the risks of bias (**ROB**) in included studies. Our protocol indicated that we would rate: allocation sequence generation, allocation concealment, standardisation and blinding of assessments, incomplete outcome data (exclusions and attrition), and selective or incomplete reporting of outcomes. We also planned to distinguish studies that support intent‐to‐treat analysis from those that provide per‐protocol analyses. These plans were further defined and additional categories were added, based on the Cochrane ROB tool (version 1, Higgins, 2011) and What Works Clearinghouse standards for assessing baseline equivalence and attrition (WWC attrition; WWC baseline). We rated each study on 11 risk‐of‐bias variables and documented reasons for these ratings.

For purposes of pairwise meta‐analysis, we captured multiple **endpoints** in categories that best fit the available data and allowed us to present as much data as possible. We had planned to look at outcomes at 6 months after referral, 1‐year follow‐up, and longer. But we found that included studies collected outcome data in shorter time intervals. Therefore, we examined outcomes in the following intervals: less than 9 months, 9–14, 15–23, and 24–42 months after treatment began.

When multiple reports on a single outcome were available (e.g., parent and youth reports on family cohesion), our protocol indicated that we would average results across **sources** and pool their standard errors. With the advent of newer, multivariate meta‐analysis methods, we were able to include multiple dependent measures in the same analysis, using robust variance estimates (RVE). Because reports from different data sources do not always agree, we selected the most direct measure for use in pair‐wise meta‐analyses (i.e., youth reports on youth outcomes, parent reports on parent and family outcomes).

We added a discussion of the methods we used for handling **missing** data.

We added a statement in the methods section on **unit** of analysis issues, describing our approach to working with studies with multiple arms and cluster‐randomised trials.

Our protocol indicated that we would use **random effects** models if we found statistical evidence of heterogeneity. More recently, experts have argued that the choice between FE and random effects models should be made a priori, based on conceptual considerations (Borenstein, Borenstein 2009, Borenstein 2010). Given the differences between FFT trials (in terms of their methods, sample characteristics, comparison conditions, etc.) we did not expect all studies to be estimating a common ES. Thus, we use random effects models whenever possible, because these models reflect our assumptions about the underlying distribution of ESs in FFT outcomes studies.

To include all relevant ESs in meta‐analysis, while accounting for dependencies among multiple ESs within studies, we used correlated effects (**CE**) models with robust variance estimation, a newer statistical method. This allowed us to synthesise multiple, dependent ESs and assess potential **moderators** of treatment effects. The use of CE models for moderator analysis replaced our earlier plans (described in our protocol) to conduct moderator analysis with older analytic methods (the ANOVA analogue and basic meta‐regression). We also conducted pair‐wise meta‐analysis, to provide clear referents for understanding potential impacts of FFT at specific endpoints. The use of these two analytic models in tandem provides a more robust assessment of effects than either approach would alone.

We dropped plans to combine odds ratios and SMDs in pairwise meta‐analysis, in favour of newer (CE) approaches.

## PUBLISHED NOTES

**Characteristics of studies**

Characteristics of included studies

Alexander, 1973

**Table:** 

**Methods**	From October 1970 to January 1972, 99 families were referred by the Salt Lake County Juvenile Court to the Family Clinic at the University of Utah, and assigned to one of four different treatment conditions. Follow‐up data were available on 86 cases.
	It is not clear who performed random assignment or how this was done. One report indicated that subjects were ‘assigned during randomly selected shifts at the Court’ (Parsons, 1973, p. 197). Other reports indicate that there were ‘minor exceptions [to random assignment] caused by program availability’ (Alexander, 1973, p. 220; Klein, 1977, p. 471). However one group of families was referred to a Mormon church program and, although ‘every attempt was made to assign these families as randomly as possible,’ this program was restricted to Mormon families (in contrast, 70% of the families in the other treatment groups were Mormon; Alexander, 1976, p. 181).
	Not including 13 cases with missing data, 46 families were assigned to FFT and 30 were assigned to two control conditions (19 to client centred family groups and 11 to eclectic‐psychodynamic family treatment) and a no‐treatment control group. It is not clear whether the no‐treatment control group was randomly assigned or randomly selected from a larger pool: ‘An additional 10 randomly selected families were released from the court with no formal treatment…’ (Alexander, 1973, p. 222).
	Authors also compared results with a post hoc no‐treatment comparison group (*n* = 46) and county recidivism rates (*n* = 2800). These groups are not included in our analysis.
	Dependent measures included observations of family interactions obtained in a clinic laboratory setting. No information on the reliability or validity of these measures was provided (hence, these measures are not included in our analysis).
	Recidivism was the primary outcome measure, defined only as ‘rereferral for behavioural offense’ following termination from treatment (Alexander & Parsons, 1973, p. 222). Criminal offenses were also recorded, but not fully reported. For treatment cases, the initial follow‐up period ranged from 6 to 18 months after treatment; it is not clear how observation periods were defined for no‐treatment control cases. A 2.5‐ to 3.5‐year follow‐up study was also conducted, using recidivism data from computerised juvenile court records, but outcomes are reported only for the younger sibling of referred youth (Klein et al., 1977).
**Participants**	Participants were youth, ages 13–16, who had been arrested or detained at for a behavioural offense, including those who had: run away; been declared ungovernable; been habitually truant; been arrested for shoplifting; or been arrested for possession of alcohol, soft drugs, or tobacco (Alexander & Parsons, 1973, p. 220).
	Only 44% (of the 86 youth with follow‐up data) were male. No information was provided on family socioeconomic status or family composition.
	One published report (e.g., Parsons & Alexander, 1973) provides data on the first 40 cases (20 FFT cases, 10 famines in client‐centred groups, and 10 families who received no treatment).
**Interventions**	**FFT** (then called ‘short term family behavioural treatment’) focused on modifying family interactions (Alexander & Parsons, 1973). Families were seen in a university lab/clinic setting with one‐way mirrors. Therapists [LAB CONTEXT] by differentiating rules from requests, establishing a token economy system, using social reinforcement, and selective use of bibliotherapy (i.e., a behaviour modification primer; Parsons & Alexander, 1973, pp. 196–197). Token economy and bibliotherapy components were used with some but not all families (Alexander & Parsons, 1973, p. 221). No information was provided on the amount of services provided to families.
	FFT therapists were 18 first and second year clinical psychology students, provided with 4 weeks of pre‐service training, live supervision (via one‐way mirror observation), and bi‐weekly group supervision. Overall, FFT therapists received an average of 6 h of training and supervision per week.
	One comparison condition involved ‘**client‐centred family groups**… representative of treatment in many juvenile centers;’ these groups involved didactic discussion of ‘attitudes and feelings about family relationships and adolescent problems’ (Alexander & Parsons, 1973, p. 221). Group treatment was reported lasted the same amount of time as FFT (no details provided). The two therapists, hired by the court, were a 5th year graduate student and recent PhD; it is not clear whether or how they were supervised.
	A second comparison condition, **eclectic, psychodynamic family counselling**, was provided by a Mormon church and ‘represented exactly the form of treatment a significant proportion of teenagers in Salt Lake County receive upon referral to the court… [R]eferrals were made through local clergy to a separate agency’ (Alexander & Parsons, 1973, p. 222). Staff were MSWs and PhDs who had more clinical experience that FFT therapists. They provided an average of 12–15 sessions per family.
	**No treatment control cases** ‘were released from the court with no formal treatment’ (Alexander & Parsons, 1973, p. 222).
	For each group, the intervention phase lasted 4 weeks. During this period, ‘families were not visited by caseworkers or probation officers, and contact with Juvenile Court was suspended’ (Parsons & Alexander, 1973, p. 198).
**Outcomes**	Data on **recidivism** (i.e., re‐referral for a behavioural offence) and **criminal offences** were derived from juvenile court records. Data on recidivism were reported for each group at the first follow‐up (6–18 months after treatment), but criminal offenses are only partially reported (outcome data are not provided for specific comparison and non‐treatment control groups). Data on recidivism (only) at 2.5–3.5 years after the end of treatment were provided for younger siblings of focal youth (only). Observation periods were highly variable across cases. No information was provided on how data were derived from court records or on the reliability of data extraction.
	Several measures of family processes were derived from audiotapes and observations of family members engaged in three tasks and discussion in the University clinic/lab over a period of 20 min. No information is provided on the reliability or validity of these measures, hence they are not included in our analysis.
**Notes**	This study is represented as multiple (separate) studies in some published reviews (e.g., Alexander, Alexander 1998, Alexander 2013, Robbins et al., 2016).
	Parsons and Alexander (1973) reports on the first 40 cases in this study.
	Funding sources: United States Department of Health, Education and Welfare (Grant 72‐P‐40061/9‐01); Utah Research Committee (Grant 774‐180‐13).

Risk of bias table

**Table:** 

Bias	Authors' judgement	Support for judgement
Random sequence generation (selection bias)	High risk	'With minor exceptions caused by program availability, families were randomly assigned upon detention to either the treatment program, comparison groups, or a no‐treatment control condition’ (Alexander & Parsons (1973); p. 220).
		Elsewhere, authors note that 10 no‐treatment control cases were ‘randomly selected’ (Alexander & Parsons, 1973, p. 222).
		Another report states that participants were ‘assigned during randomly selected shifts at the Court’ (Parsons & Alexander, 1973; p. 197).
		A third report states that one of the comparison groups was restricted to Mormon families (in contrast, Mormon families made up 70% of cases in the other treatment groups; Alexander & Barton, 1980, p. 181)
		No further information is available on sequence generation methods.
Allocation concealment (selection bias)	High risk	Service availability affected assignments, as did families’ religious affiliations.
		No further information is available on allocation concealment methods.
Baseline equivalence	High risk	Data on age and gender are only provided for first 40 cases. No information is provided on race/ethnicity. All (11) families in the eclectic‐psychodynamic family treatment group were Mormon, compared with 70% of families in other groups (Alexander & Barton, 1976, p. 181)
Performance bias (confounding)	High risk	The clinicians who worked with families in treatment different groups had different backgrounds and received different amounts of training and supervision (Alexander & Parsons, 1973, pp. 221–222).
Detection bias (blinding of assessment): administrative data	Unclear risk	Data on recidivism were derived from juvenile court records. It is not clear who derived this data or how outcomes were defined and coded.
Detection bias (blinding of assessors): participant reports	Unclear risk	
Attrition bias: administrative data	Unclear risk	13% attrition overall; insufficient information to assess differential attrition.
Attrition bias: participant reports	Unclear risk	
Standardised observation periods	High risk	Data on recidivism was collected from juvenile court records ‘following termination of treatment’ (Alexander & Parsons, 1973, p. 222). The follow‐up observation period ranged from 6 to 18 months after treatment; it is not clear how the observation period was defined for no‐treatment control cases. ‘The follow‐up period for individual families varied widely, but across groups the period was comparable’ (Alexander & Parsons, 1973, p. 223). Follow‐up observations occurred between 2.5 and 3.5 years after intervention (Klein et al., 1977).
Intention‐to‐treat	High risk	A total of 99 families were referred for assignment. ‘Subsequent to the program, follow‐up records were available only on 86 families’ (Alexander & Parsons, 1973, p. 220). No information is available on differential attrition.
Selective reporting (reporting bias)	Unclear risk	There is no protocol for this study. Three‐year follow‐up data are provided for siblings only, not for focal youth. Criminal arrest data provided for FFT group, but not for comparison groups at 6–18 months.
Validated outcome measures	Unclear risk	‘Recidivism’ was defined as ‘rereferral for a behavioural offense’ following termination of treatment; unclear how the observation period was defined for no‐treatment cases. There is no information on the reliability or validity of this outcome measure.
Conflicts of interest	High risk	Dr. Alexander is a founder of FFT.

Baglivio, 2014

**Table:** 

**Methods**	Propensity score matching (PSM) was used to create comparable groups of youth who received MST or FFT services in the state of Florida (USA) from July 2009 through June 2011. Using nearest‐neighbour matching without replacement, PSM was based on gender, race, region of the state, age at admission to MST/FFT service, overall risk to reoffend (using the Positive Achievement Change Tool, PACT), criminal history, a global ‘social risk’ measure, most serious prior offense, age at first offense, antisocial peer association, alcohol use, drug use, and parental authority (2014, p. 1038).
	Archival data from Florida's Juvenile Justice Information System (JJIS) were used to identify offenses; both juvenile and adult records were included.
**Participants**	Participants had been referred to the Florida Department of Juvenile Justice (FDJJ). Juvenile referrals to the FDJJ are similar to adult arrests (Baglivio et al., 2017, p. 1036).
**Interventions**	The average length of service was 119 days for MST and 95 days for FFT (t = 14.005, *p* < 0.001; Baglivio et al., 2014, p. 1038).
**Outcomes**	Outcomes include (1) new arrests or violations of probation that occurred during services and (2) adjudication or conviction for an offense that occurred during the 12 period after services ended.
	Our analyses included results for the matched pairs of 628 youth in each group (*n* = 1256 total).
**Notes**	

Risk of bias table

**Table:** 

Bias	Authors' judgement	Support for judgement
Random sequence generation (selection bias)	High risk	Non‐random allocation to groups.
Allocation concealment (selection bias)	High risk	Allocation was not concealed.
Baseline equivalence	Unclear risk	Initial baseline differences between groups were controlled with propensity score matching, leaving no differences with *d* > 0.25. However, region was coded 1 = North, 2 = Central, 3 = South, with substantial between‐group differences before PSM (more FFT cases in South Florida, more MST cases in the North); and balancing these groups on regional scores does not mean that the groups were balanced across the regions.
Performance bias (confounding)	Unclear risk	Cases in FFT received services over a shorter period than those in MST (mean of 95 vs. 119 days; *d* = 0.79). Authors noted that length (and intensity) of services may relate to severity of family problems or difficulty of engagement (2014, p. 1038).
Detection bias (blinding of assessment): administrative data	Unclear risk	No discussion of blinding of assessors.
Detection bias (blinding of assessors): participant reports	Unclear risk	
Attrition bias: administrative data	Low risk	Reliance on cases with administrative data.
Attrition bias: participant reports	Unclear risk	
Standardised observation periods	High risk	Observation periods vary, depending on length of service, which was significantly shorter for FFT vs. MST cases (2014, p. 1038).
Intention‐to‐treat	Low risk	'As the intent was to examine outcomes for all youth *referred* to the services…’ both program completers and dropouts were included in analyses (2014, p. 1037).
Selective reporting (reporting bias)	Unclear risk	There is no public protocol for this study.
Validated outcome measures	Low risk	Archival data used to identify criminal offences.
Conflicts of interest	Unclear risk	No statement on potential conflicts of interest.

Barnoski 2002

**Table:** 

**Methods**	Washington State court staff assessed all juvenile offenders for eligibility for services and referred some ‘moderate or high risk’ youth to FFT. Enrolment into this first phase of this study occurred from January 1999 to September 2001 in 14 counties in Washington State (USA): Benton/Franklin, Grant, Grays Harbor, King, Kitsap, Klickitat, Lincoln, Pierce, Skagit, Snohomish, Spokane, Thurston, Whatcom, and Yakima counties.
	During this period, youth who were referred to FFT when the program was full were assigned by court staff to a ‘waiting line’ comparison group that did not received FFT services (Barnoski, 2002, 2004a). Sexton and Turner (2010) describe results that build on the Barnoski study with an ‘expanded sample’. The 2010 report is described as a randomised experiment, using ‘a stratified randomisation procedure at the county level…as caseload openings permitted’ (p. 341).
	Barnoski (2002, 2004a) reported that cases in the FFT group were limited to those served by therapists who had a minimum of 90 days of supervised service; these FFT cases were further divided into two subgroups: those served by therapists deemed to be ‘competent’ and ‘not competent’. Cases seen by ‘competent’ therapists were more likely to be White, compared with cases seen by non‐competent therapists (80% vs. 71%, *χ* ^2^= 3.87, *p* = 0.049). We used data from all FFT cases in meta‐analysis.
	Data on recidivism (conviction for a subsequent offence) were obtained from administrative records kept by the state courts and Department of Corrections at 12 and 18 months after referral.
	Data from initial assessments were used as statistical controls for baseline differences between FFT and TAU groups in age, gender, and risk factors.
**Participants**	The 2002 and 2004(2004a and 2004b) reports include 700 juvenile offenders. Most (79%) were male, with an average age of 15.3; 75% were White, 11% Black.
	The 2010 report includes a total of 917 juvenile offenders.
**Interventions**	FFT versus TAU traditional probation services. No information on amounts or types of services provided in FFT or TAU.
**Outcomes**	Recidivism, defined as conviction for a subsequent offence within 12 or 18 months after group assignment. Other outcome measures were limited to felony crimes and violent felony crimes.
**Notes**	Most of the analysis in the primary (2004b) study focuses on differences in outcomes for youth served by ‘competent’ versus ‘non competent’ FFT therapists, using subjective judgements of therapist competence. Similarly, the 2010 report analyzes results by high and low FFT therapist adherence. These comparisons are not included in our analysis.
	Costs of FFT per family were approximately $2100 USD (Barnoski 2004a). Cost‐effectiveness analyses are based on felony recidivism outcomes which favoured FFT, not total recidivism (no significant between‐group differences) or violent felony recidivism (results favoured TAU).
	Funded by the Washington State Institute for Public Policy and by a grants to Dr. Sexton from the US National Institute on Drug Abuse (R01DA023165, R01‐DA017218‐01A1).
	Thomas Sexton was the president of Functional Family Therapy Associates.

Risk of bias table

**Table:** 

Bias	Authors' judgement	Support for judgement
Random sequence generation (selection bias)	High risk	Non‐random assignment to FFT or wait list comparison group.
		‘When the program reached capacity (all therapists had full caseloads or sessions were full), the remaining eligible youth were assigned by court staff to the control group and never participated in the program; instead, they received the usual juvenile court services’ (2004a, p. 4).
		‘The procedures for this assignment process varied from court to court. In some courts, the assignment of youth was random (using the last digit of their juvenile number), in some courts it occurred on a first‐come, first‐served basis, while in others, the courts exercised some discretion in group assignments’ (2004a, p. 4).
		The 2010 report describes ‘a stratified randomisation procedure at the county level…as caseload openings permitted’ (p. 341). It provides no details on these procedures.
Allocation concealment (selection bias)	High risk	'Assignment decisions [were made] by the juvenile court staff’ (2004b, p. 1).
Baseline equivalence	High risk	'Youth viewed as most in need of services may have received preferential assignment to FFT rather than the control group, and the higher‐risk youth may have received preferential assignment to the better therapists’ (2004a, p. 7). There were no significant differences between FFT and control cases on race or age.
Performance bias (confounding)	Unclear risk	No information on amounts or types of services provided to FFT and wait‐list comparison groups.
Detection bias (blinding of assessment): administrative data	Unclear risk	Recidivism data obtained from databases maintained by State Courts and Department of Corrections.
Detection bias (blinding of assessors): participant reports	Unclear risk	
Attrition bias: administrative data	Unclear risk	No information on case flow through the study (number referred, number excluded from analysis, etc.)
Attrition bias: participant reports	Unclear risk	
Standardised observation periods	Low risk	Recidivism was assessed at 18 months post‐treatment assignment.
Intention‐to‐treat	High risk	For purposes of the study, FFT cases were limited to those served by clinicians who had at least 90 days of supervised experience.
Selective reporting (reporting bias)	Unclear risk	No protocol for the study. 2010 paper only reports between‐group comparisons on felonies. Cost effectiveness analyses are based only on felony recidivism.
Validated outcome measures	Low risk	Administrative data (from state courts and Department of Corrections) on recidivism.
Conflicts of interest	Unclear risk	No conflict of interest statement for reports produced in 2002, 2004a, or 2004b; 2010 report states that Thomas Sexton was the president of Functional Family Therapy Associates.

Carr et al., 2014

**Table:** 

**Methods**	Between 2012 and 2014, 82 families were randomly assigned to FFT (*n* = 27) at Archways Families First or TAU (*n* = 55) provided by referring agencies in Dublin, Ireland. Referring agencies included the Health Service Executive (37%), schools (31%), community agencies (17%), the Department of Education's behavioural support service (7%), the Irish Youth Justice Service (4%), and other sources (5%).
	‘Minimization procedures were used to reduce differences between treatment and control group cases on age, gender, family composition (one‐ or two‐parent family), and SDQ subscale profile. Small groups of 3–6 cases were matched as closely as possible on these variables, and then randomly assigned to treatment and control groups at a ratio of 2:1’ (2016, p. 289).
	Cases were assessed at baseline (T1) and 17–20 weeks (T2). FFT cases (only) were also assessed 3 months after T2 (T3).
	After T2, cases ‘exited the control group’ (2014, p. 17); 11 control cases were lost to follow‐up, 14 were excluded from further study because they didn't meet the study's eligibility criteria, and 15 of the remaining 30 control cases were ‘randomly assigned’ to FFT.
	Authors report that they used ‘intent‐to‐treat analysis…with last‐observation carried forward where data were missing at Time 2 or 3’ (2014, p. 22); however, 15 cases that were originally assigned to the control group and later received FFT are double‐counted (i.e., they are counted in the control group AND in the FFT group) at baseline, T2, and T3. Results are provided for 97 ‘families’ (2014, p. 19; 2018, p. 5), even though the total number of families is 82.
	Because the FFT and control samples overlap, we could not use data from this study in meta‐analysis.
**Participants**	Eligibility criteria specified that parents scored at or above the clinical cut‐off (of 17) on the total difficulties scale of the Strengths and Difficulties Questionnaire (SDQ).
**Interventions**	Provided by Archways Families First, FFT consisted of approximately 20 sessions over 4–5 months.
	The control group received TAU from referring agencies until they were lost to follow‐up (*n* = 11) or completed T2 assessments (*n* = 44).
**Outcomes**	Adolescent behaviour problems were assessed with parent and adolescent versions of the SDQ; Family functioning was assessed with the Systemic Clinical Outcomes and Routine Evaluation—28 (SCORE) and the parent and adolescent versions of the revised Client Outcome Measure (COM).
**Notes**	The study was funded by Archways, an Atlantic Philanthropies grantee and a recipient of funding from the Irish Youth Justice Service.

Risk of bias table

**Table:** 

Bias	Authors' judgement	Support for judgement
Random sequence generation (selection bias)	High risk	Initially, 27 cases were randomly assigned to FFT and 55 were assigned to the control group; then, at T2, 14 (of the 55) control cases were dropped from further study because they didn't meet eligibility criteria, 11 control cases were lost to follow‐up, and 15 of the remaining 30 control cases were ‘randomly assigned’ to the FFT group. Random assignment was performed on small groups or pairs of ‘similar’ cases. Sequence generation methods were not described.
Allocation concealment (selection bias)	Unclear risk	No discussion of allocation concealment.
Baseline equivalence	Unclear risk	Data from 15 cases are included in the baseline data on *both* FFT and control groups. The appearance of the same cases in both groups renders between‐group comparisons meaningless.
Performance bias (confounding)	High risk	No information on amounts of service provided. Control cases that later received FFT are included in both groups; these cases remained in the study longer than other families.
Detection bias (blinding of assessment): administrative data	Unclear risk	
Detection bias (blinding of assessors): participant reports	Unclear risk	No discussion of blinding of assessors.
Attrition bias: administrative data	Unclear risk	
Attrition bias: participant reports	High risk	Attrition at T2 was 7% for FFT and 20% for control cases. Attrition at T3 was 48%.
Standardised observation periods	High risk	15 control cases were assessed at T1 and T2, and then reassessed in another round of T1 and T2 (in some cases, T3) interviews as FFT cases. The timing of these assessments was not parallel to assessments for other cases.
Intention‐to‐treat	High risk	15 cases were assigned the control group, later assigned to FFT, and their outcomes were included in reports for both groups. 14 (of 55) control cases were excluded from data collection because they did not meet study eligibility criteria.
Selective reporting (reporting bias)	Unclear risk	There is no public protocol for this study.
Validated outcome measures	Unclear risk	No information was provided on the reliability or validity of outcome measures in this sample.
Conflicts of interest	High risk	Thomas Sexton, one of the founders of FFT, is a co‐author.

Celinska, 2013

**Table:** 

**Methods**	Between 2006 and 2011, youth (ages 11–17) living in Middlesex County, New Jersey (USA) enroled in FFT or Youth Case Management (YCM).
	The Strengths and Needs Assessment (SNA) was administered before and after treatment.
	Court records were used to identify delinquency outcomes within 1 year after treatment completion.
**Participants**	The study sample was limited to youth who completed treatment, completed pre‐ and post‐treatment SNAs, and for whom court records were available (*n* = 107 FFT cases and 48 youth YCM cases). This sample is drawn from a larger pool of 141 FFT cases and 105 YCM cases.
	‘Youth who were in the [YCM] comparison group were matched with those who received FFT using a pre‐ and post‐matched comparison group design’ (2019, p. 258).
	The first author trained YCM case managers to identify eligible youth (those were at risk for delinquent behaviour and had been involved with Family Court, probation, detention, Division of Youth and Family Services, and/or a Family Crisis Intervention Unit).
	The study sample was 56% male; 57% white, 28% Black, 4% Asian, and 11% other races; 28% were identified as Hispanic. Mean age was 15.5.
**Interventions**	FFT services were provided by the Children at Risk Resources and Interventions—Youth Intensive Intervention Program (CARRI‐YIIP). This FFT program was structured in 3 phases.
	Youth in the YCM comparison group received individual therapy (45 cases) or mentoring (3 cases) from 10 service providers in the county.
	Length of services is described in ‘months’ (2019, p. 264), but given the nature of FFT we assume authors meant ‘weeks.’ FFT lasted an average of 16.4 [months/weeks] (SD 7.2), compared with 18.6 [months/weeks] in YCM (SD 20.2).
**Outcomes**	The Strength and Needs Assessment (SNA) was completed by therapists to assess seven domains: life domain functioning, child strengths, acculturation, caregiver strengths, caregiver needs, child behavioural/emotional needs, and child risk behaviours.
	Court records were used to determine whether youth were sanctioned, convicted, or institutionalised for a new offence within 1 year after program completion. In addition to any new criminal offenses, researchers identified new violent crimes, property crimes, and drug crimes.
**Notes**	No information on funding sources.
	The 2019 report on this study contains at least one and possibly two reporting errors: (1) In Table 5 (p. 269) the effect of treatment group on drug offenses at 1 year post‐intervention is reported as OR = 0.013 with a 95% confidence interval (CI) of 0.02 to 0.08; this is not possible, as the CI must include the estimate; hence, we did not include this information in our analyses. (2) In Table 4, differences in the proportion of cases with drug offenses at 1 year post‐intervention (37.5% of control cases and 1.9% of treatment cases) yield an OR = 31.5 (RR > 20), an unusually high outlier. We conducted sensitivity analysis to determine whether this outlier affected overall results in the CE models.

Risk of bias table

**Table:** 

Bias	Authors’ judgement	Support for judgement
Random sequence generation (selection bias)	High risk	Non‐random allocation to groups.
Allocation concealment (selection bias)	High risk	Non‐random allocation to groups (referrals by case management staff).
Baseline equivalence	High risk	Compared with YCM, FFT cases included more males (60% FFT vs. 48% YCM; *d* = 0.27); more people of races other than Black, Asian, and White (13% vs. 6%; *d* = 0.45); and FFT cases had fewer prior convictions for drug offences (*d* = 0.35), more violent offences (*d* = 0.42) and more technical offences (*d* = 0.44) (based on data from 2019, p. 263).
Performance bias (confounding)	Unclear risk	Little information on service delivery. The length of control services was longer on average and more variable than FFT.
Detection bias (blinding of assessment): administrative data	Unclear risk	No discussion of blinding of assessors.
Detection bias (blinding of assessors): participant reports	High risk	Strengths and needs assessments were completed by therapists.
Attrition bias: administrative data	Unclear risk	37% attrition, 30% differential attrition. (Exclusion of 28 FFT and 10 YCM cases that did not complete treatment, 45 YCM cases with missing court data, 6 FFT and 2 YCM cases with missing SNA data; 2019, p. 259).
Attrition bias: participant reports	High risk	37% attrition, 30% differential attrition. (Exclusion of 28 FFT and 10 YCM cases that did not complete treatment, 45 YCM cases with missing court data, 6 FFT and 2 YCM cases with missing SNA data; 2019, p. 259).
Standardised observation periods	High risk	The 12 month observation period began at the end of treatment (discharge). Treatment lengths varied considerably (esp. in the comparison group) and were longer on average for youth in the YCM vs. FFT group (YCM mean = 18.6 months, SD = 20.2; FFT mean = 16.4, SD = 7.2; 2019, p. 263).
Intention‐to‐treat	High risk	Systematic exclusion of cases that did not complete treatment (28 FFT cases and 10 YCM cases), those with missing court data (45 YCM cases), and those with missing SNA data (6 FFT and 2 YCM cases).
Selective reporting (reporting bias)	Unclear risk	No public protocol is available.
Validated outcome measures	Unclear risk	Most scales had low internal consistency in this sample (*α*s < 0.7; 2019, p. 261). No information on inter‐rater reliability of coded court records.
Conflicts of interest	Unclear risk	No statement regarding conflicts of interest.

Darnell, 2015

**Table:** 

**Methods**	Propensity score matching (PSM) was used to create four statistically equivalent groups who received different treatments shortly after their release from court‐ordered out‐of‐home placements (OHP). Three experimental treatments were provided between 2007 and 2012: (1) FFT, (2) Functional Family Probation (FFP), and (3) the combination of FFT plus FTP; (4) a comparison group received conventional probation services between 2005 and 2012 (p. 77).
	Variables used in PSM were gender, race/ethnicity, age at release from recent placement, age at first arrest, age at first felony, age at first OHP, count of prior arrests, count of prior OHPs, two variables representing geographic divisions of the service area, and counts of prior petitions of various types (p. 77).
**Participants**	Participants were predominantly Latino and African American youth, ages 11–18, who had been recently released from a court‐ordered placements in foster care, group homes, and psychiatric hospitals. After release, all youth were under probation supervision and returned home to live with their families (p. 77).
**Interventions**	For purposes of our review, relevant comparisons are FFT vs. probation services as usual (TAU).
	**FFT** was structured in five phases (Engagement, Motivation, Relational Assessment, Behaviour Change, and Generalisation) and provided by clinicians trained in the FFT model. After baseline assessment, youth and families met weekly with the FFT therapist. Successful completion occurred by mutual agreement. The average number of FFT sessions was 9.1, the average time to completion was 4.2 months (p. 77).
	**Probation Services as Usual**. ‘Youth who received standard probation supervision were assigned a PO who carried between 75 and 150 cases. POs were required to conduct one face‐to‐face contact with the youth per month, which could occur at the probation office, at school, or in the community. Additional support services that could be incorporated included school‐based services, programs focused on gang membership, gender‐specific services, and drug and mental health courts. Data on number of contacts and duration of standard probation supervision were not available’ (p. 77).
**Outcomes**	The primary outcome was subsequent out of home placement (OHP). ‘Data were extracted from administrative data systems for juvenile justice and child welfare departments’ (p. 77).
**Notes**	The study was funded by the Casey Family Program.

Risk of bias table

**Table:** 

Bias	Authors’ judgement	Support for judgement
Random sequence generation (selection bias)	High risk	Non‐random sequence generation.
Allocation concealment (selection bias)	High risk	Allocation was determined by probation supervisors’ judgement and service availability (p. 77).
Baseline equivalence	Low risk	Baseline equivalence was achieved through PSM with d < 0.25 for measured between‐group differences.
Performance bias (confounding)	Unclear risk	No data on amounts and types of services provided to the conventional probation group.
Detection bias (blinding of assessment): administrative data	Unclear risk	No discussion of blinding of assessment.
Detection bias (blinding of assessors): participant reports	Unclear risk	
Attrition bias: administrative data	Unclear risk	Mean imputation was used for all variables with missing data (p. 77).
Attrition bias: participant reports	Unclear risk	
Standardised observation periods	Low risk	Observations through 36 months after court‐ordered placement ended.
Intention‐to‐treat	High risk	Sample only includes treated cases.
Selective reporting (reporting bias)	Unclear risk	No public protocol available.
Validated outcome measures	Low risk	Use of administrative data on out of home placements.
Conflicts of interest	Unclear risk	No statement on conflicts of interest.

Dunham, 2009

**Table:** 

**Methods**	Juvenile offenders who received FFT were compared with offenders who did not receive any family‐based treatment. Both groups were involved in juvenile justice diversion programs in Miami‐Dade county (Florida, USA) in 2004‐2005. Attempts were made to match the two groups on demographic characteristics and criminal history; but there were substantial between‐group differences on gender, race/ethnicity, and severity of criminal history; as a result, some outcome analyses controlled for pre‐treatment CSI scores.
	Outcomes on recidivism and criminal charges were reported at 12 months after treatment ended (but duration of treatments varied from 3–7 months).
**Participants**	Participants were involved in juvenile justice diversionary programs in 2004‐2006, had criminal history data (in Miami‐Dade Juvenile Services Department system) for 2004‐2007, had no more than 9 previous criminal charges, and no more than 6 post‐treatment charges (pp. 100–101).
	FFT cases (*n* = 111) were included if they (1) attended at least one FFT session, (2) were seen by a therapist who had been providing FFT services for at least 1 year and had served a total of at least 50 FFT cases, (3) had valid data on FFT program completion, and (4) completed the Youth Outcome Questionnaire (YOQ).
	Comparison cases (*n* = 111) had been referred to probation services that were not family based, and had started those services.
**Interventions**	FFT was provided by 10 therapists (9 were female) at the Institute for Child and Family Health (ICFH) who had each provided FFT services for over 1 year and to a total of at least 50 cases (p. 99). FFT was structured in three phases (engagement and motivation, behaviour change, and generalisation; pp. 106–107) and offered approximately once per week for 12‐19 weeks.
	Probation services could include individual therapy, substance abuse treatment, psycho‐educational groups, social skills enhancement, community work service, victim/offender mediation, coordination of restitution payments, case management services, and alternative sanctions and treatment plans (community work service, coordination of restitution payments, writing apology letters to victims, and increased curfews). The duration and requirements of probation varied widely (p. 108). One diversion program provided after‐school services 6 days a week for 5–7 months; another provided case management services for 3–6 months (p. 104).
**Outcomes**	Outcome data were provided by the Miami‐Dade County Juvenile Services Division (JSD) and (for FFT cases only) the Institute for Child and Family Health (ICFH) for youth.
	Recidivism was defined as the commission of one or more crimes during treatment or within the 12‐month period following the completion of treatment.
	The Crime Severity Index (CSI) was used to calculate the number of crimes committed multiplied by the severity of the type of crime committed.
	Data on program completion were provided by ICFH for FFT cases and by JSD for comparison cases. YOQ data were collected for FFT cases only.
**Notes**	

Risk of bias table

**Table:** 

Bias	Authors’ judgement	Support for judgement
Random sequence generation (selection bias)	High risk	Non‐random assignment to groups.
Allocation concealment (selection bias)	High risk	Cases were referred to FFT by juvenile probation officers.
Baseline equivalence	High risk	The FFT group had a larger proportions of females (37% vs. 24%, *d* = 0.33), more Black youth (45% vs. 32%, *d* = 0.29), and fewer Hispanics (40% vs. 58%, *d* = 0.40). FFT cases had higher pre‐treatment CSI scores (more severe criminal histories) than comparisons cases (p. 118; *d* = 0.41).
Performance bias (confounding)	High risk	FFT cases were limited to those seen by therapists who had been providing FFT for at least a year and to a minimum of 50 cases; no such restrictions were placed on control cases.
Detection bias (blinding of assessment): administrative data	Unclear risk	No discussion of blinding during data collection.
Detection bias (blinding of assessors): participant reports	Unclear risk	
Attrition bias: administrative data	Unclear risk	The sample was restricted to cases with available data; it is not clear how many cases were lost due to missing data.
Attrition bias: participant reports	Unclear risk	
Standardised observation periods	High risk	Outcomes were assessed at 12 months following treatment completion, but the duration of treatments varied (from approximately 3 to 7 months).
Intention‐to‐treat	High risk	Sample was restricted to youth who started treatment.
Selective reporting (reporting bias)	Unclear risk	There is no publicly available protocol.
Validated outcome measures	Unclear risk	Use of administrative data on criminal charges. No information on reliability/validity of the CSI.
Conflicts of interest	Unclear risk	This dissertation research was directed by Thomas Sexton, one of the developers of FFT and a partner of Functional Family Therapy Partners (functionalfamilytherapy.com).

Eeren, 2018

**Table:** 

**Methods**	Between 2009 and 2014, 1714 adolescents and their families started FFT or MST at De Viersprong, an institute for personality disorders and behavioural problems in the Netherlands. After finishing treatment, 697 (41%) of parents and youth completed the primary outcome measure, the CBCL/YSR. Other outcomes (youth living arrangements, school/work involvement, new police contacts) were reported to researchers by the FFT and MST therapists.
	Propensity score matching (PSM) was used to create groups that were statistically equivalent on baseline measures. OLS and logistic regression were used to calculate treatment effects on continuous and dichotomous outcome variables.
**Participants**	Most youth participants were Dutch (85%), 60% were male, 42% were living with one parent, 76% were engaged in school or work at least 20 h per week, and 47% had prior court orders at baseline.
**Interventions**	FFT and MST therapists were trained and licensed to deliver these treatments. They received weekly supervision from a local supervisor and from an independent consultant for the FFT or MST program.
	For cases that completed treatment, the mean duration of FFT was 196.2 days; the mean duration of MST was 148.5 days.
**Outcomes**	The primary outcome was externalising problem behaviour reported by youth and parents (CBCL YSR). Secondary outcomes were dichotomous variables (reported by FFT and MST therapists) indicating whether youth were living at home, engaged in school or work, and had any new police contacts since the beginning of treatment.
	Measures of parenting stress and youths’ internalising problems (CBCL/YSR reports) were reported at baseline, but not post‐treatment.
**Notes**	

Risk of bias table

**Table:** 

Bias	Authors’ judgement	Support for judgement
Random sequence generation (selection bias)	High risk	Non‐random assignment to treatments.
Allocation concealment (selection bias)	High risk	Allocation choices were not concealed.
Baseline equivalence	Unclear risk	PSM was used to create groups with comparable baseline characteristics (after PSM, *d*s < 0.25). Before PSM, there were substantial between‐group differences in baseline scores on youths’ internalising, externalising, and total behavioural problems, gender, country of birth, level of education, previous treatment, engagement in school or work, previous court orders, previous police contact, caregiver's country of birth, and caregiver employment status; youth in FFT were more likely that those in MST to be female, Dutch, living with two parents, and less likely to have prior court orders.
Performance bias (confounding)	Unclear risk	No information provided on number of sessions or hours of contact. FFT lasted somewhat longer (196.2 days on average) than MST (mean of 148.5 days).
Detection bias (blinding of assessment): administrative data	Unclear risk	
Detection bias (blinding of assessors): participant reports	High risk	FFT and MST therapists reported data on three treatment outcomes (youth living arrangements, school/work involvement, and police contacts), in consultation with the caregiver.
Attrition bias: administrative data	Unclear risk	
Attrition bias: participant reports	Unclear risk	The sample was limited to participants who completed primary outcome measures (41% of those who started FFT or MST treatments).
Standardised observation periods	Unclear risk	Outcome data were obtained at the end of treatment; lengths of treatment varied.
Intention‐to‐treat	High risk	Analysis sample excluded participants who did not complete treatment, baseline assessments, and the primary outcome measure.
Selective reporting (reporting bias)	High risk	There is no public protocol for this study. Measures of parenting stress and youth internalising problems were reported at baseline but not post‐treatment.
Validated outcome measures	Unclear risk	Use of standardised CBCL/YSR measures, along with therapist reports on dichotomous outcomes (no information on reliability of the latter).
Conflicts of interest	Low risk	The authors declared that they had no conflicts of interest (p. 1048).

Gottfredson, 2018

**Table:** 

**Methods**	From September 2013 to February 2016, in one Family Court room in Philadelphia (Pennsylvania, USA), youth who were ordered to participate in ‘family services’ as a condition of their probation were randomly assigned to FFT‐G versus another family therapy program.
	Data were collected via interviews with youth and parents at intake and 6 months, and from court records (on justice system contacts and residential placements) and Medicaid records on community services.
**Participants**	Participants were 129 males, ages 11–17 (mean age = 15.4), in predominantly low‐income families (median household income = $17,500 USD, 44% household income below $13,000 USD, 83% received public assistance). One‐quarter of their primary caregivers were married, 80% were African‐American and 19% were Hispanic/Latino (Gottfredson et al., 2018).
	At intake, only 15% of the sample reported that they had ever been a gang member and 7% reported current gang membership (Thornberry et al., 2018).
**Interventions**	FFT‐G incorporates all features of FFT and was adapted for use with youth at risk of gang involvement. Adaptations included efforts to address pressure from neighbourhood gang members and to engage families of gang‐involved youth (Gottfredson et al., 2018, p. 940). FFT LLC staff produced the manual for FFT‐G and provided the training and initial supervision for therapists in 3 social service agencies that were licensed to provided FFT.
	TAU involved an alternative family therapy program that was approximately the same intensity and duration as FFT (p. 942).
	FFT‐G and TAU family services were provided under the direction of Community Behavioural Health (CBH), a corporation contracted by the City of Philadelphia to provide mental health services for Philadelphia County Medicaid recipients.
	Participants in both groups received a wide array of community and residential services (p. 947); 20% of cases assigned to FFT‐G did not receive that service and 83% of TAU cases did not receive the alternative family therapy to which they had been assigned; 12 control cases received FFT and 1 family in the control group received FFT‐G (p. 946).
**Outcomes**	Primary outcomes were (1) youth delinquency and (2) substance use, assessed with youth self‐reports, parent reports, and official records of arrests, dispositions, and residential placements.
	Secondary outcomes were
	youth peer relationships (peer influence, gang involvement); youth and parent beliefs about the validity of rules/laws; constructive time use (constructive activities, school participation); family functioning (family processes, parenting skills); and parent behaviour including substance use.
**Notes**	Development of the FFT‐G model was funded by Office of Juvenile Justice and Delinquency Prevention in 2009–2010 ($750,000 USD).
	The outcome study was funded by the US Centers for Disease Control and Prevention, US Office of Juvenile Justice and Delinquency Prevention, and the Smith Richardson Foundation.
	In 2018, the US National Institute of Justice funded a 5‐year follow‐up ($770,657 USD).
	Services were funded by Medicaid.
	Data on service costs were missing for 2 FFT‐G cases. The average cost of all services per family was $9888 USD per FFT‐G case and $9031 USD per TAU case. This is because fewer TAU cases received any services. Some discussions of costs use the number of cases that actually received some services (64 FFT‐G cases vs. 46 TAU cases) in the denominator, but that appears to inflate the actual cost of TAU.

Risk of bias table

**Table:** 

Bias	Authors’ judgement	Support for judgement
Random sequence generation (selection bias)	Unclear risk	Families were randomly assigned ‘by the research team using a list of random numbers previously computer‐generated by the PI. Only the research manager and the PI had access to the random assignment list, and only the research manager consulted the list to carry out the random assignment’ (Gottfredson et al., 2018, p. 943).
Allocation concealment (selection bias)	Unclear risk	'Randomisation results were never communicated to field staff’ (2018, p. 943).
Baseline equivalence	High risk	Compared with control cases, fewer FFT‐G youth had spent time in a residential facility in the 6 months before intake (52% vs. 74%, *d* = 0.56); FFT‐G youth reported higher levels of delinquency, violent delinquency, and a greater variety of hard drugs used in the 6 months before intake (p. 948).
Performance bias (confounding)	High risk	Almost all (97%) of FFT‐G cases received some service, compared with 73% of control cases (*d* = 1.36). FFT‐G therapists were more willing to provide services in family homes compared with other family therapists (Gottfredson et al., 2018).
Detection bias (blinding of assessment): administrative data	Low risk	Use of administrative data on criminal justice contacts, residential placements, and service use.
Detection bias (blinding of assessors): participant reports	Unclear risk	No discussion of blinding of assessors.
Attrition bias: administrative data	Low risk	1% to 2% attrition at 18 months with 2% to 5% differential attrition.
Attrition bias: participant reports	Low risk	With the exception of one item (parents’ ratings of youth peers), attrition was 9% to 14% at 6 months, with 0% to 7% differential attrition. For parents’ ratings of peers, attrition was 32% and differential attrition was 14% (this single item was coded as high risk of bias on attrition).
Standardised observation periods	Low risk	'The number of days elapsed from randomisation to post‐test was…similar for treatment and control participants, ranging from 215 to 224 days elapsed’ (p. 944).
Intention‐to‐treat	Low risk	Data were analysed according to group assignment, regardless of whether services were received (13 of 66 FFT cases (20%) did not receive FFT; 52 of 63 (84%) cases assigned to alternative family therapy did not receive that treatment and, of these families, 12 received FFT and 1 received FFT‐G).
Selective reporting (reporting bias)	Unclear risk	There is no public protocol for this study.
Validated outcome measures	Unclear risk	Use of administrative data (low risk of bias) plus self‐report measures with some reliability coefficients above and some below 0.7 (2018, p. 945).
Conflicts of interest	Low risk	The authors declared that they had no conflicts of interest.

Hansson, 2000

**Table:** 

**Methods**	Random assignment of delinquent youth to FFT or TAU groups, after screening by a social worker at a police station in Lund, Sweden. Enrolment into the study occurred between 1993 and 1995. Consent to participate was requested after random assignment had been performed; as a result, 21 of 40 cases assigned to the TAU group refused to participate further and only 19 TAU cases participated in follow‐up interviews.
	Data on recidivism gathered (by the same social worker who conducted intake) from police and social service records at 1‐ and 2‐year follow‐ups. Participant reports on outcome measures were obtained during home visits at baseline and 2 years.
**Participants**	89 Swedish youth reported for delinquency; 49 were assigned to FFT, 40 to TAU. Mean age at intake is 15.1 years; 87% of youth are male. No information on race/ethnicity.
**Interventions**	FFT (average of 10 sessions) versus TAU (individual and family counselling).
**Outcomes**	Recidivism in delinquency based on administrative (police and social service) records at 1‐year and 2‐year follow‐ups. Self‐administered questionnaires during home visits at baseline and 2‐year follow‐up, including the CBCL, SCL‐90, KASAM (sense of coherence), and self‐reported delinquency scales.
**Notes**	Funded by Swedish Board of the Research Council.

Risk of bias table

**Table:** 

Bias	Authors’ judgement	Support for judgement
Random sequence generation (selection bias)	Unclear risk	Randomisation is mentioned, but methods are not described.
Allocation concealment (selection bias)	Unclear risk	Randomisation is mentioned, but methods are not described.
Baseline equivalence	High risk	Group differences in mean age (*d* = 0.51), with a larger portion of youth under age 14 in FFT (16% vs. 3% in TAU; *d* = 1.12) and more youth over age 16 in the TAU group (58% vs. 45% of FFT cases; *d* = 0.28).
Performance bias (confounding)	Unclear risk	No details provided on amounts or types of treatment received by the TAU group.
Detection bias (blinding of assessment): administrative data	Unclear risk	Unclear whether assessors were blind to group membership.
Detection bias (blinding of assessors): participant reports	Unclear risk	Unclear whether assessors were blind to group membership.
Attrition bias: administrative data	Low risk	No attrition in administrative data at 1‐ and 2‐year follow‐ups.
Attrition bias: participant reports	High risk	37%–55% missing data overall (Tables 5 and 6) with 27%–54% differential attrition: missing data on 30%–43% of FFT cases and 70%–83% of TAU cases.
Standardised observation periods	Low risk	Observations at 1 and 2 year after the start of treatment for both groups.
Intention‐to‐treat	Unclear risk	No attrition for outcomes based on administrative records. Some outcomes based on participant reports are presented for FFT cases only, due to substantial proportions of missing data in the TAU group.
Selective reporting (reporting bias)	High risk	There is no protocol for this study. Incomplete reporting of SCL‐90 data on numbers of symptoms; CBCL data on total burden, internalising and externalising behaviours, and social competence; and the KASAM measure (p. 8). No reporting of results on self‐reported delinquency.
Validated outcome measures	Unclear risk	Use of administrative records, two standardised measures (CBCL, SCL‐90), plus two measure with unreported data on reliability and validity.
Conflicts of interest	High risk	Kjell Hansson was the owner of FFT‐Sweden, the agency responsible for licensing FFT in that country.

Hollimon, 2004

**Table:** 

**Methods**	Random assignment (or exclusion from random assignment) was conducted by the chief juvenile probation officer in a small Midwestern, largely rural community in the USA. Data collection began in 2001.
	Pre‐treatment and Post‐treatment data were obtained from youth, parents, and probation officers. ‘Pre‐treatment’ assessments occurred during the first two meetings with the probation officer; the second ‘Pre‐treatment’ assessment could be up to 6 months after the first one (p. 84). ‘Post‐treatment’ assessments occurred at formal discharge from probation, which could be up to 3 years after referral (Hollimon, 2004; p. 58).
**Participants**	Participants were 57 youth on formal probation who were not receiving inpatient treatment or other counselling services, not in need of crisis services, and had family members available (p. 82).
	Most of the youth were White (93%), males (67%), ranging in age from 13 to 18 years. Most (64%) were enroled in school full time, 84% were living with the family they grew up in, and half of families had incomes below $35,000 (p. 92).
**Interventions**	FFT plus probation (*n* = 27) versus supervised probation (SP) only (*n* = 30). Cases excluded from random assignment received other mental health services (higher intensity) and are not included here.
	FFT was provided by counselling psychology graduate students and usually consisted of 12 sessions over 3 months. 67% of FFT cases completed this treatment (p. 94).
	FFT cases received an average of 6.3 total hours of service. Cases in the supervised probation (SP) group received an average of 7.4 total hours of service.
**Outcomes**	Youth behaviour, parent functioning, and family functioning were assessed with the OQ‐45.2; FAM‐III; POSIT, POSIP, and POSIT‐FU subscales (family relations, youth substance abuse, mental health, peer relations, aggressive behaviour, school performance, social skills); and the Washington State Juvenile Court (WSJC) prescreening tool. The WSJC was completed by probation officers. Composite scores were created from selected subscales of the POSIT, POSIP, and POSIT‐FU despite lack of norms for this in other samples and low internal consistency in the study sample (most *α*s < 0.7, pp. 187‐189).
	Post‐treatment data were obtained from youth and probation officers in 61% of the cases. Only 47% of mothers and fewer fathers provided outcome data (thus, we did not use parental reports in meta‐analysis).
**Notes**	

Risk of bias table

**Table:** 

Bias	Authors’ judgement	Support for judgement
Random sequence generation (selection bias)	Unclear risk	Adolescents were excluded from random assignment or ‘randomly assigned by the chief juvenile probation officer’ (p. 16, p. 83). Random assignment methods are not described.
Allocation concealment (selection bias)	Unclear risk	The chief juvenile probation officer had access to a unique identification code for each adolescent (p. 82). ‘Meetings were held… on a regular basis to assure fidelity to the random assignment’ (p. 82).
Baseline equivalence	High risk	There were no statistically significant differences between groups on gender, race, or age, but this could be due to the small sample size and low statistical power. For race, 4% of FFT and 10% of Supervised Probation (SP) groups were Black (d(logit) = 0.58). For age, 15 of 26 FFT cases and 19 of 27 SP cases were 16‐18 years of age (*d* = 0.31). One of 23 FFT cases and 4 of 29 SP cases were not living with family (*d* = 0.69); 14 of 20 FFT families and 16 of 27 SP families had incomes under $35,000 per year (*d* = 0.26). Four of 20 FFT cases and 3 of 30 SP cases had some family member in jail or prison (*d* = 0.32).
Performance bias (confounding)	Unclear risk	All participants were on probation; FFT was an additional service. ‘Each treatment group received different types and durations of counselling services, educational services, case management services, and other community services’ (p. 93), but cases in SP group received a more hours of service on average than the FFT group.
Detection bias (blinding of assessment): administrative data	Unclear risk	
Detection bias (blinding of assessors): participant reports	High risk	Data provided by probation officers, parents, and youth were collected during their meetings together (p. 84).
Attrition bias: administrative data	Unclear risk	
Attrition bias: participant reports	High risk	39% attrition on outcomes reported by youth (p. 208), 53% attrition on outcomes reported by mothers (p. 209), 88% attrition on outcomes reported by fathers (p. 210).
Standardised observation periods	High risk	Post‐treatment assessments took place at discharge from probation, which could be up to 3 years after referral (p. 58).
Intention‐to‐treat	Unclear risk	Unclear due to high levels of missing data. Families who dropped out of FFT were included in the analysis (p. 93).
Selective reporting (reporting bias)	High risk	A protocol for this study is not available. Data were collected on POSIT, POSIP, and POSIT‐FU subscales on family relations, youth substance use, health, mental health, peer relations, social skills, and aggressive behaviour/delinquency; results were reported for the family relations subscale and a composite score based on other scales.
Validated outcome measures	Unclear risk	Original outcome measures had acceptable levels of internal consistency in similar samples, and a risk assessment instrument (WSJC) was associated with recidivism in similar samples. Selected subscales were combined despite lack of norms for this in other samples and low internal consistency in the study sample (most *α*s < 0.7, pp. 187–189).
Conflicts of interest	High risk	The author was one of the FFT therapists in the study and she was supervised by two of the FFT developers (Thomas Sexton and James Alexander).

Humayun, 2010

**Table:** 

**Methods**	Young people (*n* = 111) with antisocial or criminal behaviour were randomly assigned to FFT versus regular casework in two counties in England between 2008 and 2011. Two‐thirds (67%) of these youth were recruited for the study through Youth Offending Services (YOS), 22% through Targeted Youth Support Services (a multiagency prevention service for antisocial youth), and 11% through other crime prevention agencies.
	Data were collected via structured interviews and observations with youth and their primary caregivers at baseline, 3, 6, and 18 months after random assignment. Archival data on criminal offenses were obtained from the YOS and Police National Databases (PND).
**Participants**	Youth ages 10–17 (mean age = 15.0 years) were predominantly White British (90%), with below average IQ (mean = 84). Most lived with single (55%), unemployed (57%) carers, 85% of whom were the youth's biological mother; 60% of carers had no education after the age of 16 (Humayun et al., 2017).
**Interventions**	All cases received case management as usual (MAU), as required by law.
	FFT consisted of approximately 12 sessions over 3–6 months and was structured in five phases (engagement, motivation, assessment, behaviour change, generalisation). FFT was provided by Master's level Systemic Family Psychotherapists, who had up to 10 years of experience working with families and youth with multiple problems. ‘FFT LLC provided initial training then twice weekly supervision by phone, plus by six in‐person training visits which included DVD review of therapy and live supervision. To further ensure fidelity, the FFT consultant monitored therapists’ routinely completed clinical session notes’ (Humayun et al., 2017, p. 1025).
	In addition to MAU, the ‘dose control group’ was offered additional casework services (up to 12 h of support and counselling provided by a caseworker who was not trained in FFT) in attempt to match the amount of time and attention received by participants in FFT.
	Service use data showed that FFT cases received more MAU services than MAU cases (18 vs. 11 h) and more hours of service overall (28 vs. 11 h; Humayun et al., 2017).
**Outcomes**	The protocol (2010) described two sets of primary outcomes:
	1. Offending, reoffending, and breach of orders, assessed with data from the YOS and PND. (Changed to secondary outcomes in the 2017 report.) 2. Delinquency, conduct disorder, and antisocial behaviour, assessed with:
	Self Reported Delinquency (SRD), Adolescent Parent's Account of Child Symptoms (APACS), Strength and Difficulties Questionnaire (SDQ; parent and youth reports), and Antisocial Process Screening Device (APSD, parent and youth reports).
	Secondary outcomes included:
	Peer relationships: peer delinquency, relationship problems, and prosocial behaviour (SRD, SDQ); Young people's mental health: internalising disorders (SDQ), hyperactivity/inattention (Conner's Rating Scale for ADHD); Young people's education attainment (school attendance, exclusions, academic achievement, and statements of special educational needs from school records); Family relationships and parenting: CAI, IMS, CTS, CHOS, APQ, Parental attribution of intent (see protocol), Carer Confidence Questionnaire; Parent Development Interview; Psychosocial Assessment of Childhood Experiences Coding Scheme; video observations of youth and carer coded with Problem Solving & Dyad Interaction Coding Scheme; Parental mental health: Depression, Anxiety and Stress Scales; Antisocial Personality Questionnaire; Alcohol and Drug Use Disorders Identification Tests; Service use: Client Service Receipt Inventory (parent interview); Quantity and type of treatment as usual received by young people (YOS and other agency databases).
**Notes**	Funding (including salary support) was provided by the UK National Institute for Health Research (NIHR) Biomedical Research Centre for Mental Health at the South London and Maudsley NHS Foundation Trust, King's College London, and the UK Department for Education.
	Most primary outcome measures were reported; most secondary outcomes were not reported.

Risk of bias table

**Table:** 

Bias	Authors’ judgement	Support for judgement
Random sequence generation (selection bias)	Low risk	'Families were randomised to FFT or control group by a statistician independent from the research team using a random number generator employing constrained adaptive randomisation. The randomisation ratio was varied to ensure adequate caseloads for FFT therapists and varied from 3:1 cases (FFT:control) during the early period to 1:3 cases at the end’ (2017, p. 1025)
Allocation concealment (selection bias)	Low risk	Random assignment was conducted by ‘a statistician independent from the research team’.
Baseline equivalence	High risk	At baseline, FFT youth had higher self‐reported delinquency (SRD) scores (*d* = 0.26) and a larger proportion of FFT cases were identified with early onset conduct disorder, compared with comparisons cases (55% vs. 41%, *d* = 0.31).
Performance bias (confounding)	High risk	In attempt to match the time and attention provided by FFT, cases in the control group were offered an additional 12 h of casework, but it appears that these services were not used. Service utilisation data showed that FFT cases received more TAU services than TAU cases (18 vs. 11 h) and more hours of service overall (28 vs. 11 h).
Detection bias (blinding of assessment): administrative data	Unclear risk	No discussion of blinding of assessors.
Detection bias (blinding of assessors): participant reports	Unclear risk	No discussion of blinding of assessors.
Attrition bias: administrative data	Low risk	Approximately 20% missing data at 6 and 18 months, with 1% differential attrition.
Attrition bias: participant reports	Unclear risk	For SRD, 19% missing at 6 months, 21% missing at 18 months (low risk); for observational data 35% missing at 6 months, 41% missing at 18 months (high risk; 2017, p. 1029).
Standardised observation periods	Unclear risk	No discussion of variations in observation periods.
Intention‐to‐treat	Low risk	Analysis included FFT cases that did not accept, engage in, or complete FFT treatment (2017, p. 1027)
Selective reporting (reporting bias)	High risk	The protocol for this study was retrospectively registered (the trial started on 1 July 2008 and was registered on 13 May 2010). One of the two primary outcomes listed in the 2008 protocol was described as a secondary outcome in the final report (2017). Results on two measures of primary outcomes (SDQ, APSD) were not reported; most secondary outcomes were not reported.
Validated outcome measures	Low risk	Investigators relied on administrative data and measurement instruments/protocols (including observational measures) with reliability coefficients > 0.7.
Conflicts of interest	Low risk	Authors declared they had no competing or potential conflicts of interest.

Ogden, 2013

**Table:** 

**Methods**	Between 2013 and 2017, 159 youth and their families were randomly assigned to FFT or treatment as usual (TAU) for youth with behavioural problems in three sites (Skien, Trondheim, and Stavanger) in Norway.
	Outcome data were collected by staff of the Norwegian Center for Child Behavioural Development (NCCBD), either in family homes or at service provider offices, at 6 and 18 months after treatment began. Questionnaires were computerised and participants responded on laptop computers (Thøgersen, 2021).
**Participants**	Families of youth (ages 11–19) with behavioural problems (e.g., aggression, violence, rule breaking, school behaviour problems, drug abuse, and/or criminal history). Initial eligibility criteria were relaxed to include low risk cases (Thøgersen, 2021). More than half (54%) of youth were males, with an average age of 14.7 years.
**Interventions**	FFT was provided in three sites, and structured in 5 phases (engagement, motivation, assessment, behaviour change, and generalisation). No information is available on staff qualifications.
	The control group could receive any available child welfare, child mental health, or family counselling service (Familievernet) other than FFT; many control cases received Multisystemic Therapy (Thøgersen, 2021).
	No information is available on the frequency, amount, or duration of services provided.
**Outcomes**	Primary outcomes included:
	1) parents’ and teachers’ reports on
	the CBCL (externalising, aggression, rule‐breaking, attention, internalising, and total scales); the SNAP‐4 Rating scale (inattention, hyperactivity/impulsivity, and ODD); the Connors Index Questionnaire (a general index of childhood problems); the Social Skills Rating System (SSRS; assessing cooperation, assertion, responsibility, and self‐control);
	2) Youth self‐reports on the Self‐Reported Delinquency scale (SRD); 3) Archival data on arrest/recidivism (after 18 months and 36 months); and 4) Placement outside the home (based on parents’ report on where the youth was living at the time of the assessment and during most of the previous 12 months).
	Authors provided data on the first two sets of outcomes, but not on arrest/recidivism or out of home placement.
	Secondary outcomes included measures of:
	parental mental distress (Norwegian version of the SCL‐8); family functioning (using the Family Environment Scale (FES) cohesion and conflict scales, and a Norwegian version of the Conflict Tactics Scales, CTS); Inventory of Callous‐Unemotional Traits (ICU; used to measure emotional dysregulation); parents’ and youths’ alcohol consumption (Alcohol Use Disorders Identification Test, AUDIT); parenting behaviour (Alabama Parenting Questionnaire (APQ) scales on positive reinforcement, parental involvement, inconsistent discipline, poor monitoring and supervision, and harsh discipline); The Unidimensional Relationship Closeness Scale; The Inclusion of Other in the Self scale; The Influence in Families Questionnaire (IFQ); The Interpersonal Sense Of Control measure (ISOC); Client Outcome Measure (COM; client‐perceived changes); Inventory of Parent and Peer Attachment (IPPA) WHO Wellbeing index; Norwegian version of the Child and Adolescent Social Support Scale (CASSS); The Bergen Questionnaire on Antisocial Behaviour; The Gang Membership Questionnaire; the Oregon Healthy Teens Survey (OHS); the Personal Experiences Inventory (PEI); and the Angry Aggression scales (four types of violence).
	Data on secondary outcomes were not available.
**Notes**	The protocol for this study (ISRCTN58861782) was registered retrospectively (1 month after randomisation began). Funding was provided by the Norwegian Center for Child Behavioural Development (NCCBD). Results are not publicly available, but data on most primary outcomes were provided by investigators.

Risk of bias table

**Table:** 

Bias	Authors’ judgement	Support for judgement
Random sequence generation (selection bias)	Unclear risk	Random allocation sequences were generated by staff at NCCBD, using random.org, with a separate sequence for each site (Thøgersen, 2021).
Allocation concealment (selection bias)	Unclear risk	Allocation sequences were known only to staff tasked with randomisation (Thøgersen, 2021).
Baseline equivalence	High risk	At baseline, teachers reported that FFT cases had more behavioural problems (*d* = 0.25) and more problems with inattention (*d* = 0.28) than youth in the control group; teachers viewed FFT youth as less cooperative (*d* = 0.36), and parents viewed them as less responsible (*d* = 0.27) than control cases.
Performance bias (confounding)	Unclear risk	No information on amounts and duration of services.
Detection bias (blinding of assessment): administrative data	Unclear risk	
Detection bias (blinding of assessors): participant reports	High risk	Data was collected by NCCBD staff, who were aware of treatment assignments after the pre‐test (Thøgersen, 2021).
Attrition bias: administrative data	Unclear risk	
Attrition bias: participant reports	High risk	On the 6 and 18 month assessments, 31% to 42% of all cases were missing data on parent or youth reports; more than half of all cases were missing teachers’ reports (thus, the latter were not used in our analyses).
Standardised observation periods	Unclear risk	No information on variations in observation periods.
Intention‐to‐treat	Unclear risk	All FFT cases started treatment.
Selective reporting (reporting bias)	High risk	The protocol was registered (retrospectively) on 24 May 2013. Random assignment began on 22 April 2013 (Thøgersen, 2021). Data on some primary outcomes were provided by authors; data on secondary outcomes were not available. There is no public report on this study.
Validated outcome measures	Unclear risk	Use of standardised instruments (e.g., CBCL) translated into Norwegian with good reliability data (from other samples) on some (not all) of these instruments.
Conflicts of interest	Unclear risk	No public conflict of interest statement.

Ozechowski 2012

**Table:** 

**Methods**	Beginning in 2012, in two rural counties (Sandoval and Valencia) in New Mexico (USA) adolescents who met diagnostic criteria for substance abuse or dependence were randomly assigned to FFT, FFT via video conferencing, or treatment as usual (TAU).
	Assessments were conducted at baseline, 4, 8, and 12 months after random assignment.
**Participants**	90 youth, ages 13–18, were referred by juvenile probation officers.
	Youth and their families lived in rural communities located 30 to 50 miles from the Center for Family and Adolescent Research (CFAR) in Albuquerque, NM.
**Interventions**	**FFT** administered face‐to‐face in the family home in 12 to 14 weekly family sessions. Structured in 5 phases. (Not provided with laptop and Internet access described below.)
	**FFT‐V**: FFT administered by video teleconference in the homes of substance abusing adolescents. Participants ‘receive a Verizon netbook laptop computer equipped with the Microsoft Windows 7 operating system, webcam, and VTC software for use in the family home. The VTC software is designed to stream live video between therapists and participants, to record video, and to store recorded videos as digital mpeg files. All family sessions will take place via video teleconference.’
	**TAU** coordinated through the Juvenile Services Division (JSD) of the state Children, Youth, and Families Department (CYFD). Services are provided by Youth Development Incorporated (YDI), a private non‐profit organisation, which ‘provides an array of services for youth including tutoring, after‐school activities, gang intervention, school drop‐out prevention, family counselling services, an emergency teen shelter, parenting skills training, youth leadership development, community corrections services, GED studies, substance abuse and AIDS education, etc. The YDI juvenile corrections services include intensive supervision, educational and employment assistance, community service, victim restitution, and institutional transition services.'
**Outcomes**	Timeline Followback semi‐structured interview (TLFB) used ‘to determine the percent days of substance use excluding tobacco, as well as abstinence days and binge drinking days.’
	Urine Assays (NIDA 9 Test Panel) to screen for amphetamine, barbiturates, benzodiazepines, cocaine, metamphetamine, methadone, morphine, PCP, and THC (marijuana).
	Other outcome measures were not specified. ‘Outcome analyses will examine adolescent substance abuse, HIV risk behaviour, delinquency, and recidivism, family functioning, and adolescent association with substance abusing peers’ (https://clinicaltrials.gov/ct2/show/NCT01751217)
**Notes**	Funded by US NIDA project DA032260, with grants of $2,519,220 over four years (2012‐2016) to Dr. Timothy J. Ozechowski and Dr. Holly B. Waldron at the Oregon Research Institute. This study was completed in 2016; there are no public reports of outcomes, and authors did not respond to repeated requests for information on this study.

Risk of bias table

**Table:** 

Bias	Authors’ judgement	Support for judgement
Random sequence generation (selection bias)	Unclear risk	No information available.
Allocation concealment (selection bias)	Unclear risk	No information available.
Baseline equivalence	Unclear risk	No information available.
Performance bias (confounding)	Unclear risk	No information available.
Detection bias (blinding of assessment): administrative data	Unclear risk	No information available.
Detection bias (blinding of assessors): participant reports	Unclear risk	No information available.
Attrition bias: administrative data	Unclear risk	No information available.
Attrition bias: participant reports	Unclear risk	No information available.
Standardised observation periods	Unclear risk	No information available.
Intention‐to‐treat	Unclear risk	No information available.
Selective reporting (reporting bias)	High risk	The protocol for this study was registered in December 2012 (retrospectively); the study began in February 2012, was completed in 2016. Outcome data were not reported.
Validated outcome measures	Unclear risk	No information available.
Conflicts of interest	High risk	Dr. Holly B. Waldron is CEO of LIFFT, Co. (Leading Implementations in Functional Family Therapy), an organisation that disseminates FFT into community settings. Dr. Timothy Ozechowski is Director of Research for LIFFT.

Regas, 1983

**Table:** 

**Methods**	In Tippecanoe County, Indiana (USA), 20 ‘hyperactive’ adolescents were grouped into male‐female pairs and pairs were randomly assigned to one of three groups: FFT, group therapy, and a no treatment control group. Pre‐treatment and Post‐treatment (8 week) assessments were conducted for all cases; then families in the no treatment control group received the treatment of their choice. Cases in the FFT and group therapy conditions were assessed 5 months after the post‐test (approximately 7 months after random assignment). Two cases were lost after the pre‐test, leaving 6 cases in each group. The study was conducted between 1981 and 1983.
	Data were collected by the investigator at before treatment, after treatment (8 weeks) and 5 months later. Outcomes were derived from data reported by from youth, parents, and teachers.
**Participants**	Participants were families of 20 eighth and ninth graders, who had been classified as ‘hyperactive,’ based on ratings from two teachers. More than half (56%) were male, with an average age of 13.8 years. Thirty‐two parents (18 mothers, 14 fathers) and 16 siblings participated.
**Interventions**	Treatments were provided at Perdue University's Marriage and Family Center and lasted 8 weeks.
	**FFT** was provided by 3 female and 1 male therapist, who were doctoral or Master's students in Purdue's Marriage and Family Therapy program. They had 1‐4 years of prior experience with family therapy, received 10 h of pre‐service didactic training in FFT, and received live supervision from the investigator for 30 min before, during, and 30 min after each family session. Live sessions were sometimes interrupted and redirected by the supervisor (Regas, 1983; p. 67).
	**Group therapy** was co‐lead by one male and one female graduate student in Purdue's Master's program Family Studies. Groups were comprised of youth only, but family members participated in assessments. Therapists received 3 h of pre‐service didactic training and supervision from the investigator for 60 min before and 60 min after each group session. Group sessions were not interrupted.
	The **no treatment control** condition included an option to request therapy on a one‐time basis if needed, but none of the cases requested this. Three (half) of the families each had one 30 min phone consultation with the investigator during the no treatment phase (p. 41).
**Outcomes**	Data on family functioning were gathered on the Family Concept Inventory (FCI, 12 subscales). Classroom behaviours were assessed with the Connors Teacher Rating Scale (CTRS, 5 subscales). Parents’ views of youth hyperactivity, aggressiveness, and ‘inappropriate behaviour’ were assessed with the Werry‐Weiss‐Peters Activity Scale (WWPAS) and Parent Checklist of Child's Behaviour (PCCB). The Tennessee Self Concept Scale (TSCS) was used to assess youth self‐esteem.
	No information was available on the reliability or validity of the PCCB; therefore, data collected on this instrument were not included in our analyses.
	The Kvebaek Family Sculpture Test was used to assess perceived distance between family members. This was not included in our analyses, because it did not tap one of our primary or secondary outcomes.
**Notes**	Groups were small (*n* = 6 in each of 3 groups) and there were between‐group differences in family history (number of marriages and divorces) and income.

Risk of bias table

**Table:** 

Bias	Authors’ judgement	Support for judgement
Random sequence generation (selection bias)	Unclear risk	Sequence generation method was not described. It is not clear how random assignment was conducted or by whom.
Allocation concealment (selection bias)	Unclear risk	Details of random assignment were not provided. Allocation concealment was not discussed.
Baseline equivalence	High risk	On average, families in the comparison and control groups had more marriages and more divorces than those in the FFT group; there were also significant between‐group differences on income, but these were not explained (Regas, 1983; p. 53).
Performance bias (confounding)	High risk	The investigator provided ‘live supervision’ during the FFT sessions, watching sessions behind a one‐way mirror and conferring with the therapist before the end of each session to formulate homework assignments for the family.
Detection bias (blinding of assessment): administrative data	Unclear risk	
Detection bias (blinding of assessors): participant reports	High risk	All measures were administered by the investigator, who also provided therapist training and supervision in FFT and group therapy cases.
Attrition bias: administrative data	Unclear risk	
Attrition bias: participant reports	High risk	10% attrition overall; differential attrition > 10% (0% attrition in FFT, 14% in group therapy, 14% in no treatment control group). After attrition, males comprised 4/6 of FFT cases, 3/6 of group therapy, and 3/6 of no‐treatment control cases; and, on average, FFT cases were one year older than cases in the other two groups (Regas, 1983, p. 46).
Standardised observation periods	Low risk	Assessments were all conducted within 2 week windows.
Intention‐to‐treat	High risk	Loss of 2 of 20 cases (1 in each comparison group) after random assignment and pre‐test.
Selective reporting (reporting bias)	Unclear risk	There is no public protocol for this study.
Validated outcome measures	Unclear risk	Use of assessment instruments with inter‐rater reliability coefficients above and below 0.7.
Conflicts of interest	High risk	No conflict of interest statement was provided. The investigator trained and supervised therapists, and was involved in formulating homework assignments for families in every FFT session.

Robbins, 2012

**Table:** 

**Methods**	Beginning in 2012, at the Center for Family and Adolescent Research in Albuquerque, NM (USA), youth with substance abuse problems were randomly assigned to four groups: (1) FFT, (2) Motivational Enhancement Therapy/Cognitive Behavioural Therapy (MET/CBT) groups, (3) FFT + Contingency Management (CM), and (4) MET/CBT + CM. (Only the comparisons between the FFT (*n* = 32) and MET/CBT (n = 27) groups are relevant for our review.)
	According to the protocol for this study, the primary outcomes was drug abuse abstinence; secondary outcomes were adolescent sexually risky behaviour and conduct problems; exploratory outcomes were drug avoidance self‐efficacy and family functioning. Drug and alcohol use was assessed with weekly urine and saliva screens and parent and youth reports (2020); other outcomes were assessed at baseline, 2, 4, 8, and 12 months after randomisation.
**Participants**	Participants were youth, ages 13‐18 (mean age=15.9 years), who met diagnostic criteria for substance abuse or dependence, and were living at home with one or more parents. Most (76%) were male; most (77%) were Hispanic, with 14% White non‐Hispanic, 7% Native American, 2% Black, and 1% Asian American. Median family income was $33,344.
	Baseline urine drug tests for substance use were positive for 77% of youth (2020, p. 6).
**Interventions**	Both treatments lasted approximately 14 weeks; mean number of sessions attended = 13.
	FFT involved weekly family sessions, structured in five phases (engagement, motivation, relational assessment, behaviour change, and generalisation).
	MET/CBT groups included family members in first session (only). Remaining sessions focused on decision‐balance (motivation), skills training, coping with cravings, assertive communication, drug refusal, managing negative moods, problem‐solving, decision‐making, and relapse prevention.
**Outcomes**	Urine Assays (NIDA 12 Test Panel) were used to screen for the presence of amphetamine, barbiturates, benzodiazepines, cocaine, methamphetamine, methadone, morphine, Oxycodone, PCP, Propoxyphene, Opiates, and THC (marijuana).
	Alcohol use was measured with Saliva Screens (Alco‐Screen 02), ‘a simple, non‐invasive, one‐step test that provides results in four minutes and detects alcohol levels of 0.02 or greater’ (2020, p. 12).
	The TimeLine FollowBack semi‐structured interview (TLFB) was used to determine the percent days of substance use (primary measure), excluding tobacco, as well as abstinence days and binge drinking days.
	Drug avoidance self‐efficacy assessment was planned but not reported.
	Assessment of adolescents’ risky sexual behaviour was planned but not reported.
	Conduct problems were assessed with parent and youth reports on CBCL/YSR; total scores were used to measure youth reports of externalising behaviour (internalising scores were not reported). Researchers reported within‐group reductions in YSR scores below clinical thresholds (*X* < 19) from baseline to month 4 and baseline to month 12; between‐group comparisons were not fully reported, but differences between FFT and MET/CBT appeared to be non‐significant.
	Family relationships were assessed with the FES; only the conflicts subscale was reported; effects of treatment modality were not significant and not fully reported.
**Notes**	Funded by US NIH NIDA grants (project DA 032723) of $2,991,811 over five years (FY 2012‐2016) to Dr. Michael S. Robbins at the Oregon Research Institute.

Risk of bias table

**Table:** 

Bias	Authors’ judgement	Support for judgement
Random sequence generation (selection bias)	Unclear risk	No discussion of sequence generation methods.
Allocation concealment (selection bias)	Unclear risk	No discussion of allocation concealment.
Baseline equivalence	High risk	Caregivers in the FFT group were more likely to have completed high school than those in the MET/CBT group (77% vs. 59%, *d* = 0.49). Unclear whether there were baseline differences between youth in FFT vs. MET/CBT.
Performance bias (confounding)	Low risk	Similar number of sessions and duration of treatment in both groups.
Detection bias (blinding of assessment): administrative data	Unclear risk	
Detection bias (blinding of assessors): participant reports	Unclear risk	No discussion of blinding of assessments.
Attrition bias: administrative data	Unclear risk	
Attrition bias: participant reports	Low risk	12% attrition with 5% differential attrition at 4 months; 20% attrition with 3% differential attrition at 12 months.
Standardised observation periods	Low risk	Data collected at baseline, 2, 4, 8, and 12 months, plus weekly biologic tests for drug and alcohol use.
Intention‐to‐treat	Unclear risk	No discussion of intention to treat analysis.
Selective reporting (reporting bias)	High risk	The protocol for this study was registered retrospectively (on 29 November 2012; the study began in July 2012). Outcomes were not fully reported (e.g., multiple measures of substance use, CBCL/YSR, FES). Some non‐significant differences were not fully reported (e.g., effects of treatment modality (FFT vs. MET/CBT) on family conflict).
Validated outcome measures	Low risk	Use of biologic measures and standardised scales with *α*s > 0.7.
Conflicts of interest	High risk	'Dr. Robbins receives compensation from FFT LLC, an organisation that disseminates FFT into community settings. Dr. Waldron receives compensation from LIFFT, Co., an organisation that disseminates FFT into community settings’ (Robbins et al., 2020; p. 1).

Slesnick & Prestopnik, 2004

**Table:** 

**Methods**	Runaway adolescents with alcohol problem (*N* = 119) were identified in two runaway shelters in Albuquerque, New Mexico (USA) before 2004. Eligible youth were randomly assigned to (1) FFT, (2) an ecologically‐based family therapy (EBFT), or (3) treatment as usual (TAU probation).
	Youth and family functioning outcomes were assessed via reports from youth and their primary caregiver at baseline, 3, 9, and 15 months. Urine toxicology screens were assessed at baseline and 3 months.
**Participants**	Youth age 12–17 (mean age = 15.1 years) who lived within 60 miles of the research site in Albuquerque, NM; 45% were male; 29% White, 5% Black, 44% Hispanic.
**Interventions**	Two active treatments were provided by the same therapists, who were trained and supervised by the lead investigator. Both treatments were offered as 16 1‐h sessions over a 3–6‐month period.
	FFT services were office‐based and included no sessions with individual family members.
	Ecologically‐Based Family Therapy (EBFT) was modelled on the Homebuilders family preservation program.
	TAU was provided through the runaway shelters and consisted of case management and more.
**Outcomes**	Outcomes were based on structured interviews with youth and parents at baseline, 3, 8, and 15 months. Due to missing data from parents, outcome analyses relied solely on youth self‐reports.
	Youth substance use was assessed with (1) Form 90 (which combines the timeline follow‐back method (TFBM) with grid averaging), (2) POSIT, (3) the Adolescent Drinking Inventory (ADI), and (4) urine toxicology screens (at baseline and 3 months only).
	Youth symptoms were assessed with the (1) CBCL YSR (internalising and externalising scales), (2) the BDI, and (3) CDISC sections on sections on Conduct Disorder, Oppositional Defiant Disorder, Mood, Eating and Anxiety disorders.
	Data on youth delinquency related on the National Youth Survey Delinquency Scale (NYSDS) and its scores for general theft, crimes against persons, index offenses, drug sales and total delinquency.
	Family and parent functioning were assessed with (1) the Family Environment Scale (FES), (2) he Conflict Tactic Scale (CTS), and (3) the Parental Bonding Inventory (PBI).
**Notes**	Funded by a USA NIAAA and CSAT grant (R01 AA 12173).
	Outcome analyses were restricted to the subgroup of youth who completed all 4 assessments (*n* = 75/119).

Risk of bias table

**Table:** 

Bias	Authors’ judgement	Support for judgement
Random sequence generation (selection bias)	Low risk	Computer‐adjusted (urn) randomisation was used in attempt to balance group composition by gender, age, ethnicity, number of days of substance use in the last 3 months, comorbidity status, and number of previous runaway episodes.
Allocation concealment (selection bias)	Unclear risk	No discussion of allocation concealment.
Baseline equivalence	High risk	Youth in the FFT group had more prior arrests (mean = 4.39, SD = 9.18) than those in the the other two groups (mean = 2.76, pooled SD = 3.98, *d* = 0.26). Youth in the TAU group had higher delinquency scores at baseline (mean = 934, SD = 1739 vs. mean = 182, SD = 289; *d* = 0.71) and were less likely to be enroled in school than those in the active treatment groups (36% vs. 57% enroled, *χ* ^2^ = 4.99, *p* = 0.025, *df* = 1).
Performance bias (confounding)	Unclear risk	Groups received different amounts of treatment.
Detection bias (blinding of assessment): administrative data	Unclear risk	
Detection bias (blinding of assessors): participant reports	Unclear risk	No discussion of blinding.
Attrition bias: administrative data	Unclear risk	
Attrition bias: participant reports	High risk	Outcome analysis were conducted for complete cases only (*n* = 75/119); missing data on 37% of cases. Differential attrition = 3%.
Standardised observation periods	Unclear risk	Assessments were conducted at 0, 3, 9, and 15 months after baseline. No information on variability in the timing of these assessments.
Intention‐to‐treat	High risk	'Intent to treat’ analysis (tests for between‐group differences on outcomes) were based on the subsample of participants who completed all 4 assessments (75/119 cases, or 63%).
Selective reporting (reporting bias)	Unclear risk	No publicly available protocol.
Validated outcome measures	Unclear risk	Use of outcome measurement instruments with internal consistency both above and below 0.7.
Conflicts of interest	Unclear risk	No conflict of interest statement. Investigator trained and supervised therapists in both active treatment conditions.

Waldron 2001

**Table:** 

**Methods**	In Albuquerque, New Mexico (USA), 127 youth who met DSM IV criteria for substance use disorders were randomly assigned to one of four conditions, using a computerised ‘urn’ procedure in attempt to balance group composition on demographic characteristics.
	Outcome data were collected from participants at 4, 7, and 18 months after random assignment (18 month results are not available).
	Thirteen families did not complete post‐treatment assessments and were dropped from the study.
**Participants**	Participants were referred to the University of New Mexico Center for Family and Adolescent Research for drug‐abuse treatment. Referrals came from the juvenile justice system (43%), public school system (31%), self or parent referral (21%), and other treatment agencies (5%). Most youth were under a court order, probation order, or threat of school suspension for substance abuse.
	Participants included youth, ages 13‐18 (mean age 15.6); 80% were male; 47% Hispanic, 38% non‐Hispanic White; 45% single parent families.
**Interventions**	Three treatment conditions were designed to provide 12 h of service over 3 or 4 months, with a fourth (joint) treatment providing 24 h of service in the same time frame.
	**FFT** sessions were scheduled for one hour per week (actual service use was a mean of 9.7 h over an average of 15.4 weeks). FFT was applied in two phases: The first phase focused on engaging families and enhancing motivation for change. The second phase focused on creating behavioural changes in the family, using contingency management, communication and problem solving, behavioural contracting, and other behavioural interventions.
	Individual **cognitive behavioural therapy (CBT)** was structured as 12 h‐long sessions: the first 2 sessions focused on motivational enhancement and 10 sessions focused on skills in communication, problem solving, peer refusal, negative mood management, social support, work‐ and school‐related skills, and relapse prevention. CBT treatment plans were flexible, based on individual needs. Participants received an average of 9.5 h of CBT over about 16.3 weeks.
	Joint **CBT plus FFT** included both of the interventions described above, with the youth participating in individual CBT sessions and the family participating in FFT, for a total of 2 sessions per week and an average of 19.3 h of service over 18.1 weeks.
	A **psychoeducational group** for youth met in eight 90‐min sessions. Group facilitators ‘provided information about drugs and alcohol, explored expectancies and consequences of substance use, provided opportunities for adolescents to identify self‐esteem‐enhancing alternatives to substance abuse, and included some skills‐based training, such as assertiveness training and refusal skills… [The group was] highly structured, focusing on group participation and cohesion and sharing of experiences…’ (Barrett et al., 2001, p. 806). Group participants received an average of 8.4 h of service over 7.7 weeks.
	Most (7) therapists were master's‐level graduate students in clinical or counselling psychology; two held doctorates in clinical psychology.
	The contrasts of interest for our review were: FFT vs. CBT and FFT vs. group treatment.
**Outcomes**	Frequency of marijuana use was the primary outcome. Drug use was assessed with the Timeline Follow Back Interview (TFBI) form 90D, a structured self‐report. Collateral TFBI reports were obtained from parents and siblings. Urine samples were screened for a variety of drugs and alcohol. The Problem Oriented Screening InstrumenT (POSIT) was also used to assess adolescent functioning related to substance use.
	The CBCL Youth Self Report (YSR) was administered to assess internalising and externalising problems. The CBCL delinquent behaviour subscale was used. Adolescent depression was measured with the Beck Depression Inventory (BDI). Measures of perceived family conflict were included, but not described.
	We did not analyse data on caregiver substance use.
**Notes**	Funding was provided by the US National Institute on Alcohol Abuse and Alcoholism (Grant R01 AA13167) and US National Institute on Drug Abuse (Grants R01 DA11506, R01 DA18645, K01 DA139682, and R01 DA09422). Five years of funding under RO1 DA09422 = $1,217,971 USD.
	Using the statcheck program, Nuijten et al. (2015) detected 10 potentially incorrect statistical results in Barrett et al. (2001) (see https://pubpeer.com/publications/C35940A470D137C5EDF93313DF41D5#1). These appeared to be rounding errors which lowered p‐values.
	Barrett et al. (2001) and French et al. (2008) reported different baseline characteristics and different results for marijuana use at 4 and 7 months.
	Cost analysis showed that FFT cost $7061 USD per year per client, compared with $5554 for CBT and $6150 for group therapy (French et al., 2008; p. 277).

Risk of bias table

**Table:** 

Bias	Authors’ judgement	Support for judgement
Random sequence generation (selection bias)	Low risk	Computerised urn randomisation used relative probabilities of assignment to treatment groups (urns) adjusted based on previous randomisations to maximise equivalence on gender, age, substance use, ethnicity, psychiatric severity, and family composition.
Allocation concealment (selection bias)	Unclear risk	No information was provided on allocation concealment.
Baseline equivalence	High risk	Baseline comparisons provided by Barrett et al. (2001, pp. 804–805) and French et al. (2008, p. 276) were inconsistent. French et al. reported significant differences on age of first drug use and proportion of single parent families. Using data from Barrett et al. (2001), the FFT group included more two‐parent families than CBT (20/30 vs. 15/31, *d*(logit) = 0.42) and group therapy (20/30 vs. 16/30, *d*(logit) = 0.31).
Performance bias (confounding)	Unclear risk	Participants received different amounts of treatment.
Detection bias (blinding of assessment): administrative data	Unclear risk	
Detection bias (blinding of assessors): participant reports	Unclear risk	No discussion of blinding of assessors.
Attrition bias: administrative data	Unclear risk	
Attrition bias: participant reports	Unclear risk	10% missing overall (13/127); insufficient information to calculate differential attrition.
Standardised observation periods	Unclear risk	Assessments at pre‐treatment, 4 months (post‐treatment), 7 months, and 18 months. No information on variations in the timing of assessments.
Intention‐to‐treat	High risk	Thirteen families did not complete post‐treatment assessments and were dropped from analysis.
Selective reporting (reporting bias)	High risk	There is no public protocol. Null results of tests for between‐group differences (on urinalysis, CBCL, and family conflict measures) were mentioned, but details were not reported.
Validated outcome measures	Low risk	*κ*s and *α*s > 0.7.
Conflicts of interest	High risk	Dr. Holly B. Waldron is a developer of FFT for youth with substance abusing and related problems, and CEO of LIFFT (Leading Implementations in Functional Family Therapy) ‘an organization designed to train therapists and expand the reach and adoption of the FFT model’ (https://about.me/hollybarrettwaldron).

Waldron, 2005

**Table:** 

**Methods**	This ‘trial replicated the procedure in the earlier study [Waldron, 2001], but targeted 117 adolescents (92 boys, 25 girls), aged 13–19 (*M* = 16.3; SD = 1.3), who had an alcohol use disorder. In this study, percent days of baseline substance use during the previous three months was 46.87 for marijuana, 17.65 for alcohol, 61.18 for tobacco, and 54.50 for percent days on which any substance (excluding tobacco) was used….[The] Barrett et al. (2001) study provides a more detailed description of the recruitment procedures and inclusion/exclusion criteria used in both trials.’ (Waldron, 2008, p. 1778).
	This trial also appears in Waldron and Turner (2008, p. 245), with *n* = 140.
**Participants**	79% male; 45% Hispanic, 45% White non‐Hispanic (Waldron & Turner, 2008, p. 245).
**Interventions**	Same as Waldron (2001), except that all interventions were 14 sessions in length (Waldron et al., 2005, p. 1779).
**Outcomes**	Same as Waldron (2001), data collected at approximately 4, 8, and 18 months (Waldron et al., 2005; p. 1779).
**Notes**	Outcomes were presented at a conference (Waldron et al., 2005) and included in Waldron and Turner (2008) meta‐analysis. There are no published (or public) reports on these outcomes. Dr. Waldron did not respond to requests for information on this study.

Risk of bias table

**Table:** 

Bias	Authors’ judgement	Support for judgement
Random sequence generation (selection bias)	Unclear risk	No information available.
Allocation concealment (selection bias)	Unclear risk	No information available.
Baseline equivalence	Unclear risk	No information available.
Performance bias (confounding)	Unclear risk	No information available.
Detection bias (blinding of assessment): administrative data	Unclear risk	
Detection bias (blinding of assessors): participant reports	Unclear risk	No information available.
Attrition bias: administrative data	Unclear risk	
Attrition bias: participant reports	Unclear risk	No information available.
Standardised observation periods	Unclear risk	No information available.
Intention‐to‐treat	Unclear risk	No information available.
Selective reporting (reporting bias)	High risk	No public protocol available. Results were presented at a conference in 2005, but are not published or publicly available.
Validated outcome measures	Unclear risk	No information available.
Conflicts of interest	High risk	Dr. Holly B. Waldron is CEO of LIFFT, Co. (Leading Implementations in Functional Family Therapy), an organisation that disseminates FFT into community settings. Dr. Timothy Ozechowski is Director of Research for LIFFT.

Waldron, 2008a

**Table:** 

**Methods**	Youth who continued to use drugs after 6 weeks of group therapy (*n* = 140) were randomly assigned to FFT or CBT in the Albuquerque, NM (USA) metropolitan area.
**Participants**	Youth (ages 13–18) with alcohol or other substance use diagnosis (moderate to heavy users) who continued to use drugs after 6 weeks of group therapy.
**Interventions**	Eight weekly sessions of FFT were ‘designed to strengthen family relationships and build skills to help the adolescent stop or reduce his/her drug use’ (2008).
	Eight weekly sessions of individual cognitive behavioural therapy (CBT) aimed ‘to develop skills enabling adolescents stop or reduce their drug use’ (2008).
**Outcomes**	The protocol indicates that urine drug screens were collected at 6, 14, and 22 weeks, 3 and 6 months after the initial treatment session.
**Notes**	The protocol registration record (in clinicaltrials.gov) is linked to a study funded by the US NIH NIDA (DA 023568), with $3,039,371 in grants over 5 years (2007–2013) to Holly B. Waldron at the Oregon Research Institute Center for Family and Adolescent Research (ORI/CFAR). The protocol indicates that study was completed in August 2013. There are no published (or publicly available) reports on outcomes. Dr. Waldron did not respond to repeated requests for information about this study.

Risk of bias table

**Table:** 

Bias	Authors’ judgement	Support for judgement
Random sequence generation (selection bias)	Unclear risk	No information available.
Allocation concealment (selection bias)	Unclear risk	No information available.
Baseline equivalence	Unclear risk	No information available.
Performance bias (confounding)	Unclear risk	No information available.
Detection bias (blinding of assessment): administrative data	Unclear risk	
Detection bias (blinding of assessors): participant reports	Unclear risk	No information available.
Attrition bias: administrative data	Unclear risk	
Attrition bias: participant reports	Unclear risk	No information available.
Standardised observation periods	Unclear risk	No information available.
Intention‐to‐treat	Unclear risk	No information available.
Selective reporting (reporting bias)	High risk	The protocol for this study was submitted to clinicaltrials.gov in May 2008 (retrospectively). The study began in March 2008 and was completed in 2013. There are no published (or publicly available) reports on results.
Validated outcome measures	Unclear risk	No information available.
Conflicts of interest	High risk	Dr. Holly B. Waldron is the CEO of LIFFT, Co. (Leading Implementations in Functional Family Therapy), an organisation that disseminates FFT into community settings.

**Characteristics of excluded studies**

Alexander, 1971

**Table:** 

**Reason for exclusion**	Not a certified FFT program
Aultman‐Bettridge, 2007	
**Reason for exclusion**	Non‐randomised design without statistical controls for baseline differences between groups. Compared girls who completed FFT or MST (‘program youth’) with others.
Babbin et al., 2016	
**Reason for exclusion**	Not a certified FFT program
Barton, 1985 1	
**Reason for exclusion**	No parallel cohort (no comparison or control group)
Barton, 1985 2	
**Reason for exclusion**	Non‐randomised design without statistical controls for baseline differences between groups
Barton, 1985 3	
**Reason for exclusion**	Non‐randomised design without statistical controls for baseline differences between groups
Blower, 2017	
**Reason for exclusion**	Non‐randomised study with no controls for baseline differences between groups
Brent, 1988	
**Reason for exclusion**	Not a certified FFT program
Butcher, 2001	
**Reason for exclusion**	Non‐randomised design without statistical controls for baseline differences between groups
Cuellar, 2016	
**Reason for exclusion**	No parallel cohort (comparison of cases that were eligible vs. not eligible for FFT; also not an assessment of a certified FFT program)
Datchi, 2013	
**Reason for exclusion**	Does not meet age criterion (study of adults)
Doan, 2012	
**Reason for exclusion**	Non‐randomised design without statistical controls for baseline differences between groups
Eberts, 2012	
**Reason for exclusion**	Non‐randomised design without statistical controls for baseline differences between groups
Emerson, 1989	
**Reason for exclusion**	No parallel cohort (both groups received FFT)
Erickson, 2008	
**Reason for exclusion**	Non‐randomised design without statistical controls for baseline differences between groups
Friedman, 1989	
**Reason for exclusion**	Participants did not meet our age criterion (mean age = 17.9; range: 14–21 years)
Gan et al., 2016	
**Reason for exclusion**	No parallel cohort (single group, pre–post comparisons)
Gordon, 1995 1	
**Reason for exclusion**	Non‐randomised design without statistical controls for baseline differences between groups (court selected FFT cases)
Gordon, 1995 2	
**Reason for exclusion**	No parallel cohort
Gordon, 1995 3	
**Reason for exclusion**	Non‐random comparison group without statistical controls for baseline differences between groups (FFT cases lived closer to a university)
Graham, 2012	
**Reason for exclusion**	No parallel cohort (no comparison or control group)
Hanes, 2012	
**Reason for exclusion**	No parallel cohort (all cases received FFT)
Hansson, 2004	
**Reason for exclusion**	Non‐randomised design without statistical controls for baseline differences between groups
Herring‐Antwine, 2019	
**Reason for exclusion**	No parallel cohort (also, not a certified FFT program)
Hogue, 2013	
**Reason for exclusion**	Not a certified FFT program
Hogue, 2010	
**Reason for exclusion**	Not a certified FFT program (combines FFT with neuropsychological assessment and psychostimulant medication for ADHD)
Hops et al., 2011	
**Reason for exclusion**	Not a certified FFT program; Integrated Behavioural Family Therapy was ‘adapted from’ FFT.
Lewis, 1990	
**Reason for exclusion**	Not a certified FFT program
Limoncelli, 2019	
**Reason for exclusion**	No parallel cohort (no control or comparison group)
Markman, 2012	
**Reason for exclusion**	No parallel cohort (no comparison or control group)
Marshall, 2018	
**Reason for exclusion**	No parallel cohort (no control or comparison group)
Pederson, 2012	
**Reason for exclusion**	No parallel cohort (all cases received FFT)
Pettiford, 2009	
**Reason for exclusion**	No parallel cohort (all cases received FFT)
Renaud, 1998	
**Reason for exclusion**	Not a certified FFT program
Robin, 1981	
**Reason for exclusion**	Not a certified FFT program
Rohde, 2008	
**Reason for exclusion**	FFT was combined with another treatment (a course on adolescent Coping With Depression, CWD), either in sequence (FFT then CWD or CWD then FFT) or coordinated (simultaneous) provision of FFT and CWD.
Rowland, 2007	
**Reason for exclusion**	Not a certified FFT program
Saeidmanesh, 2019	
**Reason for exclusion**	Non‐randomised study without controls for baseline differences between groups
Satterfield, 2013	
**Reason for exclusion**	Non‐randomised study with no statistical controls for baseline differences between groups
Schawo, 2009	
**Reason for exclusion**	No parallel cohort (no comparison or control group)
Shakeshaft, 2020	
**Reason for exclusion**	Non‐randomised study with no controls for baseline differences between groups. (Most analyses focus on families who *completed* FFT compared with families who were not referred to FFT or MST. Analyses of families referred to FFT versus those not referred include statistical controls for correlations among outcomes within districts. Due to missing data, there were no statistical controls for age, gender, or Aboriginality. Characteristics of cases in comparison groups are not reported.)
Stout 2010	
**Reason for exclusion**	No parallel cohort (interrupted time series)
Stout, 2013	
**Reason for exclusion**	No parallel cohort (interrupted time series design)
Taxy, 2012	
**Reason for exclusion**	No parallel cohort (no comparison or control group)
Thurston, 2015	
**Reason for exclusion**	Randomised controlled trial stopped early, before the full sample was obtained and before outcome data were analysed. Reasons for stopping were: insufficient referrals, staff turnover, government take over of the service provider agency (due to concerns about management practices and risks to children), and lack of ongoing support for research. At the time this trial was stopped, 11 families had been randomly assigned out of a target sample of 76 (minimum) to 154 families.
Turner, 2014	
**Reason for exclusion**	No parallel cohort (plans for propensity score matching [2014] were not carried out; only results for FFT cases are reported [2019])
Turner, 2017	
**Reason for exclusion**	Does not meet our age criterion. Children's mean age = 9.7, SD = 5.1, range from 0 to 19. Forty percent (40%) of families had no children over age 10. Separate analysis were not conducted for older youth.
VanderPut 2012	
**Reason for exclusion**	Non‐randomised design without statistical controls for baseline differences between groups
Vardanian, 2020	
**Reason for exclusion**	No parallel cohort (no comparison or control group)
Waldron, 2008b	
**Reason for exclusion**	No parallel cohort (both groups received FFT, one group received additional pharmacological intervention)
Waldron, 2013	
**Reason for exclusion**	No parallel cohort (all cases received FFT; study investigated effects of different approaches to supervision)
Waldron, 2015	
**Reason for exclusion**	No parallel cohort (study of a system to track fidelity and assessment of FFT cases; all cases receive FFT)
Weintraub, 2019	
**Reason for exclusion**	Not a certified FFT program (used family‐focused therapy combined with pharmacotherapy).
White, 2010	
**Reason for exclusion**	No parallel cohort (no comparison or control group)
Wright, 1987	
**Reason for exclusion**	Non‐randomised design without statistical controls for baseline differences between groups (focuses on outcomes of staff training for staff; no client outcome data)
Zisk, 2019	
**Reason for exclusion**	Not a certified FFT program (compared Attachment‐Based Family Therapy with Family‐Enhanced Non‐Directive Supportive Therapy).

**Characteristics of studies awaiting classification**

Lantz, 1982

**Table:** 

**Methods**	Random assignment to FFT or alternative treatment. Post‐treatment assessment of recidivism and foster care placement.
**Participants**	46 delinquent adolescents
**Interventions**	FFT and alternative treatment (not defined)
**Outcomes**	Recidivism (not defined), foster care placement
**Notes**	Report not available

## SUMMARY OF FINDINGS TABLES

**Summary of findings**

**Table:** 

Outcomes 12 months post assignment	Relative effects FFT	95% CI	No. of studies (*k*)	No. of participants (*N*)	Certainty of evidence (GRADE)	Data source
Recidivism	OR = 0.79	0.45 to 1.41	6	1522	Very low	Analysis 1.1
Out of home placement (days)	SMD = 0.07	−0.21 to 0.35	2	203	Very low	Analysis 2.3
Externalising behaviour	SMD = −0.05	−0.35 to 0.24	2	183	Very low	Analysis 3.1
Internalising behaviour	SMD = 0.01	−0.28 to 0.31	2	184	Very low	Analysis 3.3
Self‐reported delinquency	SMD = 0.05	−0.15 to 0.25	5	468	Very low	Analysis 4.1
Alcohol and drug use	SMD = −0.29	−0.77 to 0.19	1	75	Very low	Analysis 5.2

## DATA AND ANALYSES

**1 Recidivism (arrest or conviction)**

**Table:** 

Outcome or subgroup	Studies	Participants	Statistical method	Effect estimate
1.1 Recidivism, 6–12 months	6	1522	Odds Ratio (M‐H, Random, 95% CI)	0.79 [0.45, 1.40]
1.1.1 RCTs	4	383	Odds Ratio (M‐H, Random, 95% CI)	0.58 [0.22, 1.52]
1.1.2 QEDs	2	1139	Odds Ratio (M‐H, Random, 95% CI)	1.14 [0.77, 1.68]
1.2 Recidivism, 12 months, FFT vs. no treatment	1		Odds Ratio (M‐H, Random, 95% CI)	No totals
1.2.1 RCTs	1		Odds Ratio (M‐H, Random, 95% CI)	No totals
1.3 Recidivism, 15–18 months	6	2550	Odds Ratio (M‐H, Random, 95% CI)	0.93 [0.78, 1.11]
1.3.1 RCTs	2	217	Odds Ratio (M‐H, Random, 95% CI)	0.78 [0.41, 1.49]
1.3.2 QEDs	4	2333	Odds Ratio (M‐H, Random, 95% CI)	0.95 [0.78, 1.16]
1.4 Recidivism, 24 months	2	789	Odds Ratio (M‐H, Random, 95% CI)	0.41 [0.06, 2.68]
1.4.1 RCTs	1	89	Odds Ratio (M‐H, Random, 95% CI)	0.15 [0.05, 0.40]
1.4.2 QEDs	1	700	Odds Ratio (M‐H, Random, 95% CI)	1.00 [0.75, 1.35]
1.5 Number of arrests, 6 months	1		Std. Mean Difference (IV, Random, 95% CI)	No totals
1.5.1 RCTs	1		Std. Mean Difference (IV, Random, 95% CI)	No totals
1.6 Number of arrests, 18 months	1		Std. Mean Difference (IV, Random, 95% CI)	No totals
1.6.1 RCTs	1		Std. Mean Difference (IV, Random, 95% CI)	No totals

**2 Out of home placement**

**Table:** 

Outcome or subgroup	Studies	Participants	Statistical method	Effect estimate
2.1 Placement, 6 months	1		Odds Ratio (M‐H, Random, 95% CI)	No totals
2.1.1 RCTs	1		Odds Ratio (M‐H, Random, 95% CI)	No totals
2.2 Placement, 15–18 months	2	283	Odds Ratio (M‐H, Random, 95% CI)	0.83 [0.49, 1.41]
2.2.1 RCTs	1	128	Odds Ratio (M‐H, Random, 95% CI)	0.91 [0.45, 1.85]
2.2.2 NRS	1	155	Odds Ratio (M‐H, Random, 95% CI)	0.73 [0.33, 1.65]
2.3 Days in placement, 6–12 months	2		Std. Mean Difference (IV, Random, 95% CI)	Subtotals only
2.3.1 RCTs	2	203	Std. Mean Difference (IV, Random, 95% CI)	0.07 [−0.21, 0.35]
2.4 Days in placement, 15–18 months	2		Std. Mean Difference (IV, Random, 95% CI)	Subtotals only
2.4.1 RCTs	2	203	Std. Mean Difference (IV, Random, 95% CI)	−0.04 [−0.62, 0.53]

**3 Youth behaviour problems and symptoms**

**Table:** 

Outcome or subgroup	Studies	Participants	Statistical method	Effect estimate
3.1 Externalizing behaviour, 6–12 months	2		Std. Mean Difference (IV, Random, 95% CI)	Subtotals only
3.1.1 RCTs	2	183	Std. Mean Difference (IV, Random, 95% CI)	−0.05 [−0.35, 0.24]
3.2 Externalizing behaviour, 15–18 months	2		Std. Mean Difference (IV, Random, 95% CI)	Subtotals only
3.2.1 RCTs	2	180	Std. Mean Difference (IV, Random, 95% CI)	−0.17 [−0.62, 0.27]
3.3 Internalizing behaviour, 6–12 months	2		Std. Mean Difference (IV, Random, 95% CI)	Subtotals only
3.3.1 RCTs	2	184	Std. Mean Difference (IV, Random, 95% CI)	0.01 [−0.28, 0.31]
3.4 Internalizing behaviour, 15–18 months	2		Std. Mean Difference (IV, Random, 95% CI)	Subtotals only
3.4.1 RCTs	2	179	Std. Mean Difference (IV, Random, 95% CI)	0.11 [−0.19, 0.41]
3.5 CBCL total, up to 36 months	1		Std. Mean Difference (IV, Random, 95% CI)	No totals
3.5.1 RCTs	1		Std. Mean Difference (IV, Random, 95% CI)	No totals

**4 Delinquency**

**Table:** 

Outcome or subgroup	Studies	Participants	Statistical method	Effect estimate
4.1 Delinquency scores, 6–12 months	5		Std. Mean Difference (IV, Random, 95% CI)	Subtotals only
4.1.1 RCTs	5	468	Std. Mean Difference (IV, Random, 95% CI)	0.05 [−0.15, 0.25]
4.2 Delinquency scores, 15–18 months	3		Std. Mean Difference (IV, Random, 95% CI)	Subtotals only
4.2.1 RCTs	3	262	Std. Mean Difference (IV, Random, 95% CI)	0.09 [−0.26, 0.43]

**5 Substance use**

**Table:** 

Outcome or subgroup	Studies	Participants	Statistical method	Effect estimate
5.1 Marijuana use, 6–12 months	3		Std. Mean Difference (IV, Random, 95% CI)	Subtotals only
5.1.1 RCTs	3	263	Std. Mean Difference (IV, Random, 95% CI)	0.02 [−0.23, 0.26]
5.2 Drug/alcohol use, 9 months	1		Std. Mean Difference (IV, Random, 95% CI)	No totals
5.2.1 RCTs	1		Std. Mean Difference (IV, Random, 95% CI)	No totals
5.3 Drug/alcohol use, 15 months	1		Std. Mean Difference (IV, Random, 95% CI)	No totals
5.3.1 RCTs	1		Std. Mean Difference (IV, Random, 95% CI)	No totals

**6 Peer relations**

**Table:** 

Outcome or subgroup	Studies	Participants	Statistical method	Effect estimate
6.1 Social skills, 6 months	1		Std. Mean Difference (IV, Random, 95% CI)	No totals
6.1.1 RCTs	1		Std. Mean Difference (IV, Random, 95% CI)	No totals
6.2 Social skills, 18 months	1		Std. Mean Difference (IV, Random, 95% CI)	No totals
6.2.1 RCTs	1		Std. Mean Difference (IV, Random, 95% CI)	No totals
6.3 Gang involvement, 6 months	1		Odds Ratio (M‐H, Random, 95% CI)	No totals
6.3.1 RCTs	1		Odds Ratio (M‐H, Random, 95% CI)	No totals

**7 Self esteem**

**Table:** 

Outcome or subgroup	Studies	Participants	Statistical method	Effect estimate
7.1 Youth self‐esteem, 7 months	1		Std. Mean Difference (IV, Random, 95% CI)	No totals
7.1.1 RCTs	1		Std. Mean Difference (IV, Random, 95% CI)	No totals
7.2 Youth self‐esteem, 2 months, FFT vs no treatment	1		Std. Mean Difference (IV, Random, 95% CI)	No totals
7.2.1 RCTs	1		Std. Mean Difference (IV, Random, 95% CI)	No totals

**8 Parent functioning**

**Table:** 

Outcome or subgroup	Studies	Participants	Statistical method	Effect estimate
8.1 Parenting skills, 6 months	1		Std. Mean Difference (IV, Random, 95% CI)	No totals
8.1.1 RCTs	1		Std. Mean Difference (IV, Random, 95% CI)	No totals
8.2 Parental care, 9 months	1		Std. Mean Difference (IV, Random, 95% CI)	No totals
8.2.1 RCTs	1		Std. Mean Difference (IV, Random, 95% CI)	No totals
8.3 Parental care, 15 months	1		Std. Mean Difference (IV, Random, 95% CI)	No totals
8.3.1 RCTs	1		Std. Mean Difference (IV, Random, 95% CI)	No totals
8.4 Parent functioning, up to 36 months	1		Std. Mean Difference (IV, Random, 95% CI)	No totals
8.4.1 RCTs	1		Std. Mean Difference (IV, Random, 95% CI)	No totals

**9 Family functioning**

**Table:** 

Outcome or subgroup	Studies	Participants	Statistical method	Effect estimate
9.1 Family functioning, 6‐7 months	2		Std. Mean Difference (IV, Random, 95% CI)	Subtotals only
9.1.1 RCTs	2	126	Std. Mean Difference (IV, Random, 95% CI)	0.19 [−0.16, 0.54]
9.2 Family functioning, up to 36 months	1		Std. Mean Difference (IV, Random, 95% CI)	No totals
9.2.1 RCTs	1		Std. Mean Difference (IV, Random, 95% CI)	No totals

**10 School**

**Table:** 

Outcome or Subgroup	Studies	Participants	Statistical Method	Effect Estimate
10.1 School attendance, any, 6 months	1		Odds Ratio (M‐H, Random, 95% CI)	No totals
10.1.1 RCTs	1		Odds Ratio (M‐H, Random, 95% CI)	No totals
10.2 School attendance, days/week, 6 months	1		Std. Mean Difference (IV, Random, 95% CI)	No totals
10.2.1 RCTs	1		Std. Mean Difference (IV, Random, 95% CI)	No totals

## SOURCES OF SUPPORT

**Internal sources**

- Bryn Mawr College, USA
  Support for Julia Littell

**External sources**

- Nordic Campbell Center, Denmark
  Support for review production
- Norwegian Knowledge Centre for the Health Services, Norway
  Support for review production and for Arild Bjørndal and Aina Winsvold
- The National Board of Health and Welfare (Socialstyrelsen), Sweden
  Support for review production and for Maria Bordin
- Youth Endowment Foundation, UK
  Support for review production via a grant to the Campbell Collaboration

## Supporting information

Supporting information.

Supporting information.

Supporting information.

## APPENDIX 1 Detailed search strategies

Database searches were performed in 2013‐14 and in August 2020, as described in the text. Minor changes in search strings and syntax were made in 2020, due to changes in databases or interfaces. In 2020 we imposed new date restrictions, to avoid duplication of previous searches. All such changes are noted below.

**APA PsycInfo** 1806 to August Week 4 2020 (29 August 2020, 87 hits):

1 functional family therapy.tw.

2 limit 1 to yr=“2013 ‐Current”

**ASSIA** (ProQuest) (04 August 2020, 60 hits):

ab(“functional family therapy”) OR ti(“functional family therapy”)

Date: After 01 January 2007

**Cambridge Core Collection** (30 August 2020, 2 hits):

“functional family therapy”

Published after 2006 ‐ Published before 2020

**CINAHL** (EbscoHost) (29 August 2020, 43 hits):

1 functional family therapy

Limiters ‐ Published Date: 20130101‐20201231

**Cochrane Library** (Wiley) All databases (30 August 2020, 56 hits including 1 protocol and 55 trials):

“functional family therapy” in Title Abstract Keyword

Custom year range: 2013‐2020

**Embase Classic+Embase** 1947 to 2020 August 28 (30 hits):

1 functional family therapy.tw.

2 (2013* or 2014* or 2015* or 2016* or 2017* or 2018* or 2019* or 202*).dc,dp,yr.

3 1 and 2

**ERIC** (OVID) 1965 to March 2020 (29 August 2020, 7 hits):

1 functional family therapy.tw.

2 limit 1 to yr=“2013 ‐Current”

**MEDLINE(R)** (Ovid) ALL 1946 to August 28, 2020 (31 hits):

1 functional family therapy.tw.

2 (2013* or 2014* or 2015* or 2016* or 2017* or 2018* or 2019* or 202*).dp,dt,ed,ep,yr.

3 1 and 2

**Norart** (29 August 2020, 2 hits):

“funksjonell familieterapi” OR “functional family therapy”

**Science Direct:** 1969 to 2013 March 03; 2013‐2020 (30 August 2020, 156 hits):

“functional family therapy”

*Refine by: Years 2013‐2020

**Social Care Online** (30 August 2020, 19 hits):

All fields: “functional family therapy”

AND Publication year: 2007 ‐ 2020

**Social Services Abstracts** (Proquest) (29 August 2020, 51 hits):

“functional family therapy”

Applied filters: 2013‐01‐01 ‐ 2020‐08‐31

**Sociological Abstracts** (Proquest) (29 August 2020, 36 hits):

“functional family therapy”

Additional limits ‐ Date: From 01 January 2013 to 31 August 2020

**SveMed+** (29 August 2020, 4 hits):

functional family therapy AND year:[2007 TO 2020]

funktionell familjeterapi AND year:[2007 TO 2020]

**Web of Science Core Collection** (29 August 2020, 65 hits):

TOPIC: (“functional family therapy”)

Refined by: PUBLICATION YEARS: (2020 OR 2019 OR 2018 OR 2017 OR 2016 OR 2015 OR 2014 OR 2013)

Indexes=SCI‐EXPANDED, SSCI, A&HCI, CPCI‐S, CPCI‐SSH, ESCI Timespan=All years

Science Citation Index Expanded (1900‐present)

Social Sciences Citation Index (1900‐present)

Arts & Humanities Citation Index (1975‐present)

Conference Proceedings Citation Index‐ Science (1990‐present)

Conference Proceedings Citation Index‐ Social Science & Humanities (1990‐present)

Emerging Sources Citation Index (2015‐present

**WorldCAT dissertation and theses** (OCLC) (30 August 2020, 24 hits:

kw: functional w family w therapy and yr: 2007‐2020

**Google scholar** (30 August 2020)

“functional family therapy” top 100 hits (sorted by relevance).

Custom date range: 2013 ‐ 2020

**Websites**

Searched on 30 August 2020 using the term “functional family therapy”:

U.S. Department of Health and Human Services (0 hits)

U.S. Centers for Disease Control and Prevention (5 hits)

U.S. Government Printing Office (0 hits)

UK Home Office (4 hits)

U.S. National Institutes of Health, RePORTer database (formerly CRISP) (searched 30 August 2020; hits: 221 projects, 9 publications, 6 clinical studies)

RePORTer database text search: “functional family therapy” (and), Search in: Projects, Publications and publication year limited between 2013 and 2020, Admin IC: All, Fiscal Year: Active Projects, 2020, 2019, 2018, 2017, 2016, 2015, 2014, 2013

## APPENDIX 2 Screening and data extraction forms

Overview: Steps in the screening and data extraction process

**Level 1:** Independent screening of **documents** based on titles and abstracts.

- If document is not excluded, retrieve full text and go to Level 2.
- Link multiple documents/reports to studies, assign study ID and report IDs.

**Level 2:** Independent coding of **study** eligibility criteria based on all documents associated with the study

- Compare results, resolve differences, and determine eligibility.

**Level 3:** Independent coding of **study** characteristics based on all documents associated with an eligible study

- Compare results and resolve differences.

**Level 4:** Independent coding of **outcome measures** (4a) and **outcome data** (4b)

- a) Code data on data collection measurement (questionnaires, interviews) and sources, compare results, and resolve differences.
- b) Enter outcome data, compare results, and resolve differences.

**Level 5**: Independent coding of data on study‐level **risk of bias**

- Compare results and resolve differences.
- Enter relevant data into RevMan.

**Level 1: Initial Screening (document level; from titles and abstracts)**

1.0. Procurement status: Is the abstract available?

1 Yes

0 No [STOP, find abstract]

1.1. Is this document about FFT (perhaps in addition to other topics)?

1 Yes

0 No [STOP, code as unrelated]

1.2. What is this? [select no more than ONE answer per document]

1. Study of FFT for medical conditions [STOP]

2. Review of FFT outcome studies [RETRIEVE FULL TEXT, STOP]

3. Descriptive, correlational, single‐group, or case study [STOP]

4. Theoretical or position paper, editorial, or book review [STOP]

5. Practice guidelines or treatment manual [STOP]

6. Can't tell [RETRIEVE FULL TEXT]

0 None of the above [ASSIGN THE DOCUMENT A STUDY ID AND REPORT ID]

**Level 2: Eligibility Decision (study level; based on full text, multiple reports if available)**

2.1. Does this study include two or more parallel cohorts (groups that received different treatments and were assessed at the same points in time)

1 Yes

0 No [STOP, excluded]

9 Can't tell

2.2. Is it a randomised experiment?

1 Yes [SKIP to 2.3]

0 No

9 Can't tell

2.2.1 Were any statistical controls used for baseline differences between groups? [Note: this question applies to ALL non‐randomised studies (and only to non‐randomised studies), regardless of whether groups were “matched” or whether baseline differences were reported. Check the methods sections and results to see if any statistical controls were used for participant characteristics at baseline. Propensity score matching does this; other statistical models that may use appropriate controls include: multivariate regression (e.g., OLS, logit, logistic regression), partial correlation, and hierarchical models.]

1 Yes [propensity score matching or at least one variable used to control for baseline differences between groups in analyses of outcomes]

0 No [STOP, excluded]

9 Can't tell

2.3. Does this study include a certified FFT program? [Include FFT‐G (gang) and FFT‐CW (child welfare) adaptations; exclude if FFT was always combined with pharmacotherapy or other intervention(s)]

1 Yes

0 No [STOP, excluded]

9 Can't tell

2.4. Does it include youth ages 11‐18?

1 Yes

0 No [STOP, excluded]

9 Can't tell

**Level 3: Data Extraction (study level; based on full text, multiple reports if available)**

Record in excel: Study ID, Revman name (surname of first author, date), Number of study reports retrieved, number of study reports not yet retrieved, Reviewer's initials

For remaining information, where possible please IDENTIFY REPORT NUMBERS AND PAGE NUMBERS WHERE INFORMATION WAS FOUND in notes or comments fields.

**Research method**

3.1. How were comparison groups formed?

3.3.1. Overall design:

1 RCT

0 other

3.3.2. Specific assignment methods

Random assignment methods
  1. Simple/systematic (individuals/families)
  2. Stratified/blocked (IDENTIFY STRATIFYING/BLOCKING VARIABLES)
  3. Yoked pairs (created by timing of enrollment into the study)
  4. Matched pairs (IDENTIFY MATCHING VARIABLES)
  5. Cluster (group) randomised [CHECK UNIT OF ANALYSIS]
  6. Other random method (SPECIFY)

Non‐random assignment methods
  1. Propensity score matching
  2. Other statistical controls for baseline characteristics (e.g., regression, ANCOVA)
  3. Other method (SPECIFY)

3.2. Who assigned cases to treatment/comparison groups?

1 Research staff

2 Program staff

3 Other (SPECIFY)

9 Unclear

3.3. How was group assignment performed?

1. Computer generated (random)

2. Random numbers table

3. Coins or dice

4. Envelopes or urns

5. Waitlist

6. Staff discretion

7. Other (DESCRIBE)

9. Unclear

3.4 Timing of enrollment into the study

3.4.a. Enrollment start date ______________

3.4.b. Enrollment end date _____________

3.5 Funding sources (list all) and contract/grant/project numbers if available

**Settings**

3.6. How many separate sites* were included in the study?

*Unique settings/geolocations with non‐overlapping samples (i.e., participants assigned to treatment/comparison groups in one site do not appear in program/control groups in other sites).

3.7. Location types (location of interventions)

1 Urban

2 Suburban

3 Rural

4 Mixed types

9 Unclear

3.8. Country

1 USA

2 Canada

3 Norway

4 Sweden

5 Netherlands

6 UK

7 Other (describe)

8 Multiple (describe)

9 Unclear

Location details (city, state/province) ________________

3.9. Primary service sector

1 Juvenile justice

2 Mental Health

3 Child Welfare

4 Multiple or other (specify)

9 Unclear

3.10. Briefly describe sample inclusion criteria (narrative)

**Sample Characteristics**

3.11. Caseflow and sample size

**Table:**

Number of cases	FFT	Control	Total	Pg# & Notes
Referred to study			a	
Consented			b	
Randomly assigned	c	d		
Completed treatment	e	f		

3.12. Are outcome data available for program completers only?

1 Yes

0 No

9 Unclear

3.13. Demographic characteristics

**Table:**

		Total sample	Pg# & Notes
Gender	% Male		
Age of focal youth at beginning of treatment	Mean, sd		
	Min, Max		
Race/ethnicity	% White		
	% Black		
	% Hispanic/Latinx		
	% Asian/Pacific		
	% Other		
Socioeconomic status (parents)	% college educ		
	% unemployed		
	% receive public aid		
Family composition	% single parent home		
	% in out of home care		
	Mean # of children		
Other sample characteristics			

3.14. Were there any significant differences between program and control groups *at baseline* (p‐value < .05 and/or d > 0.25)? [IF NECESSARY, CALCULATE d USING ES_Calculator.xls]

1 Yes (describe differences using p and/or d values)

0 No (how do we know?)

9 Unclear (p and d not provided)

3.15. Were there any significant differences between *FFT program* completers and drop‐outs (p< .05 and/or d > 0.25)? [IF NECESSARY, CALCULATE d USING ES_Calculator.xls]

1 Yes (describe differences using p and/or d values)

0 No (how do we know?)

9 Unclear (p and d not provided)

3.16. Were there any significant differences between completers and drop‐outs *in the control group(s)* (p< .05 and/or d > 0.25)? [IF NECESSARY, CALCULATE d USING ES_Calculator.xls]

1 Yes (describe differences using p and/or d values)

0 No (how do we know?)

9 Unclear (p and d not provided)

**3.17. FFT Service Characteristics**

**Table:**

	Mean	SD	Min	Max	Pg# & Notes
a. Duration in of services (in days)					
b. Total hours of contact					

3.18. Other characteristics of FFT services (describe)

3.19. Characteristics of FFT staff (education, demographics, etc.)

3.20. Describe methods used to ensure quality of FFT services, if any (e.g., supervision, training, consultation)

3.21. Is there any information on program adherence (fidelity) to FFT?

1 Yes (describe)

0 No

9 Unclear

3.22. Are there any implementation differences between sites? (any qualitative/quantitative differences)

1 Yes (describe differences)

0 No (how do we know?)

9 Unclear

3.23. Is there any information on FFT program costs?

2 Yes, information is provided on costs per case (record amount)

1 Yes, information on total costs (record amount)

0 No

**Services provided to control cases**

3.24. Type of control group(s)

1 Usual services (treatment as usual)

2 Alternative service (DESCRIBE)

0 No service

3.25. Describe services provided to control group

- Duration of services in days (include mean,sd, min, max if available)
- Total hours of contact (include mean,sd, min, max if available)

3.26. Briefly describe characteristics of staff who provided services to control cases (education, demographics, etc.)

**Data collection methods (study level)**

3.27. Were research interviews conducted with program participants or others?

1 Yes

0 No [SKIP to 3.32]

9 Unclear

3.28. Who conducted interviews?

1 Researchers

2 Program staff

3 Others (describe)

9 Unclear

3.29. Who participated in interviews? (enter all that apply)
  - Youth
  - Parent(s)/Caregiver(s)
  - Teachers
  - Clinicians
  - Other (SPECIFY)

3.30. When did interviews occur? (List all data collection points in terms of number of average months since baseline, with baseline=0).

3.31. Were interviews conducted in the same manner for FFT and control groups (using the same interviewers, instruments, types of data sources, time points)?

1 Yes

0 No (what were the differences?)

9 Can't tell

3.32 Were administrative data collected (e.g., police records, hospital records, payment records)

1 Yes (what types of records)

0 No

9 Can't tell

3.33. If administrative data were collected, describe the timing of this data collection (list all data collection points, in terms of average months since baseline).

**Level 4a: Outcome measures (measurement level)**

Instructions: Enter each conceptually distinct measure (e.g., distinct subscales within instruments, plus overall scales) on a separate row, regardless of whether data were collected (at the time of the report) or reported. Note that a single outcome measure can be completed by multiple sources, at multiple points in time (data from specific sources and multiple time‐points will be entered later)

4a.1. Study ID

4a.2. Report ID (If multiple reports are available, enter information from reports in numerical order.)

4a.3. Outcome ID (Enter outcome measures in the order in which they are described in the measurement and results sections.)

4a.4. Coder initials

4a.5. Page #s where information was found

4a.6. Outcome type (domain) code

1 = Placement in out‐of‐home setting (jail, hospital, residential treatment, foster care)

2 = Arrest/conviction/probation

3 = Substance use

4 = Delinquency

5 = Peer relations (include social skills)

6 = Youth behaviour and symptoms

7 = Parent behaviour and symptoms (include parental monitoring, supervision, discipline)

8 = Family functioning (include cohesion, adaptability, communication)

9 = School (grades, attendance)

4a.7. Brief description of outcome measure

4a.8. Instrument name or definition

4a.9. Is this a scale or composite?

2 = total scale or composite (e.g., CBCL total score, any drug/alcohol use)

1 = main scale or type (e.g., CBCL internalizing, CBCL externalizing; illicit drug use)

0 = subscale or category (e.g., CBCL subscales; marijuana use)

4a.10. Timing: number of months since referral (for each planned measure)

4a.11. Information on the measure's reliability and validity

1 = from other samples only

2 = from this sample only

3 = both other samples and this sample

4 = none

9 = unclear

4a.12. Format: Is this a dichotomous or continuous measure?

0 = Dichotomous

1 = Continuous

4a.13. Direction: A high score or occurrence of an event is viewed as

1 = Positive

0 = Negative

9 = Unclear

4a.14. Sources (identify all relevant sources):

Y = Youth

P = Parent/caregiver

T = Teacher

C = Clinician

A = Administrative data

O = Other

Unclear

4a.15. How are data from multiple sources reported?

1 = Separately

2 = Averaged

3 = Selected (one source is used)

9 = Unclear

4a.16. Mode of administration

1 = Self (paper forms or direct input to computer)

2 = Interview

3 = Administrative data (collected by researchers)

4 = Other (explain)

4a.17. Were assessors blind to participant's treatment group?

1 = Yes

0 = No

9 = Unclear

**Level 4b: Outcome data (ES level)**

Instructions: Complete one row for each effect size (separate rows for different measures, data sources, and data points)

4b.1. Study ID

4b.2. Site specific data? 0 = no, 1 = site number

4b.3. Report ID

4b.4. Coder initials

4b.5. Page number

4b.6. Outcome ID: numbered consecutively for each study

4b.7. Outcome type code (refers to the domain codes described above)

4b.8. Description (brief name or phrase)

4b.9. Type of score

2 = total/composite scale (e.g., CBCL total score; any drug/alcohol use)

1 = main scale (e.g., CBCL externalizing, CBCL internalizing; illicit drug use)

0 = subscale/subtype (e.g., CBCL subscales; marijuana use)

4b.10. Format: 0 = dichotomous, 1 = continuous

4b.11. High score or event is positive? 0 = no, 1 = yes

4b.12. Timing of data collection: mean n months since referral

4b.13. Timing of data collection: range (max minus min months since referral)

4b.14. Observation period begins at referral? (for event data only)

0 = no

1 = yes

8 = NA (data collected at a single point in time, via interviews or self‐administered forms)

4b.15. Data type

1 = report

2 = observed by researchers

3 = biologic

4 = administrative data

4b.16. Data source

1 = youth

2 = parent/guardian or primary caregiver

3 = professional (teacher or clinician)

4 = administrative data

5 = multiple

6 = observer (researcher)

4b.17. Did the researchers impute missing data for this outcome?

0 = no

1 = yes

8 = can't tell

4b.18. Did researchers adjust results for baseline differences?

0 = no

1 = yes [identify variables used to adjust results]

8 = can't tell

4b.19. Raw data: For each valid comparison, record each group valid N, n or % with event. Record ES statistics (OR/SMD and CI) only if raw data are not available.

Dichotomous outcomes

**Table:**

	FFT			Comparison				95% CI	
	valid N	n event	%	valid N	n event	%	OR	LB	UB
Data									

Continuous outcomes

**Table:**

	FFT			Comparison				95% CI	
	Valid N	mean	sd	valid N	mean	sd	SMD	LB	UB
Data									

4b.20. Is there any missing data for calculation of ES and variance? [missing valid Ns, means, SDs]

0 = no

1 = yes

**Level 5: Risk of bias (study level; based on full text, multiple reports if available)**

IDENTIFY REPORTS AND PAGE NUMBERS WHERE RELEVANT INFORMATION WAS FOUND; BRIEFLY EXPLAIN EACH JUDGEMENT.

5.1. **Adequate sequence generation** (selection bias): investigators describe a random component in the sequence of assignments (e.g., computer‐generated random numbers, table of random numbers, drawing lots or envelopes, coin tossing, shuffling cards, or throwing dice).

1 Yes = Low risk of bias

2 Unclear: insufficient information (e.g., random assignment is mentioned, but not described in detail)

3 No = High risk: investigators describe a non‐random component in the sequence of assignments (e.g., alternation or rotation, date of birth, date of admission or referral, case record number, clinical judgement, client preference, or service availability; nonrandom addition, replacement, or removal of cases)

5.2. **Adequate allocation concealment** (selection bias): participants and investigators could not foresee assignment, because randomisation was performed at central site remote from the trial location or investigators monitored use of assignments contained in sequentially numbered, sealed, opaque envelopes.

1 Yes = Low risk

2 Unclear: insufficient information (e.g., random assignment is mentioned, but not described in detail) or adequacy of concealment is unclear (e.g., use of coin toss, card shuffle, dice, envelopes with unspecified characteristics)

3 No = High risk: allocation was not adequately concealed; for example, investigators used open random number lists, transparent or unsealed envelopes, or quasi‐randomisation methods such as alternation or rotation, date of birth, date of admission or referral, case record number, or service availability.

5.3. **Baseline equivalence**: initial differences between groups were small or moderate (d < 0.25). [USE ES_Calculator.xls TO CALCULATE d FOR BASELINE DATA; RECORD d STATISTICS.]

1 Yes = Low risk

2 Unclear risk: insufficient information (e.g., group‐level on background characteristics were not provided, d cannot be computed)

3 No = High risk: there were baseline differences between groups with **d > 0.25**.

5.4. **Avoidance of performance bias** (confounding): no systematic differences between groups in levels of care or attention, or in exposure to factors other than the interventions of interest.

1 Yes = Low risk

2 Unclear (insufficient information)

3 No = High risk: one group received more attention, care, or surveillance than another; or factors likely to be related to outcomes (confounding factors) were unequally distributed between groups.

5.5. **Avoidance of detection bias** (blinding): assessor is unaware of group assignment when collecting outcome data.

5.5.a. For administrative data:

1 Yes for all outcomes = Low risk

2. Yes for some outcomes = Unclear

2 Unclear (insufficient information)

3. No = High risk

5.5.b. for participant reports:

1. Yes for all outcomes = Low risk

2. Yes for some outcomes = Unclear

2 Unclear (insufficient information)

3. No = High risk

5.6. **Avoidance of attrition bias**: Losses to follow up were less than or equal to 25% and equally distributed (< 10% difference in response rates) across groups. Group equivalence on important baseline characteristics was retained after losses to follow‐up (d < 0.25). [RECORD ATTRITION AND D STATISTICS]

5.6.a. for administrative data:

1. Yes for all outcomes = Low risk

2. Yes for some outcomes = Unclear

2 Unclear (insufficient information)

3. No = High risk: loss of baseline equivalence (d > 0.25), losses to follow up > 25% overall, or losses were unequally distributed (>10% difference) across groups

5.6.b. for participant reports:

1. Yes for all outcomes = Low risk

2 Yes for some outcomes = Unclear

2 Unclear (insufficient information)

3 No = High risk: loss of baseline equivalence (d > 0.25), losses to follow up > 25% overall, or losses were unequally distributed (>10% difference) across groups

5.7. **Intention‐to‐treat:** data were analyzed according to participants’ initial group assignment, regardless of whether assigned services were received or completed.

1. Yes for all outcomes = Low risk

2 Yes for some outcomes = Unclear

2 Unclear (insufficient information)

3. No = High risk

5.8. **Standardized observation periods**: follow‐up data were collected from each case at a fixed point in time after random assignment, or analyses included controls for variable observation periods.

1. Yes for all outcomes = Low risk

2. Yes for some outcomes = Unclear

2 Unclear (insufficient information)

3 No = High risk

5.9. **Validated outcome measures**: use of instruments with demonstrated reliability (e.g., alpha/kappa > .7) or validity in this sample or similar samples, or use of external administrative data on events (e.g., arrests, incarceration, hospitalization). [RECORD ALPHA/KAPPA STATISTICS]

1. Yes for all outcomes = Low risk

2 Yes for some outcomes = Unclear

2. Unclear (insufficient information)

3 No = High risk

5.10. Free of **selective reporting:** a prospective study protocol is available, and all pre‐specified outcomes are reported in the pre‐specified way; all expected outcomes are reported in full and for all cases (regardless of direction and significance of results).

1 Yes = Low risk [DOCUMENT HOW WE KNOW THAT THE PROTOCOL WAS REGISTERED BEFORE THE STUDY BEGAN]

2 Unclear (e.g., a protocol is not available, the protocol appeared or was changed after the study began) [RECORD DATES OF PROTOCOL REGISTRATION/CHANGES]

3 No = High risk: some outcomes are not reported or are reported incompletely (e.g., for subgroups only or without sufficient detail for meta‐analysis).

5.11. Free of **conflicts of interest**: investigators state that they have no conflicts of interest. Investigators would not benefit if results favored FFT *or* control/comparison groups. None of the study authors, data collection staff, or data analysts were paid to develop, supervise, or provide services to the FFT or to the comparison group; none of these investigators are members of consulting firms linked to FFT or comparison conditions.

1. Yes = Low risk

2. Unclear (insufficient information, no conflict of interest statement)

3. No = High risk

5.12. Were **FFT program developers** (James Alexander, Thomas Sexton, Holly Waldron, Michael Robbins) involved in study design, implementation, or analysis?

0 No

1 Yes

## APPENDIX 3 Glossary of Outcome Measures

**Table:**

Measurement Instrument	Definition
ADI	Adolescent Drinking Index
CAI	Child Attachment Interview
CBCL	Child Behaviour CheckList
CBCL YSR	CBCL Youth Self Report
CDISC	Clinical Data Interchange Standards Consortium (diagnostic criteria)
CTS	Conflict Tactics Scale
CHOS	Confusion, Hubbub and Order Scale
CSI	Crime Severity Index
CTRS	Connors Teacher Rating Scale
BDI	Beck Depression Inventory
FAM‐III	Family Assessment Measure
FCI	Family Concept Inventory
FEC	The Family Environment Scale
IMS	Index of Marital Satisfaction
KASAM	Kansla Av SAMmanhang (Swedish), Sense of Coherence scale
NYSDS	National Youth Survey Delinquency Scale
OQ‐45.2	Outcome Questionnaire‐45.2
PBI	Parental Bonding Instrument
PCCB	Parent Checklist of Child's Behaviour
POSIP	Problem Oriented Screening Instrument for Parents
POSIT	Problem Oriented Screening Instrument for Teenagers
POSIT‐FU	Problem Oriented Screening Instrument for Teenagers ‐‐ Follow‐up
SCL‐90	Symptom CheckList 90
Sjalvrapporterad asocialitet	Self‐reported delinquency (Swedish)
TLFB	Timeline Follow Back Interview
TSCS	Tennessee Self Concept Scale
WSJC	Washington State Juvenile Court Prescreen
WWPAS	Werry‐Weiss‐Peters Activity Scale
YSR	Youth Self Report (on the CBCL)
