# Supplementary material for: Functional Family Therapy for families of youth (age 11–18) with behaviour problems: A systematic review and meta‐analysis
Source: Campbell Syst Rev. 2023 Jul 19;19(3):e1324. doi: 10.1002/cl2.1324 (PMC10354626; doi:10.1002/cl2.1324)
Supplement: Supplementary file 1 — Supporting information. [file CL2-19-e1324-s002.docx]

FFT systematic review codebook (Littell et al., 2023)

StudyID

| Sorted by study name | | Sorted by study ID | |
| --- | --- | --- | --- |
| ID | Study name | ID | Study name |
| 2 | Alexander 1973 | 2 | Alexander 1973 |
| 42 | Baglivio 2014 | 5 | Regas 1983 |
| 18 | Barnoski 2002 | 14 | Hansson 2000 |
| 48 | Carr 2014 | 17 | Waldron 2001 |
| 49 | Celinska 2013 | 18 | Barnoski 2002 |
| 50 | Darnell 2015 | 19 | Hollimon 2004 |
| 24 | Dunham 2009 | 20 | Slesnick 2004 |
| 52 | Eeren 2018 | 24 | Dunham 2009 |
| 47 | Godfredson 2018 | 39 | Ogden 2013 |
| 14 | Hansson 2000 | 40 | Humnayan 2010 |
| 19 | Hollimon 2004 | 41 | Waldron 2005 |
| 40 | Humnayan 2010 | 42 | Baglivio 2014 |
| 39 | Ogden 2013 | 47 | Godfredson 2018 |
| 66 | Ozechowski 2012 | 48 | Carr 2014 |
| 5 | Regas 1983 | 49 | Celinska 2013 |
| 75 | Robbins 2021 | 50 | Darnell 2015 |
| 20 | Slesnick 2004 | 52 | Eeren 2018 |
| 17 | Waldron 2001 | 53 | Waldron 2008a |
| 41 | Waldron 2005 | 66 | Ozechowski 2012 |
| 53 | Waldron 2008a | 75 | Robbins 2021 |

TwoArms: more than two arms (relevant comparison groups)? 0=no 1=yes

ReportID: report ID (sequential)

EffectID: ES ID (sequential)

Outcome: outcome type

1=Placement

2=Arrest/convict/proba

3=Substance use

4=Delinquency

5=PeerRelations

6=YouthBehSympt

7=ParentBehSympt

8=FamilyFunction

9=School

Out2: Outcome detail

1=Placement

2=Arrest/convict/proba

3=Substance use

4=Delinquency

5=PeerRelations

6=YouthSymptGen

7=YouthInternalizing

8=YouthExternalizing

9=ParentSympSupport

10=ParentingBeh

11=FamilyFunctionGen

12=FamCohesion

13=FamAdaptability

14=FamConflict

15=School

Subscale: Composite, main scale, or subscale

2=Total/composite (e.g., CBCL total)

1=Scale/main type (e.g., CBCL internalizing, externalizing)

0=Subscale/subtype

Continuous: 0=Dichotomous 1=Continuous

Positive: High score is positive? 0=no 1=yes

MonthsRef: Timing of data collection: mean n months since referral

MonthsRange: Timing of data collection: range (max - min months since referral)

ObsPeriod: Observation period: begins at referral?  0=no 1=yes 8=NA (point in time)

DataType: Data type 1=report 2=observed 3=biologic 4=admin data

Source: Data source 1=youth 2=parent 3=professional 4=admin data 5=multiple 6=observer

Imputed: Imputed missing data? 0=no 1=yes

AdjBase: adjusted for baseline diffs? 0=no 1=yes

FFTN: FFT valid N

FFTEvent: FFT n event

FFTPercent: FFT %

FFTmean: FFT mean

FFTsd: FFT sd

CntlN: Control valid N

CntlEvent: Control n event

CntlPerc: Control %

Cntlmean: Control mean

Cntlsd: Control sd

Smd

Smdlb: smd lower bound

Smdub: smd upper bound

Or

Orlb

Orub

Rr

Rrlb

Rrub

Rd

Rdlb

Rdub

Beta

Betalb

Betaub

Pval: exact p-value

missingES: missing stats for ES & variance? 0=no 1=yes

attrition: overall attrition (%)

diffatt: djfferential attrition (%)

TotalN: Full sample size (N)

FFTassign: N assigned to FFT

Cntlassign: N assigned to control

Country: 1=US 2=Netherlands 3=Norway 4=Sweden 5=Ireland 6=UK

US: Country2 1=US 0=other

Sector: Service sector: 1=juvenile justice 2=mental health 3=child welfare 4=multiple

Sampletype: Sample type 1=sex offenders 2=offenders 3=delinqency / beh/MH probs 4=ASD 5=CAN 6=SUD 7=serious MH

FFTtype: FFT type 1=original 2=Gang 3=CW 4=SUD

ComCond: comparison condition 1=TAU 2=other 3=no tx

CompDetail: comparison detail 1=TAU 2=indiv tx 3=family tx 4=group tx 10=no tx

Yearstart: year enrollment started

Yearend: year enrollment ended

MeanAge: mean age (years)

Percmale: % male

Percwhite: % white

Percblack: % black

Perchispanic: % hispanic

Location: Location type: 1=urban 2=suburban 3=rural 4=mixed 9=unclear

SitesN: number of sites

Protocol: Protocol? 0=no 1=retro 2=prospectv

RCT: RCT? 0=no 1=yes

Seqgen: ROB sequence generation 1=low risk 2=unclear 3=high risk

Allocation: ROB allocation concealment 1=low risk 2=unclear 3=high risk

Baseline: ROB baseline equivalence 1=low risk 2=unclear 3=high risk

Performbias: ROB perform bias 1=low risk 2=unclear 3=high risk

Detection: ROB detection bias 1=low risk 2=unclear 3=high risk

Robattrition: ROB attrition 1=low risk 2=unclear 3=high risk

Robitt: ROB ITT 1=low risk 2=unclear 3=high risk

Robstdobs: ROB std observations 1=low risk 2=unclear 3=high risk

Robmeasures: ROB valid measures 1=low risk 2=unclear 3=high risk

Selreporting: ROB selective reporting 1=low risk 2=unclear 3=high risk

Coi: ROB conflict of interest 1=low risk 2=unclear 3=high risk

Developers: FFT developers involved in study design, implementation, or analysis? 1=yes 0=no

Alexander: Alexander co-author? 1=yes 0=no

Sexton: Sexton co-author? 1=yes 0=no

Waldron: Waldron co-author? 1=yes 0=no
