# Supplementary material for: Functional Family Therapy for families of youth (age 11–18) with behaviour problems: A systematic review and meta‐analysis
Source: Campbell Syst Rev. 2023 Jul 19;19(3):e1324. doi: 10.1002/cl2.1324 (PMC10354626; doi:10.1002/cl2.1324)
Supplement: Supplementary file 3 — Supporting information. [file CL2-19-e1324-s001.html]

FFT Analysis


# FFT Analysis

## Read in the Data and Produce Descriptives

The code below activates the relevant programs and reads in the data.

```
library(metafor)
```

```
Loading required package: Matrix
```

```
Loading required package: metadat
```

```
Loading the 'metafor' package (version 3.8-1). For an
introduction to the package please type: help(metafor)
```

```
library(tidyverse)
```

```
── Attaching packages
───────────────────────────────────────
tidyverse 1.3.2 ──
```

```
✔ ggplot2 3.4.0      ✔ purrr   1.0.0 
✔ tibble  3.1.8      ✔ dplyr   1.0.10
✔ tidyr   1.2.1      ✔ stringr 1.5.0 
✔ readr   2.1.3      ✔ forcats 0.5.2 
── Conflicts ────────────────────────────────────────── tidyverse_conflicts() ──
✖ tidyr::expand() masks Matrix::expand()
✖ dplyr::filter() masks stats::filter()
✖ dplyr::lag()    masks stats::lag()
✖ tidyr::pack()   masks Matrix::pack()
✖ tidyr::unpack() masks Matrix::unpack()
```

```
library(robumeta)

# read in the FFT data file
fftall <- read.csv("FFT12-20-22.csv")


# frequencies on number of continuous ES vs dichotomous ES
table(fftall$continuous)
```

```
  0   1 
 63 243
```

## Compute SMD for continuous outcomes

This code produces the SMDs for the relevant studies. First, those studies with means, sds and sample sizes are computed using escalc. Study 47 has reported effect sizes and sample sizes. For Study 47, the reported effect sizes are corrected with Hedges’ small sample size correction and the variance of the SMD is computed with the small sample correction applied. The last step is to change the direction of the effect sizes so that a positive effect size indicates an outcome in favor of FFT.

```
# compute the smd for cases with means and sds

fftall <- escalc(measure = "SMD",
                 m1i = FFTmean,
                 sd1i = FFTsd,
                 n1i = FFTN,
                 m2i = Cntlmean,
                 sd2i = Cntlsd,
                 n2i = CntlN,
                 data = fftall,
                 var.names = c("SMD1", "varsmd"))

# descriptives for smd and varsmd
summary(fftall$SMD1)
```

```
    Min.  1st Qu.   Median     Mean  3rd Qu.     Max.     NA's 
-2.24361 -0.21401  0.03639  0.04746  0.23822  1.34442       98
```

```
summary(fftall$varsmd)
```

```
   Min. 1st Qu.  Median    Mean 3rd Qu.    Max.    NA's 
0.01905 0.05734 0.08194 0.14779 0.33352 0.54308      98
```

```
# Study 47 has reported means and smd
# compute the variance of smd and apply small sample correction
# first subset the data: all but 47, one data set for 47 

fftnot47 <- subset(fftall , StudyID != 47)
fft47 <- subset(fftall, StudyID == 47)


# correct the reported SMD for Hedges' small sample correction
fft47$SMD1 <- 
  fft47$smd*(1-(3/((4*(fft47$FFTN+fft47$CntlN))-9)))

# compute the variance for SMD using sample sizes and reported smd
# and correct for Hedges' small sample correction
fft47$varsmd <- ((fft47$FFTN+fft47$CntlN)/(fft47$FFTN*fft47$CntlN)+
  fft47$SMD^2/(2*(fft47$FFTN+fft47$CntlN)))*
  (1-(3/((4*(fft47$FFTN+fft47$CntlN))-9)))

# look at descriptives on SMD and varSMD for study 47
summary(fft47$SMD1)
```

```
    Min.  1st Qu.   Median     Mean  3rd Qu.     Max.     NA's 
-0.40756 -0.13666 -0.01987 -0.03750  0.07451  0.28800       25
```

```
summary(fft47$varsmd)
```

```
   Min. 1st Qu.  Median    Mean 3rd Qu.    Max.    NA's 
0.03109 0.03379 0.03430 0.03446 0.03557 0.04570      25
```

```
# add study 47 SMDs to full data set
fftall2 <- rbind.data.frame(fftnot47, fft47)

# change direction of effect sizes so that positive effect sizes
# are in favor of FFT

# descriptives of positive versus negative direction
table(fftall2$Positive)
```

```
  0   1 
219  87
```

```
# recode to new SMD 
fftall2 <-
  fftall2 %>%
  mutate(
    SMD = if_else(Positive == 0, -SMD1, SMD1)
    )

summary(fftall2$SMD1)
```

```
    Min.  1st Qu.   Median     Mean  3rd Qu.     Max.     NA's 
-2.24361 -0.21195  0.02851  0.03802  0.21388  1.34442       72
```

```
summary(fftall2$SMD)
```

```
    Min.  1st Qu.   Median     Mean  3rd Qu.     Max.     NA's 
-0.56020 -0.13032  0.05506  0.14819  0.29159  2.24361       72
```

```
hist(fftall2$SMD)
```

## Compute LOR for dichotomous outcomes

The code below uses escalc to compute the log-odds ratio for relevant studies. First, the log-odds ratio is computed for those studies with information from the 2x2 table. Second, studies 49 and 50 report the odds ratio and its 95% confidence interval. For these studies, the log-odds ratio is computed along with its variance. Finally, the log-odds ratios are corrected so that a positive log-odds ratio indicates an outcome in favor of FFT. There is a large outlier (outcome 16, study 49) in the data. The odds ratio is around 31.

```
# compute LOR for the dichotomous outcomes
fftall2 <- escalc(measure = "OR",
                  ai = FFTEvent,
                  n1i = FFTN,
                  ci = CntlEvent,
                  n2i = CntlN,
                  data = fftall2,
                  var.names = c("LOR1", "varLOR"))

# examine descriptives
summary(fftall2$LOR1)
```

```
    Min.  1st Qu.   Median     Mean  3rd Qu.     Max.     NA's 
-3.44999 -0.65743 -0.31449 -0.39224  0.00367  1.06031      257
```

```
summary(fftall2$varLOR)
```

```
   Min. 1st Qu.  Median    Mean 3rd Qu.    Max.    NA's 
0.01381 0.14088 0.22182 0.26519 0.32657 0.86909     257
```

```
# studies 49 and 50 have reported OR and confidence intervals
# subset data to those with CIs and those without CIs

fftworlb <- subset(fftall2, is.na(fftall2$orlb) != T)
fftnoorlb <- subset(fftall2, is.na(fftall2$orlb) == T)

# compute LOR for studies with reported OR and confidence intervals

fftworlb$LOR1 <- log(fftworlb$or)
fftworlb$varLOR <- ((log(fftworlb$orlb)-log(fftworlb$orub))/(2*1.96))^2


# put data set back together
fftall3 <- rbind.data.frame(fftworlb, fftnoorlb)

# change direction of effect sizes so that positive effect sizes
# are in favor of FFT

# descriptives of positive versus negative direction
table(fftall3$Positive)
```

```
  0   1 
219  87
```

```
# recode to new LOR 
fftall3 <-
  fftall3 %>%
  mutate(
    LOR = if_else(Positive == 0, -LOR1, LOR1)
    )

fftwrong <- subset(fftall3, LOR > 3)
# descriptives of original and direction corrected LORs
summary(fftall3$LOR1)
```

```
    Min.  1st Qu.   Median     Mean  3rd Qu.     Max.     NA's 
-3.44999 -0.83370 -0.32267 -0.45834  0.00043  1.06031      247
```

```
summary(fftall3$LOR)
```

```
    Min.  1st Qu.   Median     Mean  3rd Qu.     Max.     NA's 
-1.06031 -0.04196  0.31449  0.43605  0.83370  3.44999      247
```

```
hist(exp(fftall3$LOR))
```

```
summary(exp(fftall3$LOR))
```

```
   Min. 1st Qu.  Median    Mean 3rd Qu.    Max.    NA's 
 0.3463  0.9596  1.3696  2.4819  2.3020 31.5000     247
```

```
# save the data set with the effect sizes

write.csv(fftall3, file = "FFTES.csv")
```

## SMD Analysis: Overall Mean

The code below estimates the overall mean SMD from the relevant studies, and computes the overall mean SMD and its standard error using robumeta. The sensitivity of the results to assumptions about the correlation among effect sizes is also provided. The 95% prediction interval is also computed.

```
# read in the data
fftES <- read.csv("FFTES.csv")

# use robumeta to compute the mean SMD with RVE

FFTSMDCE <- robu(formula = SMD ~ 1, data = fftES,
                 studynum = StudyID, var.eff.size = varsmd,
                 rho = 0.8, small = TRUE)
FFTSMDCE
```

```
RVE: Correlated Effects Model with Small-Sample Corrections 

Model: SMD ~ 1 

Number of studies = 10 
Number of outcomes = 234 (min = 1 , mean = 23.4 , median = 12 , max = 76 )
Rho = 0.8 
I.sq = 62.57427 
Tau.sq = 0.08623334 

               Estimate StdErr t-value  dfs P(|t|>) 95% CI.L 95% CI.U Sig
1 X.Intercept.   0.0717  0.107   0.669 8.14   0.522   -0.175    0.318    
---
Signif. codes: < .01 *** < .05 ** < .10 *
---
Note: If df < 4, do not trust the results
```

```
# check sensitivity to values of rho
sensitivity(FFTSMDCE)
```

```
RVE: Correlated Effects Model with Small-Sample Corrections 
Model: SMD ~ 1 

Sensitivity Analysis 

                          Rho = 0 Rho = 0.2 Rho = 0.4 Rho = 0.6 Rho = 0.8
 X.Intercept. Coefficient 0.0705  0.0708    0.0711    0.0714    0.0717   
              Std. Error  0.1071  0.1071    0.1072    0.1072    0.1072   
 Tau.sq       Estimate    0.0832  0.0840    0.0847    0.0855    0.0862   
 Rho = 1
 0.072  
 0.107  
 0.087
```

```
# compute the 95% prediction interval
lbPI <- FFTSMDCE$reg_table$b.r - 1.96*sqrt(FFTSMDCE$mod_info$tau.sq)
ubPI <- FFTSMDCE$reg_table$b.r + 1.96*sqrt(FFTSMDCE$mod_info$tau.sq)

#Lower bound
lbPI
```

```
           [,1]
[1,] -0.5038639
```

```
# Upper bound
ubPI
```

```
          [,1]
[1,] 0.6472643
```

## LOR Analysis: Overall Mean

The code below uses robumeta to estimate the mean log-odds ratio and its standard error using RVE. The sensitivity to assumptions about rho is also produced. There is a large outlier for study 49, effect ID 16 - there are 2 events out of 107 in the FFT group, and 18 out of 48 events in the control group. A second analysis is provided without this outlier. Sensitivity to assumptions about the correlation among effect sizes is computed and 95% prediction intervals provided.

```
# read in the data
fftES <- read.csv("FFTES.csv")

# use robumeta to compute the mean LOR with RVE

FFTLORCE <- robu(formula = LOR ~ 1, data = fftES,
                 studynum = StudyID, var.eff.size = varLOR,
                 rho = 0.8, small = TRUE)
FFTLORCE
```

```
RVE: Correlated Effects Model with Small-Sample Corrections 

Model: LOR ~ 1 

Number of studies = 9 
Number of outcomes = 59 (min = 1 , mean = 6.56 , median = 4 , max = 25 )
Rho = 0.8 
I.sq = 72.62958 
Tau.sq = 0.282326 

               Estimate StdErr t-value  dfs P(|t|>) 95% CI.L 95% CI.U Sig
1 X.Intercept.    0.331  0.233    1.43 7.22   0.196   -0.215    0.878    
---
Signif. codes: < .01 *** < .05 ** < .10 *
---
Note: If df < 4, do not trust the results
```

```
# check sensitivity to values of rho
sensitivity(FFTLORCE)
```

```
RVE: Correlated Effects Model with Small-Sample Corrections 
Model: LOR ~ 1 

Sensitivity Analysis 

                          Rho = 0 Rho = 0.2 Rho = 0.4 Rho = 0.6 Rho = 0.8
 X.Intercept. Coefficient 0.330   0.330     0.331     0.331     0.331    
              Std. Error  0.232   0.232     0.232     0.232     0.233    
 Tau.sq       Estimate    0.277   0.278     0.279     0.281     0.282    
 Rho = 1
 0.332  
 0.233  
 0.284
```

```
# convert results to odds ratio metric
# mean odds ratio
exp(FFTLORCE$b.r)
```

```
         [,1]
[1,] 1.392904
```

```
# 95% lower bound
exp(FFTLORCE$reg_table$CI.L)
```

```
[1] 0.806394
```

```
# 95% upper bound
exp(FFTLORCE$reg_table$CI.U)
```

```
[1] 2.405996
```

```
# compute the 95% prediction interval in log-odds metric
lbPIlor <- FFTLORCE$reg_table$b.r - 1.96*sqrt(FFTLORCE$mod_info$tau.sq)
ubPIlor <- FFTLORCE$reg_table$b.r + 1.96*sqrt(FFTLORCE$mod_info$tau.sq)

# mean LOR in odds ratio metric
exp(FFTLORCE$reg_table$b.r)
```

```
[1] 1.392904
```

```
# SE LOR in odds ratio metric
exp(FFTLORCE$reg_table$SE)
```

```
[1] 1.261816
```

```
#Lower bound
exp(lbPIlor)
```

```
          [,1]
[1,] 0.4916231
```

```
# Upper bound
exp(ubPIlor)
```

```
        [,1]
[1,] 3.94648
```

```
# Estimate the overall LOR without outlier from Study #49
fftES2 <- subset(fftES, fftES$LOR < 3)

# use robumeta to compute the mean LOR with RVE without outlier

FFTLORCE2 <- robu(formula = LOR ~ 1, data = fftES2,
                 studynum = StudyID, var.eff.size = varLOR,
                 rho = 0.8, small = TRUE)
FFTLORCE2
```

```
RVE: Correlated Effects Model with Small-Sample Corrections 

Model: LOR ~ 1 

Number of studies = 9 
Number of outcomes = 58 (min = 1 , mean = 6.44 , median = 4 , max = 25 )
Rho = 0.8 
I.sq = 70.09795 
Tau.sq = 0.2481298 

               Estimate StdErr t-value  dfs P(|t|>) 95% CI.L 95% CI.U Sig
1 X.Intercept.    0.304  0.223    1.36 7.11   0.215   -0.223    0.831    
---
Signif. codes: < .01 *** < .05 ** < .10 *
---
Note: If df < 4, do not trust the results
```

```
# check sensitivity to values of rho
sensitivity(FFTLORCE2)
```

```
RVE: Correlated Effects Model with Small-Sample Corrections 
Model: LOR ~ 1 

Sensitivity Analysis 

                          Rho = 0 Rho = 0.2 Rho = 0.4 Rho = 0.6 Rho = 0.8
 X.Intercept. Coefficient 0.303   0.303     0.303     0.304     0.304    
              Std. Error  0.223   0.223     0.223     0.223     0.223    
 Tau.sq       Estimate    0.242   0.244     0.245     0.247     0.248    
 Rho = 1
 0.304  
 0.224  
 0.250
```

```
# convert results to odds ratio metric
# mean odds ratio
exp(FFTLORCE2$b.r)
```

```
         [,1]
[1,] 1.355206
```

```
# 95% lower bound
exp(FFTLORCE2$reg_table$CI.L)
```

```
[1] 0.800315
```

```
# 95% upper bound
exp(FFTLORCE2$reg_table$CI.U)
```

```
[1] 2.294825
```

```
# compute the 95% prediction interval in log-odds metric
lbPIlor2 <- FFTLORCE2$reg_table$b.r - 1.96*sqrt(FFTLORCE2$mod_info$tau.sq)
ubPIlor2 <- FFTLORCE2$reg_table$b.r + 1.96*sqrt(FFTLORCE2$mod_info$tau.sq)

# mean LOR in odds ratio metric
exp(FFTLORCE2$reg_table$b.r)
```

```
[1] 1.355206
```

```
# SE LOR in odds ratio metric
exp(FFTLORCE2$reg_table$SE)
```

```
[1] 1.250336
```

```
#Lower bound
exp(lbPIlor2)
```

```
          [,1]
[1,] 0.5104951
```

```
# Upper bound
exp(ubPIlor2)
```

```
        [,1]
[1,] 3.59765
```

## SMD Analysis by Outcome

The code chunks below compute the mean effect size by outcome type for the studies reporting SMDs.

### SMD Placement Outcomes

This section estimates the mean SMD placement outcomes. Note that there are only 2 studies here with 7 effect sizes. The degrees of freedom in this analysis using RVE are less than 4. There is little guidance about how to handle the case of multiple effect sizes within a small number of studies. I also provide a fixed effects analysis with study as a moderator using metafor. Study 47 has estimates that are, on average, larger than those for Study 20. A combined analysis with LOR ES is provided at the end of the document.

```
# subset the data by SMD and LOR
FFTSMD <- subset(fftES, fftES$continuous == 1)
FFTLOR <- subset(fftES, fftES$continuous == 0)

# frequencies for each type of outcome
table(FFTSMD$Outcome)
```

```
 1  2  3  4  5  6  7  8  9 
 7  4 39 16 15 75 24 62  1
```

```
### PLACEMENT OUTCOMES
# subset to placement SMDs
SMDplacement <- subset(FFTSMD, FFTSMD$Outcome == 1)

# use robumeta to estimate the mean effect size

SMDPlCE <- robu(formula = SMD ~ 1, data = SMDplacement,
                 studynum = StudyID, var.eff.size = varsmd,
                 rho = 0.8, small = TRUE)
SMDPlCE
```

```
RVE: Correlated Effects Model with Small-Sample Corrections 

Model: SMD ~ 1 

Number of studies = 2 
Number of outcomes = 7 (min = 3 , mean = 3.5 , median = 3.5 , max = 4 )
Rho = 0.8 
I.sq = 62.44374 
Tau.sq = 0.105446 

               Estimate StdErr t-value dfs P(|t|>) 95% CI.L 95% CI.U Sig
1 X.Intercept.   0.0407  0.226    0.18   1   0.887    -2.84     2.92    
---
Signif. codes: < .01 *** < .05 ** < .10 *
---
Note: If df < 4, do not trust the results
```

```
# check sensitivity to values of rho
sensitivity(SMDPlCE)
```

```
RVE: Correlated Effects Model with Small-Sample Corrections 
Model: SMD ~ 1 

Sensitivity Analysis 

                          Rho = 0 Rho = 0.2 Rho = 0.4 Rho = 0.6 Rho = 0.8
 X.Intercept. Coefficient 0.0489  0.0465    0.0444    0.0425    0.0407   
              Std. Error  0.2250  0.2255    0.2258    0.2262    0.2264   
 Tau.sq       Estimate    0.0747  0.0824    0.0901    0.0978    0.1054   
 Rho = 1
 0.0392 
 0.2267 
 0.1131
```

```
# compute the 95% prediction interval
lbPIpl <- SMDPlCE$reg_table$b.r - 1.96*sqrt(SMDPlCE$mod_info$tau.sq)
ubPIpl <- SMDPlCE$reg_table$b.r + 1.96*sqrt(SMDPlCE$mod_info$tau.sq)

#Lower bound
lbPIpl
```

```
           [,1]
[1,] -0.5957237
```

```
# Upper bound
ubPIpl
```

```
          [,1]
[1,] 0.6771965
```

```
# use metafor to conduct a fixed effects analysis with
# study as a moderator

SMDPlrma <- rma(yi = SMD,
                vi = varsmd,
                data = SMDplacement, 
                mods = ~ -1 + factor(StudyID),
                method = "FE")

SMDPlrma
```

```
Fixed-Effects with Moderators Model (k = 7)

I^2 (residual heterogeneity / unaccounted variability): 0.00%
H^2 (unaccounted variability / sampling variability):   0.72

Test for Residual Heterogeneity:
QE(df = 5) = 3.5980, p-val = 0.6086

Test of Moderators (coefficients 1:2):
QM(df = 2) = 7.7311, p-val = 0.0210

Model Results:

                   estimate      se     zval    pval    ci.lb   ci.ub    
factor(StudyID)20   -0.2242  0.1414  -1.5856  0.1128  -0.5013  0.0529    
factor(StudyID)47    0.2338  0.1024   2.2841  0.0224   0.0332  0.4344  * 

---
Signif. codes:  0 '***' 0.001 '**' 0.01 '*' 0.05 '.' 0.1 ' ' 1
```

```
# forest plot to look at these seven ES
forest(SMDPlrma, slab = SMDplacement$StudyID)
```

### Arrest SMD outcomes

Below is the analysis for the 4 SMDs for the outcome, arrest. There are 2 studies and 4 effect sizes. The results of a fixed-effects moderator analysis is provided, comparing the mean effect sizes for the two different studies. A combined analysis with the LOR outcomes is provided at the end of the document.

```
### ARREST OUTCOMES
# subset to arrest SMDs
SMDarrest <- subset(FFTSMD, FFTSMD$Outcome == 2)

# use robumeta to estimate the mean effect size

SMDArCE <- robu(formula = SMD ~ 1, data = SMDarrest,
                 studynum = StudyID, var.eff.size = varsmd,
                 rho = 0.8, small = TRUE)
SMDArCE
```

```
RVE: Correlated Effects Model with Small-Sample Corrections 

Model: SMD ~ 1 

Number of studies = 2 
Number of outcomes = 4 (min = 1 , mean = 2 , median = 2 , max = 3 )
Rho = 0.8 
I.sq = 74.01373 
Tau.sq = 0.07512591 

               Estimate StdErr t-value dfs P(|t|>) 95% CI.L 95% CI.U Sig
1 X.Intercept.   -0.105  0.224   -0.47   1    0.72    -2.95     2.74    
---
Signif. codes: < .01 *** < .05 ** < .10 *
---
Note: If df < 4, do not trust the results
```

```
# check sensitivity to values of rho
sensitivity(SMDArCE)
```

```
RVE: Correlated Effects Model with Small-Sample Corrections 
Model: SMD ~ 1 

Sensitivity Analysis 

                          Rho = 0 Rho = 0.2 Rho = 0.4 Rho = 0.6 Rho = 0.8
 X.Intercept. Coefficient -0.106  -0.1059   -0.1057   -0.1055   -0.1053  
              Std. Error   0.224   0.2239    0.2239    0.2239    0.2239  
 Tau.sq       Estimate     0.070   0.0713    0.0726    0.0739    0.0751  
 Rho = 1
 -0.1052
  0.2239
  0.0764
```

```
# compute the 95% prediction interval
lbPIar <- SMDArCE$reg_table$b.r - 1.96*sqrt(SMDArCE$mod_info$tau.sq)
ubPIar <- SMDArCE$reg_table$b.r + 1.96*sqrt(SMDArCE$mod_info$tau.sq)

#Lower bound
lbPIar
```

```
           [,1]
[1,] -0.6425578
```

```
# Upper bound
ubPIar
```

```
          [,1]
[1,] 0.4318792
```

```
# use metafor to conduct a fixed effects analysis with
# study as a moderator

SMDARrma <- rma(yi = SMD,
                vi = varsmd,
                data = SMDarrest, 
                mods = ~ -1 + factor(StudyID),
                method = "FE")

SMDARrma
```

```
Fixed-Effects with Moderators Model (k = 4)

I^2 (residual heterogeneity / unaccounted variability): 0.00%
H^2 (unaccounted variability / sampling variability):   0.05

Test for Residual Heterogeneity:
QE(df = 2) = 0.1036, p-val = 0.9495

Test of Moderators (coefficients 1:2):
QM(df = 2) = 6.9357, p-val = 0.0312

Model Results:

                   estimate      se     zval    pval    ci.lb    ci.ub    
factor(StudyID)24   -0.3161  0.1380  -2.2903  0.0220  -0.5866  -0.0456  * 
factor(StudyID)47    0.1325  0.1019   1.3000  0.1936  -0.0673   0.3323    

---
Signif. codes:  0 '***' 0.001 '**' 0.01 '*' 0.05 '.' 0.1 ' ' 1
```

```
# forest plot to look at these  ES
forest(SMDARrma, slab = SMDarrest$StudyID)
```

### Substance Abuse SMD outcomes

The first analysis uses RVE with a correlated effects model. Though there are four studies and 39 ES, the dfs less than 3. The second analysis uses a fixed effects model with study as a moderator, and treating all studies as independent - this analysis is conducted to produce a forest plot of the ES. The third analysis aggregates the ES to the level of the study, and estimates the fixed effects mean across the four studies. We assume a correlation of 0.8 when aggregating effect sizes to the study level. The forest plot is also provided for this analysis. I provide these three analyses since there are no additional ES in the combined analysis.

```
### SUBSTANCE ABUSE OUTCOMES
# subset to substance abuse SMDs
SMDsubstance <- subset(FFTSMD, FFTSMD$Outcome == 3)

# use robumeta to estimate the mean effect size

SMDSACE <- robu(formula = SMD ~ 1, data = SMDsubstance,
                 studynum = StudyID, var.eff.size = varsmd,
                 rho = 0.8, small = TRUE)
SMDSACE
```

```
RVE: Correlated Effects Model with Small-Sample Corrections 

Model: SMD ~ 1 

Number of studies = 4 
Number of outcomes = 39 (min = 2 , mean = 9.75 , median = 4.5 , max = 28 )
Rho = 0.8 
I.sq = 68.59937 
Tau.sq = 0.1475241 

               Estimate StdErr t-value  dfs P(|t|>) 95% CI.L 95% CI.U Sig
1 X.Intercept.    0.338  0.191    1.78 2.96   0.175   -0.272    0.949    
---
Signif. codes: < .01 *** < .05 ** < .10 *
---
Note: If df < 4, do not trust the results
```

```
# check sensitivity to values of rho
sensitivity(SMDSACE)
```

```
RVE: Correlated Effects Model with Small-Sample Corrections 
Model: SMD ~ 1 

Sensitivity Analysis 

                          Rho = 0 Rho = 0.2 Rho = 0.4 Rho = 0.6 Rho = 0.8
 X.Intercept. Coefficient 0.337   0.337     0.338     0.338     0.338    
              Std. Error  0.190   0.190     0.190     0.190     0.191    
 Tau.sq       Estimate    0.134   0.138     0.141     0.144     0.148    
 Rho = 1
 0.339  
 0.191  
 0.151
```

```
# compute the 95% prediction interval
lbPIsa <- SMDSACE$reg_table$b.r - 1.96*sqrt(SMDSACE$mod_info$tau.sq)
ubPIsa <- SMDSACE$reg_table$b.r + 1.96*sqrt(SMDSACE$mod_info$tau.sq)

#Lower bound
lbPIsa
```

```
           [,1]
[1,] -0.4143301
```

```
# Upper bound
ubPIsa
```

```
         [,1]
[1,] 1.091297
```

```
# sort data by StudyID to facilitate the forest plot
SMDsubstance <- SMDsubstance[order(SMDsubstance$StudyID),]

# use metafor to conduct a fixed effects analysis with
# study as a moderator

SMDSArma <- rma(yi = SMD,
                vi = varsmd,
                data = SMDsubstance, 
                mods = ~ -1 + factor(StudyID),
                method = "FE")

SMDSArma
```

```
Fixed-Effects with Moderators Model (k = 39)

I^2 (residual heterogeneity / unaccounted variability): 1.58%
H^2 (unaccounted variability / sampling variability):   1.02

Test for Residual Heterogeneity:
QE(df = 35) = 35.5629, p-val = 0.4417

Test of Moderators (coefficients 1:4):
QM(df = 4) = 31.3298, p-val < .0001

Model Results:

                   estimate      se    zval    pval    ci.lb   ci.ub      
factor(StudyID)17    0.5038  0.1345  3.7460  0.0002   0.2402  0.7674  *** 
factor(StudyID)20    0.0646  0.0535  1.2078  0.2271  -0.0402  0.1694      
factor(StudyID)47    0.0268  0.0833  0.3221  0.7474  -0.1364  0.1901      
factor(StudyID)75    0.8351  0.2105  3.9667  <.0001   0.4225  1.2477  *** 

---
Signif. codes:  0 '***' 0.001 '**' 0.01 '*' 0.05 '.' 0.1 ' ' 1
```

```
# forest plot to look at these ES
forest(SMDSArma, slab = SMDsubstance$StudyID)
```

```
# Dfs for RVE are less than 4
# will use aggregate function in metafor to aggregate to level of study
# will assume a constant correlation of 0.8 among ES within studies

# turn substance data frame to an escalc object
SMDsubstance <- escalc(yi = SMD, vi = varsmd, data = SMDsubstance)

# average effect sizes
substanceagg <- aggregate(SMDsubstance, cluster = StudyID, rho = 0.8)

# use a fixed effects model to examine placement ES
substanceFE <- rma(yi = SMD,
               vi = varsmd,
               data = substanceagg,
               method = "FE")

substanceFE
```

```
Fixed-Effects Model (k = 4)

I^2 (total heterogeneity / total variability):   55.09%
H^2 (total variability / sampling variability):  2.23

Test for Heterogeneity:
Q(df = 3) = 6.6795, p-val = 0.0828

Model Results:

estimate      se    zval    pval   ci.lb   ci.ub    
  0.2730  0.1228  2.2237  0.0262  0.0324  0.5137  * 

---
Signif. codes:  0 '***' 0.001 '**' 0.01 '*' 0.05 '.' 0.1 ' ' 1
```

```
# forest plot for aggregated analysis
forest(substanceFE, slab = substanceagg$StudyID)
```

### Delinquency SMD outcomes

The first analysis uses RVE with a correlated effects model. Though there are five studies and 16 ES, the dfs less than 4. The second analysis uses a fixed effects model with study as a moderator, and treating all studies as independent - this analysis is conducted to produce a forest plot of the ES. The third analysis aggregates the ES to the level of the study (assuming a correlation among effect sizes within studies of 0.8), and estimates the fixed effects mean across the four studies. The forest plot is also provided for this analysis. I provide these analyses since there are no additional ES in the combined analysis.

```
### DELINQUENCY OUTCOMES

delinq <- subset(FFTSMD, FFTSMD$Outcome == 4)

# use robumeta to estimate the mean effect size

DeCE <- robu(formula = SMD ~ 1, data = delinq,
                 studynum = StudyID, var.eff.size = varsmd,
                 rho = 0.8, small = TRUE)
DeCE
```

```
RVE: Correlated Effects Model with Small-Sample Corrections 

Model: SMD ~ 1 

Number of studies = 5 
Number of outcomes = 16 (min = 2 , mean = 3.2 , median = 4 , max = 4 )
Rho = 0.8 
I.sq = 22.07914 
Tau.sq = 0.01466811 

               Estimate StdErr t-value  dfs P(|t|>) 95% CI.L 95% CI.U Sig
1 X.Intercept.  -0.0409 0.0921  -0.445 3.74   0.681   -0.304    0.222    
---
Signif. codes: < .01 *** < .05 ** < .10 *
---
Note: If df < 4, do not trust the results
```

```
# check sensitivity to values of rho
sensitivity(DeCE)
```

```
RVE: Correlated Effects Model with Small-Sample Corrections 
Model: SMD ~ 1 

Sensitivity Analysis 

                          Rho = 0  Rho = 0.2 Rho = 0.4 Rho = 0.6 Rho = 0.8
 X.Intercept. Coefficient -0.04267 -0.04221  -0.0418   -0.0413   -0.0409  
              Std. Error   0.09072  0.09109   0.0914    0.0918    0.0921  
 Tau.sq       Estimate     0.00839  0.00996   0.0115    0.0131    0.0147  
 Rho = 1
 -0.0405
  0.0924
  0.0162
```

```
# compute the 95% prediction interval
lbPIdelinq <- DeCE$reg_table$b.r - 1.96*sqrt(DeCE$mod_info$tau.sq)
ubPIdelinq <- DeCE$reg_table$b.r + 1.96*sqrt(DeCE$mod_info$tau.sq)

#Lower bound
lbPIdelinq
```

```
           [,1]
[1,] -0.2783209
```

```
# Upper bound
ubPIdelinq
```

```
          [,1]
[1,] 0.1964381
```

```
# sort data by StudyID to facilitate the forest plot
delinq <- delinq[order(delinq$StudyID),]

# use metafor to conduct a fixed effects analysis with
# study as a moderator

DErma <- rma(yi = SMD,
                vi = varsmd,
                data = delinq, 
                mods = ~ -1 + factor(StudyID),
                method = "FE")

DErma
```

```
Fixed-Effects with Moderators Model (k = 16)

I^2 (residual heterogeneity / unaccounted variability): 0.00%
H^2 (unaccounted variability / sampling variability):   0.56

Test for Residual Heterogeneity:
QE(df = 11) = 6.1739, p-val = 0.8615

Test of Moderators (coefficients 1:5):
QM(df = 5) = 10.5030, p-val = 0.0622

Model Results:

                   estimate      se     zval    pval    ci.lb    ci.ub    
factor(StudyID)17    0.3098  0.1324   2.3410  0.0192   0.0504   0.5692  * 
factor(StudyID)20   -0.0366  0.1414  -0.2588  0.7958  -0.3138   0.2406    
factor(StudyID)39    0.0189  0.1011   0.1874  0.8514  -0.1791   0.2170    
factor(StudyID)40   -0.3291  0.1528  -2.1532  0.0313  -0.6286  -0.0295  * 
factor(StudyID)47   -0.0695  0.1304  -0.5333  0.5938  -0.3251   0.1860    

---
Signif. codes:  0 '***' 0.001 '**' 0.01 '*' 0.05 '.' 0.1 ' ' 1
```

```
# forest plot to look at these seven ES
forest(DErma, slab = delinq$StudyID)
```

```
# Dfs for RVE are less than 4
# will use aggregate function in metafor to aggregate to level of study
# will assume a constant correlation of 0.8 among ES within studies

# turn delinquency data frame to an escalc object
delinq <- escalc(yi = SMD, vi = varsmd, data = delinq)

# average effect sizes
delinqagg <- aggregate(delinq, cluster = StudyID, rho = 0.8)

# use a fixed effects model to examine placement ES
delinqFE <- rma(yi = SMD,
               vi = varsmd,
               data = delinqagg,
               method = "FE")

delinqFE
```

```
Fixed-Effects Model (k = 5)

I^2 (total heterogeneity / total variability):   0.00%
H^2 (total variability / sampling variability):  0.92

Test for Heterogeneity:
Q(df = 4) = 3.6748, p-val = 0.4518

Model Results:

estimate      se     zval    pval    ci.lb   ci.ub    
 -0.0455  0.0990  -0.4595  0.6459  -0.2395  0.1485    

---
Signif. codes:  0 '***' 0.001 '**' 0.01 '*' 0.05 '.' 0.1 ' ' 1
```

```
# forest plot for aggregated analysis
forest(delinqFE, slab = delinqagg$StudyID)
```

### Peer Relations SMD outcomes

The first analysis uses RVE with a correlated effects model. Though there are three studies and 15 ES, the dfs are less than 4. The second analysis uses a fixed effects model with study as a moderator, and treating all studies as independent - this analysis is conducted to produce a forest plot of the ES. The third analysis aggregates the ES to the level of the study assuming a correlation among effect sizes within studies of 0.8, and estimates the fixed effects mean across the four studies. The forest plot is also provided for this analysis. These analyses are provided here since there are no additional ES in the combined analysis.

```
### PEER RELATIONS OUTCOMES
# subset to peer relations SMDs
SMDpeer <- subset(FFTSMD, FFTSMD$Outcome == 5)

# use robumeta to estimate the mean effect size

SMDPRCE <- robu(formula = SMD ~ 1, data = SMDpeer,
                 studynum = StudyID, var.eff.size = varsmd,
                 rho = 0.8, small = TRUE)
SMDPRCE
```

```
RVE: Correlated Effects Model with Small-Sample Corrections 

Model: SMD ~ 1 

Number of studies = 3 
Number of outcomes = 15 (min = 2 , mean = 5 , median = 3 , max = 10 )
Rho = 0.8 
I.sq = 59.19253 
Tau.sq = 0.106362 

               Estimate StdErr t-value  dfs P(|t|>) 95% CI.L 95% CI.U Sig
1 X.Intercept.   0.0273  0.203   0.134 1.56   0.908    -1.13     1.18    
---
Signif. codes: < .01 *** < .05 ** < .10 *
---
Note: If df < 4, do not trust the results
```

```
# check sensitivity to values of rho
sensitivity(SMDPRCE)
```

```
RVE: Correlated Effects Model with Small-Sample Corrections 
Model: SMD ~ 1 

Sensitivity Analysis 

                          Rho = 0 Rho = 0.2 Rho = 0.4 Rho = 0.6 Rho = 0.8
 X.Intercept. Coefficient 0.0165  0.0193    0.0220    0.0247    0.0273   
              Std. Error  0.1960  0.1978    0.1996    0.2013    0.2030   
 Tau.sq       Estimate    0.0871  0.0919    0.0967    0.1015    0.1064   
 Rho = 1
 0.0298 
 0.2047 
 0.1112
```

```
# compute the 95% prediction interval
lbPIpr <- SMDPRCE$reg_table$b.r - 1.96*sqrt(SMDPRCE$mod_info$tau.sq)
ubPIpr <- SMDPRCE$reg_table$b.r + 1.96*sqrt(SMDPRCE$mod_info$tau.sq)

#Lower bound
lbPIpr
```

```
           [,1]
[1,] -0.6119351
```

```
# Upper bound
ubPIpr
```

```
          [,1]
[1,] 0.6665016
```

```
# use metafor to conduct a fixed effects analysis with
# study as a moderator

SMDPRrma <- rma(yi = SMD,
                vi = varsmd,
                data = SMDpeer, 
                mods = ~ -1 + factor(StudyID),
                method = "FE")

SMDPRrma
```

```
Fixed-Effects with Moderators Model (k = 15)

I^2 (residual heterogeneity / unaccounted variability): 0.00%
H^2 (unaccounted variability / sampling variability):   0.42

Test for Residual Heterogeneity:
QE(df = 12) = 5.0141, p-val = 0.9575

Test of Moderators (coefficients 1:3):
QM(df = 3) = 22.4611, p-val < .0001

Model Results:

                   estimate      se     zval    pval    ci.lb    ci.ub      
factor(StudyID)5     0.8276  0.3495   2.3681  0.0179   0.1426   1.5126    * 
factor(StudyID)39   -0.2536  0.0619  -4.0989  <.0001  -0.3749  -0.1324  *** 
factor(StudyID)47    0.0322  0.1415   0.2276  0.8200  -0.2451   0.3094      

---
Signif. codes:  0 '***' 0.001 '**' 0.01 '*' 0.05 '.' 0.1 ' ' 1
```

```
# forest plot to look at these  ES
forest(SMDPRrma, slab = SMDpeer$StudyID)
```

```
# Dfs for RVE are less than 4
# will use aggregate function in metafor to aggregate to level of study
# will assume a constant correlation of 0.8 among ES within studies

# turn peer data frame to an escalc object
SMDpeer <- escalc(yi = SMD, vi = varsmd, data = SMDpeer)

# average effect sizes
peeragg <- aggregate(SMDpeer, cluster = StudyID, rho = 0.8)

# use a fixed effects model to examine placement ES
peerFE <- rma(yi = SMD,
               vi = varsmd,
               data = peeragg,
               method = "FE")

peerFE
```

```
Fixed-Effects Model (k = 3)

I^2 (total heterogeneity / total variability):   44.33%
H^2 (total variability / sampling variability):  1.80

Test for Heterogeneity:
Q(df = 2) = 3.5923, p-val = 0.1659

Model Results:

estimate      se     zval    pval    ci.lb   ci.ub    
 -0.0541  0.1368  -0.3954  0.6926  -0.3223  0.2141    

---
Signif. codes:  0 '***' 0.001 '**' 0.01 '*' 0.05 '.' 0.1 ' ' 1
```

```
# forest plot for aggregated analysis
forest(peerFE, slab = peeragg$StudyID)
```

### Youth Behavior Symptoms SMD outcomes

Below is the analysis for the youth behavior symptoms SMD outcomes. There are seven studies with 70 SMDs. The CE model is appropriate here. An additional analysis is provided at the end of the document that combines the SMD and LOR ES.

```
### YOUTH BEHAVIOR SYMPTOMS OUTCOMES
# subset to youth behavior symptoms SMDs
SMDyouth <- subset(FFTSMD, FFTSMD$Outcome == 6)

# use robumeta to estimate the mean effect size

SMDybCE <- robu(formula = SMD ~ 1, data = SMDyouth,
                 studynum = StudyID, var.eff.size = varsmd,
                 rho = 0.8, small = TRUE)
SMDybCE
```

```
RVE: Correlated Effects Model with Small-Sample Corrections 

Model: SMD ~ 1 

Number of studies = 7 
Number of outcomes = 70 (min = 3 , mean = 10 , median = 8 , max = 19 )
Rho = 0.8 
I.sq = 13.7759 
Tau.sq = 0.009346008 

               Estimate StdErr t-value  dfs P(|t|>) 95% CI.L 95% CI.U Sig
1 X.Intercept.  -0.0291 0.0647   -0.45 4.55   0.673     -0.2    0.142    
---
Signif. codes: < .01 *** < .05 ** < .10 *
---
Note: If df < 4, do not trust the results
```

```
# check sensitivity to values of rho
sensitivity(SMDybCE)
```

```
RVE: Correlated Effects Model with Small-Sample Corrections 
Model: SMD ~ 1 

Sensitivity Analysis 

                          Rho = 0  Rho = 0.2 Rho = 0.4 Rho = 0.6 Rho = 0.8
 X.Intercept. Coefficient -0.03057 -0.03021  -0.02984  -0.02947  -0.02909 
              Std. Error   0.06412  0.06427   0.06441   0.06455   0.06468 
 Tau.sq       Estimate     0.00338  0.00487   0.00636   0.00785   0.00935 
 Rho = 1
 -0.0287
  0.0648
  0.0108
```

```
# compute the 95% prediction interval
lbPIyb <- SMDybCE$reg_table$b.r - 1.96*sqrt(SMDybCE$mod_info$tau.sq)
ubPIyb <- SMDybCE$reg_table$b.r + 1.96*sqrt(SMDybCE$mod_info$tau.sq)

#Lower bound
lbPIyb
```

```
           [,1]
[1,] -0.2185746
```

```
# Upper bound
ubPIyb
```

```
          [,1]
[1,] 0.1603904
```

### Parent Behavior Symptoms SMD outcomes

Below is the analysis of parent behavior symptoms SMD outcomes. There are five studies with 22 SMDs. The estimate of tau-squared is 0 - so the 95% prediction interval does not make sense. The second analysis uses a fixed effects model with study as a moderator, and treating all studies as independent - this analysis is conducted to produce a forest plot of the 22 ES. The third analysis aggregates the ES to the level of the study assuming a correlation of 0.8 for effect sizes within studies, and estimates the fixed effects mean across the four studies. The forest plot is also provided for this analysis. These analyses are provided here as there are no additional ES in the combined analysis.

```
### PARENT BEHAVIOR SYMPTOMS OUTCOMES
# subset to parent behavior symptoms SMDs
SMDparent <- subset(FFTSMD, FFTSMD$Outcome == 7)

# use robumeta to estimate the mean effect size

SMDPBCE <- robu(formula = SMD ~ 1, data = SMDparent,
                 studynum = StudyID, var.eff.size = varsmd,
                 rho = 0.8, small = TRUE)
SMDPBCE
```

```
RVE: Correlated Effects Model with Small-Sample Corrections 

Model: SMD ~ 1 

Number of studies = 5 
Number of outcomes = 22 (min = 1 , mean = 4.4 , median = 5 , max = 8 )
Rho = 0.8 
I.sq = 0 
Tau.sq = 0 

               Estimate StdErr t-value  dfs P(|t|>) 95% CI.L 95% CI.U Sig
1 X.Intercept.  -0.0139 0.0644  -0.216 3.06   0.842   -0.216    0.189    
---
Signif. codes: < .01 *** < .05 ** < .10 *
---
Note: If df < 4, do not trust the results
```

```
# check sensitivity to values of rho
sensitivity(SMDPBCE)
```

```
RVE: Correlated Effects Model with Small-Sample Corrections 
Model: SMD ~ 1 

Sensitivity Analysis 

                          Rho = 0 Rho = 0.2 Rho = 0.4 Rho = 0.6 Rho = 0.8
 X.Intercept. Coefficient -0.0139 -0.0139   -0.0139   -0.0139   -0.0139  
              Std. Error   0.0644  0.0644    0.0644    0.0644    0.0644  
 Tau.sq       Estimate     0.0000  0.0000    0.0000    0.0000    0.0000  
 Rho = 1
 -0.0139
  0.0644
  0.0000
```

```
# compute the 95% prediction interval
lbPIpb <- SMDPBCE$reg_table$b.r - 1.96*sqrt(SMDPBCE$mod_info$tau.sq)
ubPIpb <- SMDPBCE$reg_table$b.r + 1.96*sqrt(SMDPBCE$mod_info$tau.sq)

#Lower bound
lbPIpb
```

```
            [,1]
[1,] -0.01392533
```

```
# Upper bound
ubPIpb
```

```
            [,1]
[1,] -0.01392533
```

```
# sort data by StudyID to facilitate the forest plot
SMDparent <- SMDparent[order(SMDparent$StudyID),]

# use metafor to conduct a fixed effects analysis with
# study as a moderator

SMDPArma <- rma(yi = SMD,
                vi = varsmd,
                data = SMDparent, 
                mods = ~ -1 + factor(StudyID),
                method = "FE")
```

```
Warning: Studies with NAs omitted from model fitting.
```

```
SMDPArma
```

```
Fixed-Effects with Moderators Model (k = 22)

I^2 (residual heterogeneity / unaccounted variability): 0.00%
H^2 (unaccounted variability / sampling variability):   0.84

Test for Residual Heterogeneity:
QE(df = 17) = 14.2603, p-val = 0.6486

Test of Moderators (coefficients 1:5):
QM(df = 5) = 5.0012, p-val = 0.4157

Model Results:

                   estimate      se     zval    pval    ci.lb    ci.ub    
factor(StudyID)19    0.1514  0.3857   0.3926  0.6946  -0.6045   0.9074    
factor(StudyID)20   -0.0443  0.0998  -0.4441  0.6570  -0.2399   0.1513    
factor(StudyID)40    0.0567  0.0956   0.5932  0.5531  -0.1307   0.2442    
factor(StudyID)47   -0.1680  0.0844  -1.9911  0.0465  -0.3333  -0.0026  * 
factor(StudyID)49    0.0710  0.1229   0.5776  0.5635  -0.1699   0.3118    

---
Signif. codes:  0 '***' 0.001 '**' 0.01 '*' 0.05 '.' 0.1 ' ' 1
```

```
# forest plot to look at these  ES
forest(SMDPArma, slab = SMDparent$StudyID)
```

```
# Dfs for RVE are less than 4
# will use aggregate function in metafor to aggregate to level of study
# will assume a constant correlation of 0.8 among ES within studies

# turn parent data frame to an escalc object
SMDparent <- escalc(yi = SMD, vi = varsmd, data = SMDparent)

# average effect sizes
parentagg <- aggregate(SMDparent, cluster = StudyID, rho = 0.8)

# use a fixed effects model to examine placement ES
parentFE <- rma(yi = SMD,
               vi = varsmd,
               data = parentagg,
               method = "FE")

parentFE
```

```
Fixed-Effects Model (k = 5)

I^2 (total heterogeneity / total variability):   0.00%
H^2 (total variability / sampling variability):  0.28

Test for Heterogeneity:
Q(df = 4) = 1.1313, p-val = 0.8893

Model Results:

estimate      se     zval    pval    ci.lb   ci.ub    
 -0.0139  0.1008  -0.1381  0.8901  -0.2115  0.1837    

---
Signif. codes:  0 '***' 0.001 '**' 0.01 '*' 0.05 '.' 0.1 ' ' 1
```

```
# forest plot for aggregated analysis
forest(parentFE, slab = parentagg$StudyID)
```

### Family Function SMD outcomes

Below is the analysis for the family functioning SMD outcomes. There are 5 studies and 60 SMDs. One study contributes 36 SMDs. Tau-squared cannot be estimated here as in the prior analysis. The second analysis uses a fixed effects model with study as a moderator, and treating all studies as independent - this analysis is conducted to produce study means. A forest plot of the 60 ES is not provided since it is unreadable. The third analysis aggregates the ES to the level of the study using a correlation of 0.8 for effect sizes within studies, and estimates the fixed effects mean across the four studies. The forest plot is also provided for this analysis.

```
SMDfamily <- subset(FFTSMD, FFTSMD$Outcome == 8)

# use robumeta to estimate the mean effect size

SMDFamCE <- robu(formula = SMD ~ 1, data = SMDfamily,
                 studynum = StudyID, var.eff.size = varsmd,
                 rho = 0.8, small = TRUE)
SMDFamCE
```

```
RVE: Correlated Effects Model with Small-Sample Corrections 

Model: SMD ~ 1 

Number of studies = 5 
Number of outcomes = 60 (min = 2 , mean = 12 , median = 4 , max = 36 )
Rho = 0.8 
I.sq = 0 
Tau.sq = 0 

               Estimate StdErr t-value dfs P(|t|>) 95% CI.L 95% CI.U Sig
1 X.Intercept.    0.137  0.033    4.14 2.4  0.0389   0.0151    0.259  **
---
Signif. codes: < .01 *** < .05 ** < .10 *
---
Note: If df < 4, do not trust the results
```

```
# check sensitivity to values of rho
sensitivity(SMDFamCE)
```

```
RVE: Correlated Effects Model with Small-Sample Corrections 
Model: SMD ~ 1 

Sensitivity Analysis 

                          Rho = 0 Rho = 0.2 Rho = 0.4 Rho = 0.6 Rho = 0.8
 X.Intercept. Coefficient 0.137   0.137     0.137     0.137     0.137    
              Std. Error  0.033   0.033     0.033     0.033     0.033    
 Tau.sq       Estimate    0.000   0.000     0.000     0.000     0.000    
 Rho = 1
 0.137  
 0.033  
 0.000
```

```
# compute the 95% prediction interval
lbPIfam <- SMDFamCE$reg_table$b.r - 1.96*sqrt(SMDFamCE$mod_info$tau.sq)
ubPIfam <- SMDFamCE$reg_table$b.r + 1.96*sqrt(SMDFamCE$mod_info$tau.sq)

#Lower bound
lbPIfam
```

```
          [,1]
[1,] 0.1369242
```

```
# Upper bound
ubPIfam
```

```
          [,1]
[1,] 0.1369242
```

```
# use metafor to conduct a fixed effects analysis with
# study as a moderator

SMDfamilyrma <- rma(yi = SMD,
                vi = varsmd,
                data = SMDfamily, 
                mods = ~ -1 + factor(StudyID),
                method = "FE")
```

```
Warning: Studies with NAs omitted from model fitting.
```

```
SMDfamilyrma
```

```
Fixed-Effects with Moderators Model (k = 60)

I^2 (residual heterogeneity / unaccounted variability): 0.00%
H^2 (unaccounted variability / sampling variability):   0.54

Test for Residual Heterogeneity:
QE(df = 55) = 29.6770, p-val = 0.9979

Test of Moderators (coefficients 1:5):
QM(df = 5) = 40.2967, p-val < .0001

Model Results:

                   estimate      se    zval    pval    ci.lb   ci.ub      
factor(StudyID)5     0.5794  0.0993  5.8341  <.0001   0.3848  0.7741  *** 
factor(StudyID)19    0.1338  0.1806  0.7409  0.4588  -0.2202  0.4877      
factor(StudyID)20    0.1406  0.0707  1.9892  0.0467   0.0021  0.2792    * 
factor(StudyID)47    0.0686  0.1316  0.5210  0.6024  -0.1894  0.3265      
factor(StudyID)49    0.1497  0.1230  1.2174  0.2234  -0.0913  0.3908      

---
Signif. codes:  0 '***' 0.001 '**' 0.01 '*' 0.05 '.' 0.1 ' ' 1
```

```
# Dfs for RVE are less than 4
# will use aggregate function in metafor to aggregate to level of study
# will assume a constant correlation of 0.8 among ES within studies

# turn parent data frame to an escalc object
SMDfamily <- escalc(yi = SMD, vi = varsmd, data = SMDfamily)

# average effect sizes
familyagg <- aggregate(SMDfamily, cluster = StudyID, rho = 0.8)

# use a fixed effects model to examine placement ES
familyFE <- rma(yi = SMD,
               vi = varsmd,
               data = familyagg,
               method = "FE")

familyFE
```

```
Fixed-Effects Model (k = 5)

I^2 (total heterogeneity / total variability):   0.00%
H^2 (total variability / sampling variability):  0.19

Test for Heterogeneity:
Q(df = 4) = 0.7520, p-val = 0.9448

Model Results:

estimate      se    zval    pval    ci.lb   ci.ub    
  0.1369  0.1086  1.2605  0.2075  -0.0760  0.3498    

---
Signif. codes:  0 '***' 0.001 '**' 0.01 '*' 0.05 '.' 0.1 ' ' 1
```

```
# forest plot for aggregated analysis
forest(familyFE, slab = familyagg$StudyID)
```

### School SMD outcomes

There is only one effect size for school SMD outcomes. No analysis is possible.

```
SMDschool <- subset(FFTSMD, FFTSMD$Outcome == 9)

summary(SMDschool$SMD)
```

```
   Min. 1st Qu.  Median    Mean 3rd Qu.    Max. 
-0.1292 -0.1292 -0.1292 -0.1292 -0.1292 -0.1292
```

## LOR Analysis by outcome

The code chunks below compute the mean effect size by outcome type for the studies reporting LORs. There are sufficient outcomes for placement (1), arrest (2), youth behavior symptoms (6), and school (9).

```
# get frequencies for number of types of outcomes for LOR
table(FFTLOR$Outcome)
```

```
 1  2  5  6  9 
12 44  1  4  2
```

### Placement LOR outcomes

Below is the analysis for placement LOR outcomes. There are three studies and 11 outcomes. Since the dfs in the RVE analysis are less than 4, I add a fixed effects analysis with Study ID as a moderator. An additional analysis with combined SMD and LOR outcomes is provided at the end of the document.

```
# PLACEMENT LOR OUTCOMES
# subset to placement lorS
LORplacement <- subset(FFTLOR, FFTLOR$Outcome == 1)

# use robumeta to compute the mean LOR with RVE

FFTPlLCE <- robu(formula = LOR ~ 1, data = LORplacement,
                 studynum = StudyID, var.eff.size = varLOR,
                 rho = 0.8, small = TRUE)
FFTPlLCE
```

```
RVE: Correlated Effects Model with Small-Sample Corrections 

Model: LOR ~ 1 

Number of studies = 3 
Number of outcomes = 11 (min = 2 , mean = 3.67 , median = 4 , max = 5 )
Rho = 0.8 
I.sq = 38.7378 
Tau.sq = 0.1376557 

               Estimate StdErr t-value  dfs P(|t|>) 95% CI.L 95% CI.U Sig
1 X.Intercept.    0.374  0.154    2.43 1.91   0.141   -0.318     1.07    
---
Signif. codes: < .01 *** < .05 ** < .10 *
---
Note: If df < 4, do not trust the results
```

```
# check sensitivity to values of rho
sensitivity(FFTPlLCE)
```

```
RVE: Correlated Effects Model with Small-Sample Corrections 
Model: LOR ~ 1 

Sensitivity Analysis 

                          Rho = 0 Rho = 0.2 Rho = 0.4 Rho = 0.6 Rho = 0.8
 X.Intercept. Coefficient 0.3680  0.3698    0.371     0.373     0.374    
              Std. Error  0.1574  0.1564    0.155     0.155     0.154    
 Tau.sq       Estimate    0.0822  0.0961    0.110     0.124     0.138    
 Rho = 1
 0.375  
 0.153  
 0.152
```

```
# convert results to odds ratio metric
# mean odds ratio
exp(FFTPlLCE$b.r)
```

```
        [,1]
[1,] 1.45378
```

```
# SE of odds ratio
exp(FFTPlLCE$reg_table$SE)
```

```
[1] 1.166294
```

```
# 95% lower bound
exp(FFTPlLCE$reg_table$CI.L)
```

```
[1] 0.7275072
```

```
# 95% upper bound
exp(FFTPlLCE$reg_table$CI.U)
```

```
[1] 2.905092
```

```
# compute the 95% prediction interval in log-odds metric
lbPllor <- FFTPlLCE$reg_table$b.r - 1.96*sqrt(FFTPlLCE$mod_info$tau.sq)
ubPllor <- FFTPlLCE$reg_table$b.r + 1.96*sqrt(FFTPlLCE$mod_info$tau.sq)

# mean LOR in odds ratio metric
exp(FFTPlLCE$reg_table$b.r)
```

```
[1] 1.45378
```

```
# SE LOR in odds ratio metric
exp(FFTPlLCE$reg_table$SE)
```

```
[1] 1.166294
```

```
#Lower bound
exp(lbPllor)
```

```
          [,1]
[1,] 0.7025547
```

```
# Upper bound
exp(ubPllor)
```

```
         [,1]
[1,] 3.008271
```

```
# sort data by StudyID to facilitate the forest plot
LORplacement <- LORplacement[order(LORplacement$StudyID),]

# use metafor to conduct a fixed effects analysis with
# study as a moderator

LORPlrma <- rma(yi = LOR,
                vi = varLOR,
                data = LORplacement, 
                mods = ~ -1 + factor(StudyID),
                method = "FE")
```

```
Warning: Studies with NAs omitted from model fitting.
```

```
Warning: Redundant predictors dropped from the model.
```

```
LORPlrma
```

```
Fixed-Effects with Moderators Model (k = 11)

I^2 (residual heterogeneity / unaccounted variability): 45.79%
H^2 (unaccounted variability / sampling variability):   1.84

Test for Residual Heterogeneity:
QE(df = 8) = 14.7577, p-val = 0.0640

Test of Moderators (coefficients 1:3):
QM(df = 3) = 32.8169, p-val < .0001

Model Results:

                   estimate      se    zval    pval    ci.lb   ci.ub      
factor(StudyID)47    0.1409  0.1838  0.7666  0.4433  -0.2194  0.5012      
factor(StudyID)49    0.5286  0.3126  1.6911  0.0908  -0.0840  1.1413    . 
factor(StudyID)50    0.9539  0.1760  5.4193  <.0001   0.6089  1.2989  *** 

---
Signif. codes:  0 '***' 0.001 '**' 0.01 '*' 0.05 '.' 0.1 ' ' 1
```

```
# transform the means to OR
exp(LORPlrma$beta)
```

```
                      [,1]
factor(StudyID)47 1.151337
factor(StudyID)49 1.696573
factor(StudyID)50 2.595877
```

```
# transform the SEs to OR metric
exp(LORPlrma$se)
```

```
[1] 1.201819 1.366948 1.192464
```

```
# transform the lower bound to OR metric
exp(LORPlrma$ci.lb)
```

```
[1] 0.8030097 0.9193986 1.8384588
```

```
# transform the upper bound to OR metric
exp(LORPlrma$ci.ub)
```

```
[1] 1.650759 3.130700 3.665340
```

```
# forest plot of ORs
forest(LORPlrma, slab = LORplacement$StudyID, transf = exp, refline = 1 )
```

### Arrest LOR outcomes

Below is the analysis for the Arrest LOR outcomes. There are 8 studies and 43 LORs. A second analysis is provided without the outlier in study #49 (outcome 16). An additional analysis with combined SMD and LOR outcomes is provided at the end of the document.

```
# ARREST LOR OUTCOMES
# subset to arrest lorS
LORarrest <- subset(FFTLOR, FFTLOR$Outcome == 2)

# summary statistics for LOR for arrest outcomes
summary(FFTLOR$LOR)
```

```
    Min.  1st Qu.   Median     Mean  3rd Qu.     Max.     NA's 
-1.06031 -0.04196  0.31449  0.43606  0.83371  3.44999        4
```

```
# use robumeta to compute the mean LOR with RVE

FFTArLCE <- robu(formula = LOR ~ 1, data = LORarrest,
                 studynum = StudyID, var.eff.size = varLOR,
                 rho = 0.8, small = TRUE)
FFTArLCE
```

```
RVE: Correlated Effects Model with Small-Sample Corrections 

Model: LOR ~ 1 

Number of studies = 8 
Number of outcomes = 42 (min = 1 , mean = 5.25 , median = 2.5 , max = 19 )
Rho = 0.8 
I.sq = 73.04564 
Tau.sq = 0.266689 

               Estimate StdErr t-value  dfs P(|t|>) 95% CI.L 95% CI.U Sig
1 X.Intercept.    0.346  0.248    1.39 6.26   0.211   -0.255    0.948    
---
Signif. codes: < .01 *** < .05 ** < .10 *
---
Note: If df < 4, do not trust the results
```

```
# check sensitivity to values of rho
sensitivity(FFTArLCE)
```

```
RVE: Correlated Effects Model with Small-Sample Corrections 
Model: LOR ~ 1 

Sensitivity Analysis 

                          Rho = 0 Rho = 0.2 Rho = 0.4 Rho = 0.6 Rho = 0.8
 X.Intercept. Coefficient 0.345   0.345     0.346     0.346     0.346    
              Std. Error  0.248   0.248     0.248     0.248     0.248    
 Tau.sq       Estimate    0.261   0.262     0.264     0.265     0.267    
 Rho = 1
 0.347  
 0.249  
 0.268
```

```
# convert results to odds ratio metric
# mean odds ratio
exp(FFTArLCE$b.r)
```

```
         [,1]
[1,] 1.414097
```

```
# SE for odds ratio
exp(FFTArLCE$reg_table$SE)
```

```
[1] 1.281993
```

```
# 95% lower bound
exp(FFTArLCE$reg_table$CI.L)
```

```
[1] 0.7746823
```

```
# 95% upper bound
exp(FFTArLCE$reg_table$CI.U)
```

```
[1] 2.581279
```

```
# compute the 95% prediction interval in log-odds metric
lbArlor <- FFTArLCE$reg_table$b.r - 1.96*sqrt(FFTArLCE$mod_info$tau.sq)
ubArlor <- FFTArLCE$reg_table$b.r + 1.96*sqrt(FFTArLCE$mod_info$tau.sq)

# mean LOR in odds ratio metric
exp(FFTArLCE$reg_table$b.r)
```

```
[1] 1.414097
```

```
#Lower bound
exp(lbArlor)
```

```
          [,1]
[1,] 0.5139184
```

```
# Upper bound
exp(ubArlor)
```

```
         [,1]
[1,] 3.891028
```

```
## ANALYSIS WITHOUT THE OUTLIER
LORarrest2 <- subset(LORarrest, LORarrest$LOR < 3.0)

# use robumeta to compute the mean LOR with RVE

FFTArLCE2 <- robu(formula = LOR ~ 1, data = LORarrest2,
                 studynum = StudyID, var.eff.size = varLOR,
                 rho = 0.8, small = TRUE)
FFTArLCE2
```

```
RVE: Correlated Effects Model with Small-Sample Corrections 

Model: LOR ~ 1 

Number of studies = 8 
Number of outcomes = 41 (min = 1 , mean = 5.12 , median = 2.5 , max = 19 )
Rho = 0.8 
I.sq = 69.88672 
Tau.sq = 0.2271652 

               Estimate StdErr t-value  dfs P(|t|>) 95% CI.L 95% CI.U Sig
1 X.Intercept.    0.311  0.235    1.32 6.14   0.234   -0.262    0.884    
---
Signif. codes: < .01 *** < .05 ** < .10 *
---
Note: If df < 4, do not trust the results
```

```
# check sensitivity to values of rho
sensitivity(FFTArLCE2)
```

```
RVE: Correlated Effects Model with Small-Sample Corrections 
Model: LOR ~ 1 

Sensitivity Analysis 

                          Rho = 0 Rho = 0.2 Rho = 0.4 Rho = 0.6 Rho = 0.8
 X.Intercept. Coefficient 0.309   0.309     0.310     0.310     0.311    
              Std. Error  0.235   0.235     0.235     0.235     0.235    
 Tau.sq       Estimate    0.221   0.223     0.224     0.226     0.227    
 Rho = 1
 0.311  
 0.236  
 0.229
```

```
# convert results to odds ratio metric
# mean odds ratio
exp(FFTArLCE2$b.r)
```

```
         [,1]
[1,] 1.364443
```

```
# SE for odds ratio
exp(FFTArLCE2$reg_table$SE)
```

```
[1] 1.26537
```

```
# 95% lower bound
exp(FFTArLCE2$reg_table$CI.L)
```

```
[1] 0.7694735
```

```
# 95% upper bound
exp(FFTArLCE2$reg_table$CI.U)
```

```
[1] 2.419454
```

```
# compute the 95% prediction interval in log-odds metric
lbArlor2 <- FFTArLCE2$reg_table$b.r - 1.96*sqrt(FFTArLCE2$mod_info$tau.sq)
ubArlor2 <- FFTArLCE2$reg_table$b.r + 1.96*sqrt(FFTArLCE2$mod_info$tau.sq)

# mean LOR in odds ratio metric
exp(FFTArLCE2$reg_table$b.r)
```

```
[1] 1.364443
```

```
#Lower bound
exp(lbArlor2)
```

```
          [,1]
[1,] 0.5361047
```

```
# Upper bound
exp(ubArlor2)
```

```
         [,1]
[1,] 3.472652
```

### Youth Behavior Symptoms

Below is the analysis for youth behavior symptoms for LOR outcomes. Since there is only one study, I use metafor to estimate the fixed effect analysis, and then convert to the odds ratio metric. An additional analysis is provided at the end of the document for the combined ES.

```
# Youth Behavior Symptoms LOR OUTCOMES
# subset to youth behavior lorS
LORyouth <- subset(FFTLOR, FFTLOR$Outcome == 6)

# Examine the number of outcomes and studies
table(LORyouth$StudyID)
```

```
40 
 4
```

```
# there is only one study with 4 outcomes

# use metafor rma to compute the mean LOR using Fixed effects

FFTyoLrma <- rma(yi = LOR,
                 vi = varLOR,
                 data = LORyouth,
                 method = "FE")
FFTyoLrma
```

```
Fixed-Effects Model (k = 4)

I^2 (total heterogeneity / total variability):   0.00%
H^2 (total variability / sampling variability):  0.71

Test for Heterogeneity:
Q(df = 3) = 2.1339, p-val = 0.5451

Model Results:

estimate      se     zval    pval    ci.lb    ci.ub     
 -0.6934  0.2529  -2.7419  0.0061  -1.1891  -0.1977  ** 

---
Signif. codes:  0 '***' 0.001 '**' 0.01 '*' 0.05 '.' 0.1 ' ' 1
```

```
# convert results to odds ratio metric
# mean odds ratio
exp(FFTyoLrma$beta)
```

```
             [,1]
intrcpt 0.4998723
```

```
# SE in oR metric
exp(FFTyoLrma$se)
```

```
[1] 1.287741
```

```
# 95% lower bound
exp(FFTyoLrma$ci.lb)
```

```
[1] 0.3045084
```

```
# 95% upper bound
exp(FFTyoLrma$ci.ub)
```

```
[1] 0.8205761
```

### School LOR outcomes

Though there are two studies with school LORs, only one has an estimate. No analysis is possible. Study 52 does not provide sufficient information - only study 47 has an effect size.

```
# School LOR OUTCOMES

LORschool <- subset(FFTLOR, FFTLOR$Outcome == 9)

# Examine the number of outcomes and studies
table(LORschool$StudyID)
```

```
47 52 
 1  1
```

```
summary(LORschool$LOR)
```

```
   Min. 1st Qu.  Median    Mean 3rd Qu.    Max.    NA's 
-0.6574 -0.6574 -0.6574 -0.6574 -0.6574 -0.6574       1
```

## COMBINED ANALYSIS

The sections below convert the 60 LOR effect sizes to SMDs and conduct analyses on the combined data file.

```
# convert studies with LOR only to SMD

fftESall <- fftES

fftESall <-
  fftESall %>%
  mutate(
    SMD = if_else(is.na(SMD) == TRUE, LOR*(sqrt(3)/pi), SMD),
    varsmd = if_else(is.na(varsmd) == TRUE, varLOR*(3/(pi^2)), varsmd)
  )
```

### Overall SMD mean effect size using CE model

This analysis provides the overall SMD and prediction interval for the combined ES data. I provide a histogram to see the distribution of effect sizes and to check on the large LOR in study 49, outcome 16. In the SMD metric, the outlier is not as extreme.

```
# Estimate the mean effect size using RVE CE model

allESCE <- robu(formula = SMD ~ 1, data = fftESall,
                 studynum = StudyID, var.eff.size = varsmd,
                 rho = 0.8, small = TRUE)
allESCE
```

```
RVE: Correlated Effects Model with Small-Sample Corrections 

Model: SMD ~ 1 

Number of studies = 15 
Number of outcomes = 293 (min = 2 , mean = 19.5 , median = 8 , max = 76 )
Rho = 0.8 
I.sq = 68.24988 
Tau.sq = 0.08246667 

               Estimate StdErr t-value  dfs P(|t|>) 95% CI.L 95% CI.U Sig
1 X.Intercept.     0.19 0.0949    2.01 12.5  0.0669  -0.0154    0.396   *
---
Signif. codes: < .01 *** < .05 ** < .10 *
---
Note: If df < 4, do not trust the results
```

```
# check sensitivity to values of rho
sensitivity(allESCE)
```

```
RVE: Correlated Effects Model with Small-Sample Corrections 
Model: SMD ~ 1 

Sensitivity Analysis 

                          Rho = 0 Rho = 0.2 Rho = 0.4 Rho = 0.6 Rho = 0.8
 X.Intercept. Coefficient 0.1899  0.1901    0.1902    0.1904    0.1905   
              Std. Error  0.0948  0.0949    0.0949    0.0949    0.0949   
 Tau.sq       Estimate    0.0811  0.0814    0.0818    0.0821    0.0825   
 Rho = 1
 0.1906 
 0.0949 
 0.0828
```

```
# compute the 95% prediction interval
lbPIall <- allESCE$reg_table$b.r - 1.96*sqrt(allESCE$mod_info$tau.sq)
ubPIall <- allESCE$reg_table$b.r + 1.96*sqrt(allESCE$mod_info$tau.sq)

# lower bound
lbPIall
```

```
           [,1]
[1,] -0.3723548
```

```
# upper bound
ubPIall
```

```
         [,1]
[1,] 0.753352
```

```
hist(fftESall$SMD)
```

### Moderator Analysis with Combined Outcomes: US vs other Countries

The code below examines the difference between US and other countries. The first code chunk examines the difference between US and other countries; the difference is not statistically significant. The second code chunk provides the means for the two groups.

```
# get table for US vs. non-US
table(fftESall$US)
```

```
  0   1 
 62 244
```

```
# MR model for US versus other countries
USMRCE <- robu(formula = SMD ~ US, 
               data = fftESall,
                 studynum = StudyID, var.eff.size = varsmd,
                 rho = 0.8, small = TRUE)
USMRCE
```

```
RVE: Correlated Effects Model with Small-Sample Corrections 

Model: SMD ~ US 

Number of studies = 15 
Number of outcomes = 293 (min = 2 , mean = 19.5 , median = 8 , max = 76 )
Rho = 0.8 
I.sq = 70.12083 
Tau.sq = 0.09314218 

               Estimate StdErr t-value  dfs P(|t|>) 95% CI.L 95% CI.U Sig
1 X.Intercept.    0.142  0.341   0.416 1.98   0.718    -1.34     1.62    
2           US    0.067  0.354   0.189 3.16   0.861    -1.03     1.16    
---
Signif. codes: < .01 *** < .05 ** < .10 *
---
Note: If df < 4, do not trust the results
```

```
# MR analysis to get separate means
USMRCE2 <- robu(formula = SMD ~ -1 + factor(US), 
               data = fftESall,
                 studynum = StudyID, var.eff.size = varsmd,
                 rho = 0.8, small = TRUE)
USMRCE2
```

```
RVE: Correlated Effects Model with Small-Sample Corrections 

Model: SMD ~ -1 + factor(US) 

Number of studies = 15 
Number of outcomes = 293 (min = 2 , mean = 19.5 , median = 8 , max = 76 )
Rho = 0.8 
I.sq = 70.12083 
Tau.sq = 0.09314218 

              Estimate StdErr t-value  dfs P(|t|>) 95% CI.L 95% CI.U Sig
1 factor.US.0    0.142 0.3414   0.416 1.98  0.7183 -1.34005    1.624    
2 factor.US.1    0.209 0.0925   2.258 9.70  0.0483  0.00191    0.416  **
---
Signif. codes: < .01 *** < .05 ** < .10 *
---
Note: If df < 4, do not trust the results
```

### Moderator Analysis with Combined Outcomes: Developer involved or not

Note that US and developer-involved are confounded. All the developer-involved studies are in the US.

```
# frequencies for Us versus other countries
table(fftESall$developers)
```

```
  0   1 
282  24
```

```
# Is US and developers confounded?
table(fftESall$developers, fftESall$US)
```

```
      0   1
  0  62 220
  1   0  24
```

```
# MR model for US versus other countries
DVMRCE <- robu(formula = SMD ~ developers, 
               data = fftESall,
                 studynum = StudyID, var.eff.size = varsmd,
                 rho = 0.8, small = TRUE)
DVMRCE
```

```
RVE: Correlated Effects Model with Small-Sample Corrections 

Model: SMD ~ developers 

Number of studies = 15 
Number of outcomes = 293 (min = 2 , mean = 19.5 , median = 8 , max = 76 )
Rho = 0.8 
I.sq = 69.90115 
Tau.sq = 0.09599543 

               Estimate StdErr t-value  dfs P(|t|>) 95% CI.L 95% CI.U Sig
1 X.Intercept.    0.155  0.101   1.535 8.21   0.162  -0.0769    0.387    
2   developers    0.122  0.242   0.503 8.30   0.628  -0.4336    0.678    
---
Signif. codes: < .01 *** < .05 ** < .10 *
---
Note: If df < 4, do not trust the results
```

```
# MR analysis to get separate means
DVMRCE2 <- robu(formula = SMD ~ -1 + factor(developers), 
               data = fftESall,
                 studynum = StudyID, var.eff.size = varsmd,
                 rho = 0.8, small = TRUE)
USMRCE2
```

```
RVE: Correlated Effects Model with Small-Sample Corrections 

Model: SMD ~ -1 + factor(US) 

Number of studies = 15 
Number of outcomes = 293 (min = 2 , mean = 19.5 , median = 8 , max = 76 )
Rho = 0.8 
I.sq = 70.12083 
Tau.sq = 0.09314218 

              Estimate StdErr t-value  dfs P(|t|>) 95% CI.L 95% CI.U Sig
1 factor.US.0    0.142 0.3414   0.416 1.98  0.7183 -1.34005    1.624    
2 factor.US.1    0.209 0.0925   2.258 9.70  0.0483  0.00191    0.416  **
---
Signif. codes: < .01 *** < .05 ** < .10 *
---
Note: If df < 4, do not trust the results
```

### Moderator Analysis with Independent/non-US, Independent US and Developer -involved

For this analysis, I created a new code with three levels: 1 = Independent researchers outside of the US, 2 = Independent researchers within the US, and 3 = Developers in the US. The analysis treats this as a factor, and in the first meta-regression treats level 1 (Independent researchers outside of the US) as the reference category. In the second analysis, the meta-regression results provide the means for the three groups.

```
# create two new dummy codes for three groups
# non-US, US independent and developers
# developers is already a dummy code
# create dummy code for independent US
fftESall <-
  fftESall %>%
  mutate(
    indepUS = case_when(US == 0 & developers == 0 ~ 1,
                        US == 1 & developers == 0 ~ 2,
                        US == 1 & developers == 1 ~ 3)
    )

table(fftESall$indepUS)
```

```
  1   2   3 
 62 220  24
```

```
# MR analysis to compare US independent and developer with nonUS
DVMRCE3 <- robu(formula = SMD ~ factor(indepUS), 
               data = fftESall,
                 studynum = StudyID, var.eff.size = varsmd,
                 rho = 0.8, small = TRUE)
DVMRCE3
```

```
RVE: Correlated Effects Model with Small-Sample Corrections 

Model: SMD ~ factor(indepUS) 

Number of studies = 15 
Number of outcomes = 293 (min = 2 , mean = 19.5 , median = 8 , max = 76 )
Rho = 0.8 
I.sq = 71.76466 
Tau.sq = 0.112628 

                   Estimate StdErr t-value  dfs P(|t|>) 95% CI.L 95% CI.U Sig
1     X.Intercept.   0.1455  0.344   0.423 1.99   0.714   -1.345    1.636    
2 factor.indepUS.2   0.0191  0.348   0.055 3.98   0.959   -0.949    0.988    
3 factor.indepUS.3   0.1402  0.408   0.344 4.52   0.747   -0.943    1.223    
---
Signif. codes: < .01 *** < .05 ** < .10 *
---
Note: If df < 4, do not trust the results
```

```
# MR analysis to get separate means US independent, nonUS and developer
DVMRCE4 <- robu(formula = SMD ~ -1 + factor(indepUS), 
               data = fftESall,
                 studynum = StudyID, var.eff.size = varsmd,
                 rho = 0.8, small = TRUE)
DVMRCE4
```

```
RVE: Correlated Effects Model with Small-Sample Corrections 

Model: SMD ~ -1 + factor(indepUS) 

Number of studies = 15 
Number of outcomes = 293 (min = 2 , mean = 19.5 , median = 8 , max = 76 )
Rho = 0.8 
I.sq = 71.76466 
Tau.sq = 0.112628 

                   Estimate StdErr t-value  dfs P(|t|>) 95% CI.L 95% CI.U Sig
1 factor.indepUS.1    0.145 0.3441   0.423 1.99   0.714   -1.345    1.636    
2 factor.indepUS.2    0.165 0.0522   3.153 5.35   0.023    0.033    0.296  **
3 factor.indepUS.3    0.286 0.2191   1.304 4.37   0.257   -0.303    0.874    
---
Signif. codes: < .01 *** < .05 ** < .10 *
---
Note: If df < 4, do not trust the results
```

### Moderator Analysis with Combined Outcomes: RCT versus QED

This code chunk compares the RCTs with QEDs. The first analysis provides the difference between RCTs and the reference category, QED. The second analysis provides the means for both groups of studies.

```
# frequencies of RCT versus not
table(fftESall$RCT)
```

```
  0   1 
 44 262
```

```
# MR model for US versus other countries
RCTMRCE <- robu(formula = SMD ~ RCT, 
               data = fftESall,
                 studynum = StudyID, var.eff.size = varsmd,
                 rho = 0.8, small = TRUE)
RCTMRCE
```

```
RVE: Correlated Effects Model with Small-Sample Corrections 

Model: SMD ~ RCT 

Number of studies = 15 
Number of outcomes = 293 (min = 2 , mean = 19.5 , median = 8 , max = 76 )
Rho = 0.8 
I.sq = 67.80011 
Tau.sq = 0.08863433 

               Estimate StdErr t-value  dfs P(|t|>) 95% CI.L 95% CI.U Sig
1 X.Intercept.   0.0571  0.100   0.571 3.72   0.601   -0.229    0.343    
2          RCT   0.2348  0.174   1.351 9.00   0.210   -0.158    0.628    
---
Signif. codes: < .01 *** < .05 ** < .10 *
---
Note: If df < 4, do not trust the results
```

```
# MR analysis to get separate means
RCTMRCE2 <- robu(formula = SMD ~ -1 + factor(RCT), 
               data = fftESall,
                 studynum = StudyID, var.eff.size = varsmd,
                 rho = 0.8, small = TRUE)
RCTMRCE2
```

```
RVE: Correlated Effects Model with Small-Sample Corrections 

Model: SMD ~ -1 + factor(RCT) 

Number of studies = 15 
Number of outcomes = 293 (min = 2 , mean = 19.5 , median = 8 , max = 76 )
Rho = 0.8 
I.sq = 67.80011 
Tau.sq = 0.08863433 

               Estimate StdErr t-value  dfs P(|t|>) 95% CI.L 95% CI.U Sig
1 factor.RCT.0   0.0571  0.100   0.571 3.72  0.6007  -0.2290    0.343    
2 factor.RCT.1   0.2919  0.142   2.054 8.34  0.0726  -0.0335    0.617   *
---
Signif. codes: < .01 *** < .05 ** < .10 *
---
Note: If df < 4, do not trust the results
```

## COMBINED ANALYSIS BY OUTCOME

This section reports on the analysis by outcome, using the combined ES data.

### Placement Outcomes

The first analysis uses RVE with a correlated effects model. Though there are four studies and 18 ES, the dfs in the CE analysis are 2.88. The second analysis uses a fixed effects model with study as a moderator, and treating all studies as independent - this analysis is conducted to produce a forest plot of the 18 ES. The third analysis aggregates the ES to the level of the study (assuming the correlation among effect sizes within studies is 0.8), and estimates the fixed effects mean across the four studies. The forest plot is also provided for this analysis.

```
# get the table of all outcomes using combined ES data set
table(fftESall$Outcome)
```

```
 1  2  3  4  5  6  7  8  9 
19 48 39 16 16 79 24 62  3
```

```
# get a data set with only placement outcomes
placement <- subset(fftESall, fftESall$Outcome == 1)

# use robumeta to estimate the mean effect size

PlCE <- robu(formula = SMD ~ 1, data = placement,
                 studynum = StudyID, var.eff.size = varsmd,
                 rho = 0.8, small = TRUE)
PlCE
```

```
RVE: Correlated Effects Model with Small-Sample Corrections 

Model: SMD ~ 1 

Number of studies = 4 
Number of outcomes = 18 (min = 2 , mean = 4.5 , median = 4.5 , max = 7 )
Rho = 0.8 
I.sq = 45.45485 
Tau.sq = 0.05535046 

               Estimate StdErr t-value  dfs P(|t|>) 95% CI.L 95% CI.U Sig
1 X.Intercept.    0.133  0.109    1.22 2.88   0.312   -0.221    0.488    
---
Signif. codes: < .01 *** < .05 ** < .10 *
---
Note: If df < 4, do not trust the results
```

```
# check sensitivity to values of rho
sensitivity(PlCE)
```

```
RVE: Correlated Effects Model with Small-Sample Corrections 
Model: SMD ~ 1 

Sensitivity Analysis 

                          Rho = 0 Rho = 0.2 Rho = 0.4 Rho = 0.6 Rho = 0.8
 X.Intercept. Coefficient 0.1338  0.134     0.1335    0.1333    0.1332   
              Std. Error  0.1069  0.107     0.1079    0.1084    0.1088   
 Tau.sq       Estimate    0.0429  0.046     0.0491    0.0522    0.0554   
 Rho = 1
 0.1330 
 0.1092 
 0.0585
```

```
# compute the 95% prediction interval
lbPIplace <- PlCE$reg_table$b.r - 1.96*sqrt(PlCE$mod_info$tau.sq)
ubPIplace <- PlCE$reg_table$b.r + 1.96*sqrt(PlCE$mod_info$tau.sq)

#Lower bound
lbPIplace
```

```
          [,1]
[1,] -0.327964
```

```
# Upper bound
ubPIplace
```

```
          [,1]
[1,] 0.5942819
```

```
# sort data by StudyID to facilitate the forest plot
placement <- placement[order(placement$StudyID),]

# use metafor to conduct a fixed effects analysis with
# study as a moderator

Plrma <- rma(yi = SMD,
                vi = varsmd,
                data = placement, 
                mods = ~ -1 + factor(StudyID),
                method = "FE")
```

```
Warning: Studies with NAs omitted from model fitting.
```

```
Warning: Redundant predictors dropped from the model.
```

```
Plrma
```

```
Fixed-Effects with Moderators Model (k = 18)

I^2 (residual heterogeneity / unaccounted variability): 28.32%
H^2 (unaccounted variability / sampling variability):   1.39

Test for Residual Heterogeneity:
QE(df = 14) = 19.5299, p-val = 0.1457

Test of Moderators (coefficients 1:4):
QM(df = 4) = 39.3736, p-val < .0001

Model Results:

                   estimate      se     zval    pval    ci.lb   ci.ub      
factor(StudyID)20   -0.2242  0.1414  -1.5856  0.1128  -0.5013  0.0529      
factor(StudyID)47    0.1550  0.0720   2.1518  0.0314   0.0138  0.2961    * 
factor(StudyID)49    0.2914  0.1723   1.6911  0.0908  -0.0463  0.6292    . 
factor(StudyID)50    0.5259  0.0970   5.4193  <.0001   0.3357  0.7161  *** 

---
Signif. codes:  0 '***' 0.001 '**' 0.01 '*' 0.05 '.' 0.1 ' ' 1
```

```
# forest plot to look at these seven ES
forest(Plrma, slab = placement$StudyID)
```

```
# Dfs for RVE are less than 4
# will use aggregate function in metafor to aggregate to level of study
# will assume a constant correlation of 0.8 among ES within studies

# turn placement data frame to an escalc object
placement <- escalc(yi = SMD, vi = varsmd, data = placement)

# average effect sizes
placeagg <- aggregate(placement, cluster = StudyID, rho = 0.8)

# use a fixed effects model to examine placement ES
placeFE <- rma(yi = SMD,
               vi = varsmd,
               data = placeagg,
               method = "FE")

placeFE
```

```
Fixed-Effects Model (k = 4)

I^2 (total heterogeneity / total variability):   0.00%
H^2 (total variability / sampling variability):  0.78

Test for Heterogeneity:
Q(df = 3) = 2.3325, p-val = 0.5063

Model Results:

estimate      se    zval    pval    ci.lb   ci.ub    
  0.1382  0.1227  1.1267  0.2599  -0.1022  0.3786    

---
Signif. codes:  0 '***' 0.001 '**' 0.01 '*' 0.05 '.' 0.1 ' ' 1
```

```
# forest plot for aggregated analysis
forest(placeFE, slab = placeagg$StudyID)
```

### Arrest Outcomes

This analysis has 8 studies and 46 outcomes. The CE model with RVE is appropriate here. In the SMD metric, there are no obvious outliers.

```
### ARREST OUTCOMES
arrest <- subset(fftESall, fftESall$Outcome == 2)

# use robumeta to estimate the mean effect size

ArCE <- robu(formula = SMD ~ 1, data = arrest,
                 studynum = StudyID, var.eff.size = varsmd,
                 rho = 0.8, small = TRUE)
ArCE
```

```
RVE: Correlated Effects Model with Small-Sample Corrections 

Model: SMD ~ 1 

Number of studies = 8 
Number of outcomes = 46 (min = 2 , mean = 5.75 , median = 2.5 , max = 22 )
Rho = 0.8 
I.sq = 74.91385 
Tau.sq = 0.08582561 

               Estimate StdErr t-value  dfs P(|t|>) 95% CI.L 95% CI.U Sig
1 X.Intercept.    0.182  0.141     1.3 6.31    0.24   -0.158    0.523    
---
Signif. codes: < .01 *** < .05 ** < .10 *
---
Note: If df < 4, do not trust the results
```

```
# check sensitivity to values of rho
sensitivity(ArCE)
```

```
RVE: Correlated Effects Model with Small-Sample Corrections 
Model: SMD ~ 1 

Sensitivity Analysis 

                          Rho = 0 Rho = 0.2 Rho = 0.4 Rho = 0.6 Rho = 0.8
 X.Intercept. Coefficient 0.182   0.1818    0.1820    0.1823    0.1825   
              Std. Error  0.140   0.1404    0.1405    0.1406    0.1406   
 Tau.sq       Estimate    0.084   0.0844    0.0849    0.0854    0.0858   
 Rho = 1
 0.1827 
 0.1407 
 0.0863
```

```
# compute the 95% prediction interval
lbPIarrest <- ArCE$reg_table$b.r - 1.96*sqrt(ArCE$mod_info$tau.sq)
ubPIarrest <- ArCE$reg_table$b.r + 1.96*sqrt(ArCE$mod_info$tau.sq)

#Lower bound
lbPIarrest
```

```
           [,1]
[1,] -0.3917062
```

```
# Upper bound
ubPIarrest
```

```
          [,1]
[1,] 0.7566973
```

```
hist(arrest$SMD)
```

### Youth Behavior

This analysis has seven studies and 74 outcomes. The CE model with RVE is appropriate here.

```
### YOUTH BEHAVIOR SYMPTOMS OUTCOMES
youth <- subset(fftESall, fftESall$Outcome == 6)

# use robumeta to estimate the mean effect size

ybCE <- robu(formula = SMD ~ 1, data = youth,
                 studynum = StudyID, var.eff.size = varsmd,
                 rho = 0.8, small = TRUE)
ybCE
```

```
RVE: Correlated Effects Model with Small-Sample Corrections 

Model: SMD ~ 1 

Number of studies = 7 
Number of outcomes = 74 (min = 3 , mean = 10.6 , median = 12 , max = 19 )
Rho = 0.8 
I.sq = 16.36013 
Tau.sq = 0.01173815 

               Estimate StdErr t-value  dfs P(|t|>) 95% CI.L 95% CI.U Sig
1 X.Intercept.  -0.0293 0.0665   -0.44 4.56    0.68   -0.206    0.147    
---
Signif. codes: < .01 *** < .05 ** < .10 *
---
Note: If df < 4, do not trust the results
```

```
# check sensitivity to values of rho
sensitivity(ybCE)
```

```
RVE: Correlated Effects Model with Small-Sample Corrections 
Model: SMD ~ 1 

Sensitivity Analysis 

                          Rho = 0  Rho = 0.2 Rho = 0.4 Rho = 0.6 Rho = 0.8
 X.Intercept. Coefficient -0.03034 -0.03009  -0.02984  -0.0296   -0.0293  
              Std. Error   0.06573  0.06594   0.06615   0.0664    0.0665  
 Tau.sq       Estimate     0.00559  0.00713   0.00867   0.0102    0.0117  
 Rho = 1
 -0.0290
  0.0667
  0.0133
```

```
# compute the 95% prediction interval
lbPIyouth <- ybCE$reg_table$b.r - 1.96*sqrt(ybCE$mod_info$tau.sq)
ubPIyouth <- ybCE$reg_table$b.r + 1.96*sqrt(ybCE$mod_info$tau.sq)

#Lower bound
lbPIyouth
```

```
           [,1]
[1,] -0.2416642
```

```
# Upper bound
ubPIyouth
```

```
          [,1]
[1,] 0.1830395
```

### School Outcomes

In the combined ES analysis, there are two ES, both from the same study. A fixed effects model is reported with a forest plot.

```
# get school data set

school <- subset(fftESall, fftESall$Outcome == 9)

# there are only two effect sizes
# use a fixed effects model to get mean

schoolFE <- rma(yi = SMD,
                vi = varsmd,
                data = school,
                method = "FE")
```

```
Warning: Studies with NAs omitted from model fitting.
```

```
schoolFE
```

```
Fixed-Effects Model (k = 2)

I^2 (total heterogeneity / total variability):   0.00%
H^2 (total variability / sampling variability):  0.20

Test for Heterogeneity:
Q(df = 1) = 0.1997, p-val = 0.6550

Model Results:

estimate      se     zval    pval    ci.lb   ci.ub    
 -0.1581  0.1720  -0.9188  0.3582  -0.4953  0.1791    

---
Signif. codes:  0 '***' 0.001 '**' 0.01 '*' 0.05 '.' 0.1 ' ' 1
```

```
# forest plot
forest(schoolFE, slab = school$StudyID)
```
